# Protocol for CHANGE: a randomized clinical trial assessing lifestyle coaching plus care coordination versus care coordination alone versus treatment as usual to reduce risks of cardiovascular disease in adults with schizophrenia and abdominal obesity

**DOI:** 10.1186/s12888-015-0465-2

**Published:** 2015-05-23

**Authors:** Helene Speyer, Hans Christian Brix Nørgaard, Carsten Hjorthøj, Thomas Axel Madsen, Søren Drivsholm, Charlotta Pisinger, Christian Gluud, Ole Mors, Jesper Krogh, Merete Nordentoft

**Affiliations:** 1 Mental Health Centre Copenhagen, Mental Health Services in the Capital Region, DK-2400 Copenhagen, Denmark; 2 Institute of Clinical Medicine, Faculty of Health Sciences, University of Copenhagen, Copenhagen, Denmark; 3 Research Department P, Aarhus University Hospital, Risskov, Denmark; 4 Research Centre for Prevention and Health, Department 84–85, Glostrup University Hospital, Glostrup, Denmark; 5 Copenhagen Trial Unit, Centre for Clinical Intervention Research, Rigshospitalet, Copenhagen University Hospital, Copenhagen, Denmark

## Abstract

**Background:**

Life expectancy in patients with schizophrenia is reduced by 20 years for males and 15 years for females compared to the general population. About 60% of the excess mortality is due to physical illnesses, with cardiovascular disease being the single largest cause of death.

**Methods/design:**

The CHANGE trial is an investigator-initiated, independently funded, randomized, parallel-group, superiority, multi-centre trial with blinded outcome assessment. 450 patients aged 18 years or above, diagnosed with schizophrenia spectrum disorders and increased waist circumference, will be recruited and randomized 1:1:1 to 12-months interventions. We will compare the effects of 1) affiliation to the CHANGE team, offering a tailored, manual-based intervention targeting physical inactivity, unhealthy dietary habits, and smoking, and facilitating contact to their general practitioner to secure medical treatment of somatic comorbidity; versus 2) affiliation to a care coordinator who will secure guideline-concordant monitoring and treatment of somatic comorbidity by facilitating contact to their general practitioner; versus 3) treatment as usual to evaluate the potential add-on effects of lifestyle coaching plus care coordination or care coordination alone to treatment as usual. The primary outcome is the 10-year risks of cardiovascular disease assessed at 12 months after randomization.

**Discussion:**

The premature mortality observed in this vulnerable population has not formerly been addressed specifically by using composite surrogate outcomes for mortality. The CHANGE trial expands the evidence for interventions aiming to reduce the burden of metabolic disturbances with a view to increase life expectancy. Here, we present the trial design, describe the methodological concepts in detail, and discuss the rationale and challenges of the intermediate outcomes.

**Trial registration:**

Clinical Trials.gov NCT01585493. Date of registration 27^th^ of March 2012.

**Electronic supplementary material:**

The online version of this article (doi:10.1186/s12888-015-0465-2) contains supplementary material, which is available to authorized users.

## Background

Schizophrenia is a life shortening disease, with life expectancy being reduced by 20 years for males and 15 years for females compared to the general population [1]. About 60% of the excess mortality is due to physical illness, with cardiovascular disease being the single largest cause of death [2]. While the general population has benefitted from a steady decline in ischemic heart disease since the 1980s, this is not the case for patients with schizophrenia [3-5].

Death due to cardiovascular disease is closely related to metabolic syndrome [6]. It has been estimated that the prevalence of metabolic syndrome in patients with schizophrenia may be as high as one in three [7]. The high mortality due to cardiovascular disease can be explained by unhealthy lifestyle [8], disparities in quality of health care [9], metabolic adverse effects of antipsychotics [10], and probably genetic vulnerability [11]. Of these, lifestyle and use of primary health care might be considered modifiable factors and thus accessible to intervention.

Sedentary lifestyle, smoking, and unhealthy dietary habits are highly prevalent among patients with schizophrenia. A recent study found that patients with schizophrenia spend more than 12 hours on sedentary activities on a daily basis [12], and make unhealthy dietary choices, consuming more sugar and saturated fats than the background population [8]. The combination of pronounced sedentary behaviour and a diet rich in sugar and fat, highly contributes to the reported proportion of obesity of 42% to 60% among patients with schizophrenia [13]. A significant association between low aerobic fitness and metabolic syndrome has been found in patients with schizophrenia [14]. Furthermore, patients with schizophrenia have more than five times the odds of being smoker, and smoking cessation is lower than compared to the general population [15]. Thus, the high prevalence of cardiovascular disease is multifactorial, and likely requires a multifaceted intervention.

Several studies have examined the effect of behavioural and pharmacological interventions targeting single cardiovascular risk factors like obesity, smoking, glucose-intolerance, and dyslipidaemia in patients with schizophrenia [16-24]. Weight loss or prevention of weight gain has been studied in trials aiming to improve unhealthy diet, physical inactivity, or a combination. Two recent systematic reviews of randomized clinical trials of lifestyle interventions conclude that there is significant reduction of 0.94 kg/m^2^ [25] and 0.98 kg/m^2^ [26] in body mass index (BMI), the latter review finding a superior effect of combined nutritional counselling and exercise. This is supported by our own work [27], where exercise as a single intervention does not seem to affect BMI or other cardiovascular risk factors [28]. Further support for the effect of interventions combining exercise and nutrition has been found recently, in a randomized clinical trial for weight loss in patients with schizophrenia resulting in a net difference in BMI of 1.1 kg/m^2^ between patients in the intervention group and controls [29]. There is evidence that bupropion and varenicline increase the chance for smoking cessation in patients with schizophrenia [24,30,31], but no randomized clinical trial has combined smoking cessation with an exercise and nutritional interventions, to maximize the possibility to reduce cardiovascular disease.

Disparity in quality of primary health care is another major issue explaining the high mortality. The European Psychiatric Association [32] and the National Institute for Health and Care Excellence (NICE) guidelines both recommend that patients with schizophrenia are annually screened for obesity and cardiovascular risk factors, and receive guideline concordant prophylactic treatment of these factors, but this does not appear to happen [33]. Acknowledging the unmet need for primary health care among patients with schizophrenia, several approaches have been proposed to fill the gap; an expanded role for the psychiatrist, an integrative care model with a general practitioner allocated to supported housings or care coordination providing contact to primary care. Reviewing the literature in the electronic databases (PubMed, EMBASE, and Clinical Trials.gov) for studies related to the terms “*shared care*, *collaborative care* and *care coordination*” and “*SMI* (severe mental illnesses) and/or *schizophrenia*” resulted in no published studies that have examined the effect of care coordination on schizophrenia patients in a randomized clinical trial. We found one ongoing trial assessing the effect of care management with quality of life as the primary outcome and cardiovascular risk factors as the secondary outcome [34]. No results from that trial have yet been published [34].

Our systematic search revealed no trials or studies investigating the add-on effect of lifestyle interventions compared with care coordination alone in a randomized clinical trial.

### Aim and hypothesis

We will compare in a randomized clinical trial the benefits and harms of 1) lifestyle coaching defined as affiliation to a CHANGE team member, offering a tailored, manual-based intervention targeting physical inactivity, unhealthy dietary habits, smoking, and facilitate contact to their general practitioner to secure medical treatment of somatic comorbidity; versus 2) affiliation to a care coordinator who will secure guideline-concordant monitoring and treatment of somatic comorbidity by facilitating contact to their general practitioner; versus 3) treatment as usual for obese patients with schizophrenia. The primary outcome of the CHANGE trial is the estimated 10-years risk of cardiovascular at 12 months post-randomization. Our alternative hypotheses are that there will be a reduction in the estimated 10-years risk of cardiovascular disease in the two experimental intervention groups compared with the control group, and that the lifestyle coaching will be more effective than the care-coordination.

The duration of all interventions is 12 months. Assessment of outcomes will take place 12 months and 24 months after randomization.

## Method

### Design

The CHANGE trial is an investigator-initiated, independently funded, randomized, parallel-group, superiority, multi-centre trial with blinded outcome assessment.

### Patients

Patients were recruited from well-defined catchment areas in two major Danish cities (Aarhus and Copenhagen). Eligible patients were verbally informed by the usual caretaker, and referred to CHANGE research staff by phone or e-mail, if accepting. The patients were contacted by phone, and a meeting was arranged at the research centre, the outpatient clinic, or at the patient’s home. Verbal and written information was provided. If the patient accepted participation in the trial, an informed consent was signed and an appointment for collection of baseline data was made. Baseline data were collected between *1*^*st*^ of December 2012 and *1*^*st*^ of May 2014.

#### Patient inclusion criteria

1) Adults, ≥18 years, fulfilling the ICD-10 diagnostic criteria for schizophrenia, persistent delusional disorders, or schizoaffective disorders [35] using the Schedule for Clinical Assessment in Neuropsychiatry (SCAN) [36]; 2) Waist circumference ≥88 cm for females and ≥102 cm for males [37] measured between the crista iliac and lowest rib); and 3) Written informed consent.

#### Patient exclusion criteria

1. Current self-reported pregnancy
2. Inability to consent.

### Randomization and blinding

Patients were randomized with a 1:1:1 ratio to either the lifestyle coaching versus care coordination versus treatment as usual. Randomization was stratified according to the two psychiatric centres, sex, and a high/low risk of cardiovascular disease. High risk was defined according to cut-off points from a Danish population study using the Copenhagen risk score, aiming to identify the quintile at highest risk. Each person was - in the computer program - simulated as 60 years old, to reach a substantial level of risk [38], This approach is was recommended by the European cardiovascular risk factor management guidelines to asses risk in young individuals [39].

The randomization was centralized and carried out by the Copenhagen Trial Unit using a computerized randomization sequence with alternating block sizes unknown to the investigators. After inclusion in the trial, a health care provider contacted the Copenhagen Trial Unit with a unique patient identifier plus stratification variables and in return received the patient allocation.

### Blinding

Outcome assessors, statisticians, and all investigators involved in the trial are blinded to patient allocation. Patients and the health professionals providing the interventions are not blinded to patient allocation. The statistical analysis of the 12 months post randomization follow up and the drafting of the first result manuscript will be carried out blinded to patient allocation.

### Interventions

#### Lifestyle coaching

The theoretical framework of the lifestyle coaching was based on the theory of stages of change [40], motivational interviewing (MI), and an assertive approach adapted from the assertive community treatment [41]. MI is a method to help patients elicit their own wishes to change, and it has been shown effective in patients with schizophrenia and comorbid alcohol abuse [42]. The assertive approach allows the staff to be respectfully active and still persistent in follow-up; be flexible in time; and conduct short message services, phone calls, home visits or meetings in the local area.

Manuals (see Additional file 1: care coordinator manual, Additional file 2: diet manual and Additional file 3: physical activity manual (Danish)): The three methods mentioned above, were incorporated in four manuals with detailed descriptions of the intervention addressing care-coordination, smoking cessation, healthy diet, and increased physical activity, based on the official Danish guidelines [43,44]. An important first step was to clarify possibilities for changes that seem achievable and realistic according to the stages of change. The aim of the lifestyle coach was to support the patient in setting up individual goals that pay attention to the patient’s values, life conditions, and priorities. The coach offered home visits with systematic exploration of possibilities for physical activity in daily life, which were realistic and attractive to the patient. Dietary changes require concrete examination of the patient’s dietary habits, food purchases and cooking practices, and identification of economically realistic, easy and attractive possibilities for change. During home visits, the coach took part in the activities (ex. physical activity or food purchases) if requested by the patient, to support lifestyle changes. Personal and professional networks and patient network could be part of individual plans. The smoking cessation program was adapted from the program published by The National Cancer Organization [45,46], and tailored to the patient population in order to elicit and enhance motivation and maintain smoking cessation. Support was provided for motivation, including prevention of relapses, and smoking cessation medication. First line treatment was nicotine substitution and second line was bupropion.

The staff had access to anthropometric measures and blood samples collected at baseline and used these in their first consultation with patients to plan the further course. Weight was monitored every third month.

Patients commenced the lifestyle coaching as soon as possible after collection of baseline data, even if they were in-patients. The coach:patient ratio was 1:15. To allow sufficient time to implement changes in habits, each patient was offered affiliation with the team member for one year and we offered a follow-up after 24 months, to investigate whether changes in lifestyle and treatment of physical disorders were maintained one year after the intervention ended. The lifestyle coach aimed to have individual meetings or activities with their patients weekly. Further support was provided by phone calls, e-mails, and text messages.

The lifestyle coaches and care coordinators performed written registration of all contact with patients including cancellations and classification of the focus area of each consultation, enabling the researchers to evaluate adherence and program fidelity.

Training and supervision: Lifestyle coaches were health professionals (e.g., occupational therapist, physiotherapists, or dieticians) with clinical training in psychiatry. They received a 5-days course in motivational interviewing, a 5-days course in smoking cessation, a 1-day course in examination and treatment of lifestyle disorders and a 2-days course on healthy dieting, based on the official Danish guidelines. During the intervention, supervision of the team took place weekly. In addition to the intervention described above, the patients were offered care coordination (see below) and treatment as usual.

#### Care coordinator function

**Figure 1:**
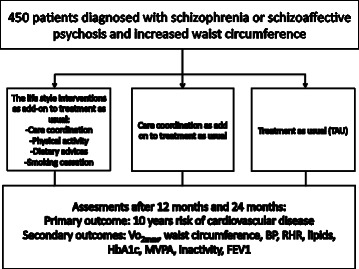
10 years risk of CVD estimated with Copenhagen Risk Score, Vo2max = max. oxygen uptake, BP = blood pressure, RHR = resting heart rate, HbA1c = glycosylated haemoglobin, MVPA = moderate/vigorous activity, inactivity = sedentary activity, FEV1 = forced expiratory volume.

**Figure 2:**
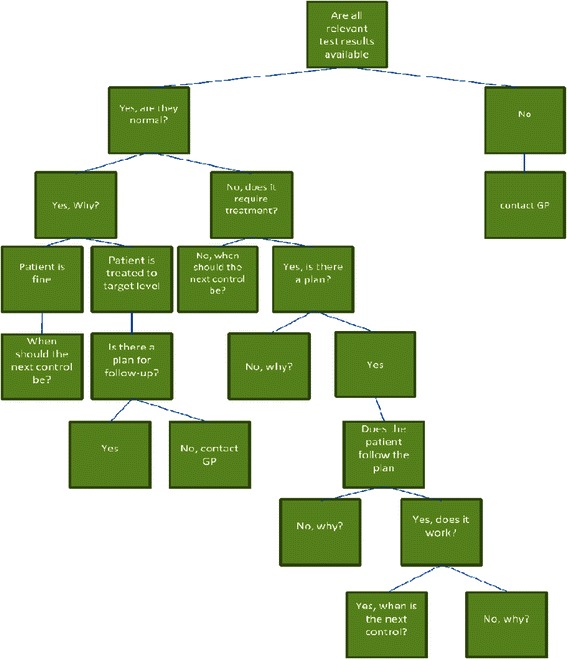
The decision tree incorporated in the care coordinator function.

The care coordinator function was incorporated in the lifestyle intervention as well as the add on treatment in the second intervention group (see Figure 1). The care coordinator facilitated contact to primary care in order to ensure treatment of physical health problems. The care-coordinator was nurse with a nurse:patient ratio of 1:25. Affiliation to the care-coordinator was offered for one year. The intervention was manual-based, and the aim was to ensure that the patients in this group were monitored and received guideline-concordant medical treatment. Their contact with patients comprised personal meetings, phone calls and text messages, and the frequency of contact was adjusted according to the individual need. The first meeting with the patient consisted of a general health talk about the physical well-being and test results from physical examination performed at baseline. Special awareness was paid to symptoms of obstructive pulmonary disease, diabetes and cardiovascular disease. The care coordinator used the decision tree (Figure 2) to plan the further course. In addition to the care coordinator intervention described above, the patients continued treatment as usual.

#### Treatment as usual

In Denmark all persons have a personal general practitioner and can consult her/him for free when needed. Patients in secondary mental health services stay affiliated with their general practitioner, who is responsible for treating abnormal results from the mandatory yearly screening of metabolic risk factors. No formalized extra effort was made regarding lifestyle counselling or treatment of physical disorders. Results from the baseline assessment were available if requested by the patient or usual caretakers, and if any of the results was a matter of urgent consideration, the CHANGE research staff contacted the usual caretaker.

### Outcomes

Research staff blinded to patient allocation assesses outcomes. All patients will be assessed at the following time points: baseline (T0), 12 months post-randomization (T1-at completion of intervention), and 24 months post-randomization (T2).

### Study objectives

The CHANGE trial aims to answer the questions set out below under primary objectives, secondary objectives and exploratory objectives.

### Primary objectives

1. Is lifestyle coaching plus care coordination more effective than treatment as usual in reducing risk of cardiovascular disease 12 months from randomisation?
2. Is lifestyle coaching plus care coordination more effective than care coordination alone in reducing risk of cardiovascular disease 12 months from randomisation?
3. Is care coordination alone more effective than treatment as usual in reducing risk of cardiovascular disease 12 months from randomisation?

#### Primary outcome

The primary outcome is the risk of cardiovascular disease at 12 months, assessed by the Copenhagen risk score. The Copenhagen risk score is based on data from two large epidemiological studies in the Copenhagen area [47].

A risk assessment computer program (PRECARD®) combines the Copenhagen risk score with data from randomized clinical trials [47]. This composite measure includes: sex, family history of CVD (defined as parents suffering fatal or non-fatal cardiovascular event before the age of 55 years (father) or 60 years (mother); prior heart disease (defined as myocardial infarction (MI) or verified atherosclerosis of coronary arteries); +/- smoking; +/- diabetes mellitus (HbA1c-based or receiving anti glycaemic drugs); total cholesterol, high density lipoprotein cholesterol (HDL); systolic blood pressure; and body mass index (weight/height^2^). Absolute risk is defined as the probability of a clinical event (IHD, MI, stroke, death) happening to a person within 10 years. Age is simulated to be 60 years, to reach a substantial level of risk [38], aiming to estimate life time risk.

#### Secondary outcomes

Cardiorespiratory fitness was originally defined as an exploratory outcome, due to insecurity of the acceptability and feasibility of the test procedure among the recruited patients. After completed data collection at baseline, we found an acceptable level of satisfying tests, and redefined fitness to a key secondary outcome. The patient’s maximal oxygen uptake (V · O_2max_) ml oxygen/kg/min was measured using a bicycle cardiopulmonary exercise test. The test was based on L. B. Andersens cycle exercise protocol where the initial 5 min of the cycle test (Monark) the workload is 75 W for women, and 100 W for men (L. B. [48]). Then the workload is increased by 25 W/2 min till exhaustion. All patients were continuously verbally encouraged. The maximum pulse at VO_2max_ was recorded. Forced expiratory volume (FEV1) measured with Easyone® spirometer.

Physical Activity Scale was used to determine time spent on moderate and vigorous and sedentary activity a day [49]. Waist circumference measured between the crista iliac and lowest rib, blood pressure measured on the right upper arm after 10 minutes of rest in a sitting position - the average of the two last consecutive measurements will be reported, resting heart rate after 10 minutes of rest, HDL, non-HDL-cholesterol and HbA1c.

#### Exploratory outcomes

Anthropometric measures: weight in kg and body mass index, skinfolds measured at four sites (biceps, triceps, subscapular, suprailiac), and body fat percentage calculated from skinfold measures [50].

Psychometric measures: positive and negative symptoms (SAPS and SANS) [51], cognition (BACS) [52], quality of life (MANSA and EQ-5D) [53], global assessment of functioning (GAF) [54], perceived health [55], and perceived stress [56].

Biomedical status measures: triglycerides, high sensitive CRP (hsCRP), low-density lipoprotein cholesterol (LDL).

Lifestyle measures: food frequency questionnaire [57], 24 hour recall, self-reported point abstinence from smoking (nicotine dependence questionnaire [58]).

### Baseline measures

At baseline, the following was assessed: socio-demographic data; age, sex, self-reported ethnicity, marital status, economic status, work situation, and educational level. Health care: medical history of diabetes, cardiovascular disease, cerebrovascular disease, and other past medical history. Current medication.

Data regarding vital status, causes of death, use of health services, institutional stay, use of medication and use of services from general practice will be extracted from longitudinal Danish registers [59-62]; The Danish National Health Insurance Service Registry (NHSR) which holds information on all contacts to general practice and all services provided [63]; and The Danish Civil Registration System (CRS), which has updated information on vital status, e.g. day of death, on all Danish citizens. The register is a key tool in Danish epidemiologic research [64].

### Statistical analyses

#### Sample size

We expect the experimental interventions to reduce the Copenhagen risk score during 12 months from baseline by 2.5% 10-year risk for coronary heart disease in patients allocated to lifestyle coaching compared with the score in patients allocated to care coordination alone, and a similar reduction of 2.5% in care coordination compared to treatment as usual as presented in Table 1. We plan to compare all three groups and accordingly we reduced our alpha level to 0.05/3 = 0.0166 [65]. Allowing a power of 90% we need to recruit 150 patients to each intervention group for a total of 450 patients. This calculation is based on an SD of 5.9% of the Copenhagen risk score as found in the Inter99-investigation [38].

**Table 1:** **10 years risk of CVD calculated with Copenhagen risk score, WC = waist circumference, BP = blood pressure, RHR = resting heart rate, HDL = high density lipoprotein, non-HDL = total cholesterol-HDL, HbA1c = glycosylated haemoglobin, FEV1 = forced expiratory volume, VO**_**2max**_ **= maximal oxygen uptake, sedentary = hours of physical activity during leisure time spending ≤1.5 metabolic equivalents, MVPA = hours of moderate or vigorous activity**

	Variables	Expected difference, mean	Expected standard deviation	α	Power %
Primary outcome	10 years risk of CVD (%)	2.5	5.9	0.0166	0.90
Secondary outcomes	WC (cm)	5	14	0.0166	0.75
	BP (mm Hg)	5	12	0.0166	0.88
	RHR (per minutes)	10	20	0.0166	0.97
	HDL (mmol/l)	0.2	0.4	0.0166	0.97
	Non-HDL (mmol/l)	0.45	1.1	0.0166	0.87
	HbA1c (mmol/mol)	0.5	1.1	0.0166	0.94
	FEV1 (L)	-0.36	0.92	00166	0.84
	VO_2max_	3.5	9	0.0166	0.73
	Sedentary (minutes/day)	60	140	0.0166	0.90
	MVPA (Minutes/day)	20	40	0.0166	0.97

### Data analysis

Analysis of data will be based on the intention-to-treat principle. I.e., all patients randomized will be included in the analysis regardless of adherence to the allocated intervention. The primary outcome and other continuous outcomes will be analysed using a repeated measurement, likelihood-based, mixed-effects model with an unstructured covariance matrix. This analysis will include measurement at baseline and 12 months for the primary outcome, and all measurements (baseline, 12 months, and 24 months post-intervention) for the follow-up results, and is an appropriate approach to handling missing data. Dichotomous outcomes will be analysed using logistic regression. In case more than 5% of data is missing at follow up we will use multiple imputation to handle missing data. The imputations will be based on a linear regression model with 100 imputations and 20 iterations. The pooled analysis will subsequently be used for our analysis.

All statistical analysis will be conducted in SPSS. All tests will be two-tailed and unless otherwise mentioned the alpha level will be set at 0.01666.

### Approval

Approval from the Danish Ethical Committee: H-4-2012-051.

Approval from the Danish Data Protection Agency referral number: 01689 RHP-2012-007.

## Discussion

### Legitimacy of the study

Based on the growing mortality gap between schizophrenic patients and people without schizophrenia, there is an urgent need to improve the physical health in patients with schizophrenia, allowing them to benefit from the decline in cardiovascular disease that has been seen in the general population in developed countries. A recent Cochrane systematic review concluded that lifestyle counselling is ineffective to prevent cardiovascular disease in the general population, but recommends further research in subgroups with high risk of cardiovascular disease, as they find a modest effect on patients with diabetes or hypertension [66]. As the mortality from cardiovascular disease is twice as high in patients with schizophrenia compared to the general population, we find that the former comprises such a subgroup. Furthermore, we selected patients with increased waist circumference, due to the correlations between central obesity and metabolic disturbances [67]. Daumit et al. confirmed that weight loss is possible in this subgroup, by offering group exercise on a regular basis (three times a week) and free, healthy meals. However, this is a costly intervention demanding a reorganization of the outpatient care. With CHANGE we have developed an alternative intervention, hoping that an individualized approach integrated in the local area can be effective and sustainable, as well as reaching out for those with the most severe psychiatric and medical disabilities that might not be ready to attain regular group exercise.

### Statistical considerations

In line with current recommendations, our approach to handling missing data has been described in the study protocol [4]. Several methods have been used, including complete analysis, which excludes participants with missing outcomes or simple imputation where missing values are substituted by ‘last observation carried forward’ or mean of the sample. These methods assume that variables are missing completely at random, which is usually not the case [68], and underestimate the precision (standard error and confidence interval) [69]. Data are missing at random, given all we have observed about a person, the risk of missing a specific observation is independent of the actual value of that observation. Following this assumption, attempts can be made to substitute missing values by using multiple imputation, where a prediction model is used, and therefore accounts for the uncertainty surrounding missing data values. As this assumption of missing at are random is impossible to verify, multiple imputation will be accompanied by a sensitivity analysis, as recommended by the CONSORT guidelines [70]. In our trial, this is especially crucial, as one might speculate that participants lost to follow up had none or even harmful effects of the lifestyle intervention, which could be weight gain as a result of attempts to stop smoking.

The problem of multiplicity arises in this trial due to multiple interventions, multiple outcomes, and multiple measurements (follow-up at both 12 and 24 months after randomization), increasing the risk of type 1 error (falsely rejecting the 0-hypothesis). To account for this, analysis of primary and secondary outcomes will use a Bonferroni-corrected alpha (0.05/3), hypothesising that the lifestyle intervention will be superior to the care coordination that will be superior to the treatment a usual. This approach might be too conservative, due to a high probability of correlation between the outcomes [65]. We therefore decided to calculate unadjusted p-values, but interpret the results in accordance with values described below:

- *P* ≥0.05: The trial results could not demonstrate an effect of the experimental intervention on the secondary outcome.
- 0.01 < *P* <0.05: The trial results indicate that there may be a positive effect of the experimental intervention on the secondary outcome. However, the indication is not strong.
- 0.001 < *P* <0.01: The trial results indicate that there may be a positive effect of the experimental intervention on the secondary outcome.
- *P* <0.001: The trial results strongly indicate that there may be a positive effect of the experimental intervention on the secondary outcome.

### Outcomes

It is obvious that fatal and non-fatal cardiovascular outcomes would be the optimal outcome for interventions aiming to reduce mortality from cardiovascular disease. Facing limited time and resources though, we chose to focus on cardiovascular risk, and thus searched for the most suitable risk score model, estimating 10-years risk. The Copenhagen risk score is the best suitable in a Danish population, and has incorporated data from randomized clinical trials, thus making it the best model to estimate changes in risk [71]. Furthermore, the Copenhagen risk score can be used to estimate risk in patients with diabetes and patients with a history of cardiovascular disease. As was done in the population based study Inter99 [38], we extrapolated the age at 60 years, to reach a substantial level of risk, as no young persons have a high risk in spite of unhealthy lifestyle habits and values highly above the recommended. Additionally, by choosing a composite outcome, we reduce the risk of multiplicity, without adjusting the alpha-level.

A priori, we defined cardiorespiratory fitness as an exploratory outcome, due to insecurity about the patients’ ability and acceptance of the ‘watt max test’. After completing data collection, it was redefined to key secondary outcome. In a young high-risk population and in patients with schizophrenia, traditional risk equations tend to underestimate the risk, while cardiorespiratory fitness has consistently been shown to correlate closely to cardiovascular as well as all-cause mortality [72]. A major modifiable risk factor in the Copenhagen risk score is weight. However, recent research has questioned relevance of weight as outcome in lifestyle studies, as most patients regain weight soon after a terminated intervention, and solely focusing on weight reduction might have unhealthy implications. Our sample has a low mean age and very low cardiorespiratory fitness, and it might be just as clinically relevant for these patients to improve cardiorespiratory fitness than to lowering traditional risk factors for cardiovascular disease.

### Strengths and limitations

The CHANGE trial has several strengths. First, the design has central randomization, blinded outcome assessments, data management, data analysis, and independent funding [73-79]. Second, we planned our sample size to avoid substantial type 2 errors. Third, we use a manual-based, well described, and evidence-based theoretical framework. Fourth, the approach has a high intensity intervention, offering an assertive approach with at least weekly personal contact. Fifth, we have a multifaceted method, allowing the staff to work on all the known risk factors. Sixth, our composite outcome integrates the results even though they might be heterogeneous. Seventh, by comparing care-coordination with the lifestyle coaching, we will be able to differentiate between the effect of sufficient monitoring and treatment of somatic comorbidity and the effect of lifestyle changes, so a significant difference between the two intervention groups will point at an add-on effect of lifestyle coaching. Eighth, all contacts, and the focus of the contact, with patients are registered. Ninth, the intervention is developed to be sustainable, using low-budget possibilities in the neighbourhood to enable the patients to create long lasting changes. Ninth, we will be able to follow patients through Danish publish register to assess any long-term effects [80].

There are also limitations. Regarding some of the secondary outcomes, we will not have power to detect a clinically relevant difference, for example smoking cessation, why this important outcome has been categorized as an exploratory outcome. The thorough examination at baseline might initialize some lifestyle changes in patients randomized to the control group. The external validity is directed by the selection of patients with abdominal obesity; hence our results will only be valid for this group of patients. Moreover, an unavoidably limitation is also the selection bias created by a heightened motivation to change lifestyle habits, just by accepting participation in the CHANGE trial. Choosing a surrogate outcome like the Copenhagen risk score is a limitation due to the risk scores possible inaccuracy in predicting actual morbidity and mortality [81]. Furthermore, even though an individualized approach is necessary in order to implement lifestyle changes in daily life, it makes the trial vulnerable regarding its external validity, as not all patients will have the same interventions.

## Conclusion

This paper describes the study protocol for a randomized clinical trial to investigate the effectiveness of a tailored, multifaceted health promotion intervention versus care coordination versus treatment as usual in patients with schizophrenia in outpatient care. The primary outcome is the risk of cardiovascular disease assessed at 12 months. Secondary outcomes are physical health parameters, health related behaviours, and psychometric measures.

The lifestyle coaching is developed to adapt to real life, exploiting the possibilities of individual patients to create long lasting lifestyle changes. There is limited evidence to support the role of lifestyle interventions and care-coordination in improving weight loss and reducing metabolic risk in schizophrenia. Several smaller studies have evaluated the effect of either physical activity or diet or smoking cessation programs. However, larger sample sizes and longer follow-up time are needed.

CHANGE will increase the evidence regarding physical health in this vulnerable population, and enable clinicians to provide treatment that will reduce the mortality gap.

## Additional files

Additional file 1:
**Care coordinator manual.**

Additional file 2:
**Diet manual.**

Additional file 3:
**Physical activity manual.**
