# Supplementary material for: Protocol for CHANGE: a randomized clinical trial assessing lifestyle coaching plus care coordination versus care coordination alone versus treatment as usual to reduce risks of cardiovascular disease in adults with schizophrenia and abdominal obesity
Source: BMC Psychiatry. 2015 May 23;15:119. doi: 10.1186/s12888-015-0465-2 (PMC4460642; doi:10.1186/s12888-015-0465-2)
Supplement: Additional file 1: — Care coordinator manual. [file 12888_2015_465_MOESM1_ESM.pdf]

Oversigt:

- Formålet og Care-koordinator funktionen
- Brug af manualen
- Beslutningstræet
- Egensomsorg
- Hypertension
- Hyperlipidæmi
- Diabetes
- KOL

Formålet med care-koordinationen er at sikre, at patienten undersøges og evt. behandles for somatisk sygdom. Det er vigtigt, at der er opmærksomhed på indikatorer på øget risiko for hjertekarsygdom, diabetes, KOL og andre livsstilsrelaterede sygdomme. Care-koordinatoren skal koordinere de komplekse behandlingsforløb og der igennem øge den samlede indsats for patienten.

Care-koordinatoren:

- Funktionen er opsøgende og støttende i forhold til, at patienten kan have svært ved selv at tage vare på sit almene velbefindende og reagere på evt. somatiske symptomer.
- Skal være opmærksom på at graduerer graden af støtte i forhold til den enkelte patients behov og evne til at yde egenomsorg.
- Tjekker om alle relevante prøver er taget (er angivet i protokollen).
- Tjekker om værdierne er normale.
- Ringer til alle patienter for at informere om, at han/hun er kommet i CK-gruppen og aftale tid for 1.møde.
- Holder med alle patienter et indledende møde, hvor testresultaterne og CK-funktionen gennemgås samt patientens forventninger afklares. Her spørges også ind til pt's hverdag og fysiske tilstand.
- Aftaler som udgangspunkt med patienter med normale værdier, at vi ringer til dem hver 2.-3. md for at høre, hvordan det går med helbredet. Ved opkald spørges ind til helbredet generelt og ved gener, spørges mere direkte ind til det konkrete problem og der vurderes her ud fra, om der er behov for et møde eller om patienten skal opfordres til at søg e.l.
- Sikre sig at patienter med normale værdier ved, at de altid må kontakte CK, hvis der dukker somatiske problemstillinger op, eller de har behov for at få gentaget noget fra 1.møde.
- Tjekker patienter med normale værdier's journal 1 gang om md for at opfange, om der er kommet nye helbredsoplysninger, som patienten ikke har haft overskud til at formidle videre til CK. Hvis dette er tilfældet kontakter CK patienten.
- Tjekker op på at patienter med normale værdier kontrolleres igen efter et tidsforløb, der er relevant i forhold til den enkeltes risikofaktorer. Patienter der får antipsykotisk medicin bør somatisk udredes min 1 x årligt.
- Har hos patienter med afvigende testresultater det koordinerende ansvar for at den somatiske behandlingsplanen følges og at sikre at patienten får den støtte der er behov for. Det praktiske arbejde kan udføres af care-koordinatoren eller uddelegeres til bostøtter eller andre nøglepersoner med god kontakt til patienten. Rent praktisk kan nogle patienter måske klare det meste selv, mens andre kan have brug for hjælp til at bestille tid hos lægen og/eller komme derhen og måske til at formidle de relevante symptomer videre til lægen.

- Følger flowet i en evt. udredningsplan og reagerer få dage efter prøvesvar, lægebesøg mv. Det aftales individuelt om det er patienten, som kontakter CK efter lægebesøg mv eller om CK skal kontakte patienten, (der stiles primært efter det første) og så følges op på de initiativer, der er sat i værk.

- Tjekker om der er iværksat en somatisk behandlingsplan.

- Følger op på at patienten får hentet medicinen på apoteket og får den taget – evt kan care-koordinatoren selv eller en anden kontaktperson følger med patienten på apoteket, hvis patienten ikke selv formår at gøre det.

- Sikre at ændringer i medicin til behandling af somatisk sygdom videregives, hvis medicinen administreres af andre end patienten selv. Det kan være hjemmesygeplejerske, sundhedscenter e.a.

- Følger op på om alle kontroller huskes både af patienten og lægen.

- Følger op på om patienten følger behandlingsplanen og føler en bedring af de somatiske symptomer.

- CK må gerne på opfordring fra patienten give svar på spørgsmål om kost og motion og give gode råd, men udleverer ikke materialer, kostplaner mv, ledsager ikke til fitnesscenter, indkøb mv og coacher ikke i forhold til livsstil. De må bruge deres almene viden om kost og motion, og hvis patienten har brug for mere henvises til e.l.

- Der kan ikke fastsættes en øvre grænse for CK's kontakt og i ekstreme tilfælde må det diskuteres CK'erne imellem og med CHANGE ledelsen.

- Kan hjælpe patienten med at navigere rundt i sundhedssystemet.

- Registrerer sit arbejde i journalen og i Excel arket.

Manualen:

De **korte beskrivelser** af KOL, hyperlipidæmi, hypertension og diabetes er beregnet som let tilgængelig information om de enkelte sygdomme og symptomerne på disse. De kan bruges som en basis viden, der sammen med værdierne fra baseline undersøgelsen, kan danne grundlag for at vurdere, om patienten har behov for at komme til lægen til yderligere udredning for en eller flere af ovenstående sygdomme. Det er altid lægen som stiller diagnosen og har behandlingsansvaret.
Uddybende information kan findes i de kilder, der henvises til.

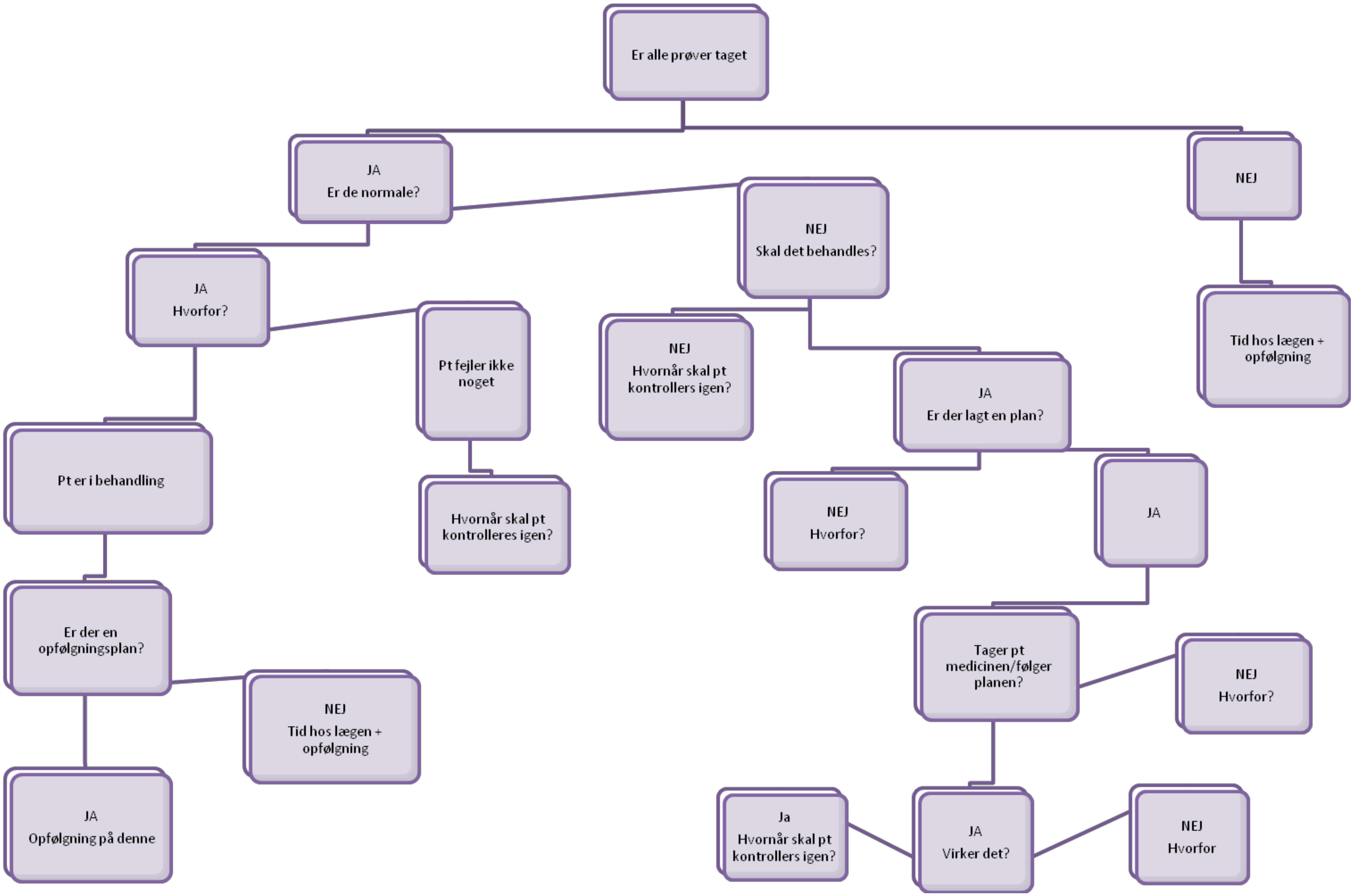
Er alle prøver taget
JA
Er de normale?
NEJ
JA
Hvorfor?
NEJ
Skal det behandles?
Tid hos lægen + opfølgning
Pt fejler ikke noget
NEJ
Hvornår skal pt kontrollers igen?
JA
Er der lagt en plan?
Pt er i behandling
Hvornår skal pt kontrolleres igen?
NEJ
Hvorfor?
JA
Er der en opfølgningsplan?
Tager pt medicinen/følger planen?
NEJ
Hvorfor?
NEJ
Tid hos lægen + opfølgning
JA
Opfølgning på denne
Ja
Hvornår skal pt kontrollers igen?
JA
Virker det?
NEJ
Hvorfor

International klassifikation af funktionsevne, funktionsnedsættelse og helbredstilstand (ICF)

WHO's internationale klassifikationssystem.

Vedligeholde egen sundhed.

"Tage vare på sig selv ved at være opmærksom på, at man har brug for og skal gøre det, der er nødvendigt til at varetagelse af egen sundhed, både ved at reagere på risikofaktorer og forebygge sygdom ved f.eks. at opsøge professionel hjælp; følge lægelige og andre råd om sundhed; undgå risikofaktorer som fysiske skader, smitsomme sygdomme, misbrug og seksuelt overførbare sygdomme."

Udgivet af Wold Health Organisation 2001, 1.udgave, 1.oplag. Dansk udgave udgivet i samarbejde med SST og Munksgaard Danmark.

Egenomsorg i.flg. Hummelvolt:

"Egenomsorg vedrører de handlinger, mennesket selv udfører for at bevare liv og sundhed. Denne evne varierer fra person til person og er forskellig i forskellige situationer og forskellige faser af livet. Når psykiske forstyrrelser og problemer melder sig, vil denne kapacitet reduceres.
Nedsat egenomsorg kan skyldes flere forhold. Den kan skyldes mangel på viden om sammenhængen mellem levevis og helbred, at man kun har lidt støtte i sit miljø, at man mangler motivation til at gøre noget ved sin livsførelse og livssituation, at ønsker til fremtiden og selvopfattelse er urealistiske, eller at der foreligger mangler på fysiske og psykiske kræfter, eventuelt reducerede intellektuelle evner."

Jan Kåre Hummelvolt: Helt – ikke stykkevist og delt.
Psykiatrisk Sygepleje 4. rev. Udgave, Ad Notam Gyldendal, Oslo 1992

Hypertension

BT > = 140/90
Ved diabetes og kronisk nyresygdom > = 130/80

Hvis disse værdier er overskredet, gøres pt opmærksom på at han/hun skal gå til e.l. obs forhøjet BT

Risikofaktorer:

- Familiær prædisponering
- Overvægt – abdominal fedme
- Stort alkoholforbrug
- Højt saltindtag
- Stress
- Type 2-diabetes
- Medicin

Hypertension giver som regel ingen symptomer undtagen ved meget høje tryk. Hvis de er til stedet kan de være,

Symptomer:

- Hovedpine
- Svimmelhed
- Funktionsdypsnø
- Anstrengelsesrelaterede brystsmerter
- Hjertebanken

Hypertension Grad 1 systolisk BT 140-159 mmHg eller
diastolisk BT 90-99 mmHg

Kontrol med 4-6 ugers interval i 3-6 mdr. Ingen medicin til at starte med, men opfordring til øget fysisk aktivitet, kostændringer og rygestop.
Obs, kortere observationstid ved diabetes og nyresygdomme.

Hypertension Grad 2 systolisk BT 160-179 mmHg eller
diastolisk BT 100-109 mmHg

Observeres kun i uger inden opstart af medicin.

Hypertension Grad 3 systolisk BT > = 180 mmHg eller
diastolisk BT > = 110 mmHg

Observeres kun i dage inden opstart af medicin.

Prøver i forbindelse med hypertension:
- Elektrolytter – natrium/kalium
- Kreatinin
- Blodlipider (HDL, LDL, triglycerid, totalkolesterol)
- Faste blodsukker og HbA1c
- Hæmoglobin
- Urinundersøgelse – om der er tegn på nyreskader
- EKG

Ved opstart af behandlingen:
- Kontroleres blodprøver og urin igen efter 3 mdr
- Hos ældre eller ved hjertekarsygdom kontrolleres kreatinin efter 14 efter opstart af ACE-hæmmer og AII-hæmmer

Efter stabilisering:
- BT kontrolleres 2-3 om året
- Årlig kontrol af kalium, urinsyre, blodlipider, blodglukose, kreatinin, urinundersøgelse og vægt

Hyperlipidæmi

Totalkolesterol	< 5 mmol/l, ved diabetes <4,5 mmol/l
LDL	< 3 mmol/l, ved diabetes 2,5 mmol/l
HDL	Skal være over 1
Triglycerider	< 2 mmol/l

Hvornår skal CK reagere på kolesteroltal

1) Ved høje værdier:

Totalkolesterol > 7,5 henvises til e.l

LDL > 5 henvises til e.l

HDL < 1, obs. på andre faktorer (triglycerid, BT etc. som oftest så vil være påvirket)

Triglycerid: Hvis kun isoleret forhøjet, (altså normale kolesteroltal), henvises først ved triglycerid >10.

Ved forhøjede triglycerid desuden obs. på livsstil og særligt alkohol (Mest relevant for Livsstilscoach)

2) Ved grænseværdier dvs. total (5,1-7,5) og LDL (3,1-7,5) henvises til e.l. hvis en eller flere af følgende er til stede:

- **BT for højt (se længere oppe)**
- **Pt er ryger**

Risikofaktorer:
- Familiær prædisponering
- Livstil (rygning, fedtholdig kost, mangel på motion, indtagelse af alkohol)
- Overvægt
- Hypertension
- Diabetes
- Nyre-lever sygdom
- Medicin: Vanddrivende, binyrebark hormon, østrogener)

Prøver/undersøgelse ved mistanke om hyperlipidæmi:
- Klinisk undersøgelse
- Blodlipider
- Blodsukker
- Kreatinin
- Urat
- BT
- Puls
- Vægt
- BMI
- Hofte/liv-mål
- Urin-stix for albumin
- EKG

Ved opstart af behandling:
- Blodprøver og urin kontrolleres månedligt indtil behandlingsmålet er opnået
- Kliniske undersøgelse efter behov

Mål for behandling:
- Patienter med hjertekarsygdom og/eller diabetes:
- Totalkolesterol <4,5 mmol/l, LDL <2,5 mmol/l

- Patienter uden ovenstående:
- Totalkolesterol <5,0 mmol/l, LDL <3,0 mmol/l

Efter stabilisering:

- 1-2 årlige undersøgelse med samme fokus som ved diagnostisering

Diabetes 2

**HbA1C**	**> = 48 mmol/L (KBH)**
	**> = 44 mmol/L (Århus)**

Ved værdier over ovenstående, rådes pt til at søge e.l. på mistanke om nyopdaget diabetes. (48 mmol(L eller derover er diagnostisk for diabetes, når 2 gentagende målinger viser dette)

Ovenstående kan ikke benyttes hos patienter, som har tilstande, hvor erythrocytterens levetid er påvirket (bl.a.alkoholisme, jern-og B12-mangel, nyreinsufficiens, hæmoglobinopatier – se mere på DSAM's nye vejl. om diabetes side 8)

I disse tilfælde eller hvis det rent praktisk ikke er muligt at få analyseret HbA1C prøven, benyttes nedenstående.

Faste BS	> = 7,0 mmol/L (6,1-6,9 mmol/L bør tages glukosetolerancetest)
Ikke faste BS	6,1-11,0 mmol/L diabetes kan foreligge – faste BS eller glukosetolerancetest tages
Ikke faste BS	> = 11,1 diabetesværdi – er der symptomer? Ellers tages faste BS eller glukosetolerancetest

Risikofaktorer:

- Familiær prædisponering
- Overvægt – abdominal fedme
- Fysisk inaktivitet
- Hypertension/hjerte-karsygdom
- Lipidforstyrrelser
- Rygning
- Alvorlig psykisk sygdom – *bør udredes 1 x årligt ved længerevarende fast beh med antipsykotika*
- Obstruktiv søvnapnøsyndrom
- PCOS
- Brug af kortikosteroider (binyrebarkhormon)

Symptomer:

- Tørst
- Vægttab
- Øget træthed
- Uoplagt
- Initiativløs
- Deprimeret
- Candida i underlivet
- Balanit (betændelse af forhuden)
- Ved meget høje værdier kan patienten være omtåget og i værste fald gå i koma

Supplerende prøver:

- HbA1c
- Blodlipider (HDL, LDL, triglycerid, totalkolesterol)
- Kreatinin
- Urinstix, glukose, nitrit, leucocytter
- Albumin/creatinin ratio (ACR) eller døgnurinopsamling
- EKG
- BT

Ved opstart af behandling:

- Kontrol 2.-4. uge i 3 mdr

Ved stabilisering:

- HbA1c hver 3.-6. mdr
- BT hver 3.-6- mdr (behøver ikke være hos lægen)
- Vægt hver 3.-6. mdr (behøver ikke være hos lægen)
- Årskontrol med fokus på tilkomne komplikationer bl.a. risiko for diabetisk fodsår og nyrepåvirkning

Fagpersoner omkring patienten bør altid være obs. på fodsår og gerne opfordre til at gå til fodterapeut (henvisning fås hos lægen)

Øjenundersøgelse:

- På diagnosetidspunktet
- Derefter årligt

KOL

Diagnosen stilles af lægen på baggrund af ryge- og erhvervsanamnese, symptomer samt spirometrifund. (FEV1/FVC <70% efter bronkodilation, samt FEV1 i % af forventet FEV1 værdi)

Investigator (Helene/Mette, Merete/HC) skriver hvis der er afvigelser, der kræver at pt går til lægen.

Risikofaktorer:

- Rygning/passiv rygning
- Erhverv
- Hyppige lungeinfektioner i den tidlige barndom
- Astma – hvis patienten er ryger

Symptomer:

- Åndenød, som bliver værre over tid og ved anstrengelse og som er til stede hver dag
- Hoste
- Eksspektorationer
- Hyppige nedre luftvejsinfektioner
- Vægttab
- Ømhed i brystkassen
- Perifere ødemer
- Træthed, dårlig søvnkvalitet
- Koncentrationsbesvær
- Depression, angst
- Social isolation

Ved opstart af behandling:

Patienten kontrolleres efter 3-6 mdr efter individuelt skøn fra lægen.

Efter stabilisering årlig kontrol af:

- Måling af lungefunktion
- Ernæringstilstand (BMI)

- Åndenød (MRC-skala)
- Tjek af inhalationsteknik
- Vurdering af risikoen for eksacerbationer og behovet for rehabilitering
- Måling af Hb og Htc med tanke på polycytæmi (antallet af røde blodlegemer) hos patienter med hypoxæmi (Lavt iltindhold i blodet)

KOL kan ikke kureres, men med rygestop og evt medicin alt efter graden af sygdommen, kan progression mindskes.

De overordnede mål ved KOL-behandling er at:

- Forhindre fortsat sygdomsprogression
- Mindske de daglige symptomer
- Forhindre og behandle eksacerbationer
- Forhindre og behandle de systemiske komplikationer af sygdommen

Mere specifikt skal man fokusere på følgende aspekter:

- Åndenød
- Hoste og opspyt
- Rygning
- Eksacerbationer
- Fysisk aktivitet
- Ernæring
- Tilstødende sygdomme

Ved iværksættelse af medicinsk behandling bør der aftales kontrol med fokus på, om patientdefinerede mål er opnået, om patienten oplever, at den iværksatte bronkodilaterende behandling har haft effekt. Hvis ikke forsøges den seponeret, og der følges op ved næste kontrol. Patienten er således sin egen kontrol.

Kilder:

DSAm's vejledninger
Lægehåndbogen
www.praksis.dk (sundhed.dk)
Dansk Cardiologisk Selskab, NBA 2012 kap.28. følger ECS guidelines (European Society of Cardiology)
