# Supplementary material for: Protocol for CHANGE: a randomized clinical trial assessing lifestyle coaching plus care coordination versus care coordination alone versus treatment as usual to reduce risks of cardiovascular disease in adults with schizophrenia and abdominal obesity
Source: BMC Psychiatry. 2015 May 23;15:119. doi: 10.1186/s12888-015-0465-2 (PMC4460642; doi:10.1186/s12888-015-0465-2)
Supplement: Additional file 2: — Diet manual. [file 12888_2015_465_MOESM2_ESM.doc]

### Indledning

Denne mappe er en manual for Projekt Change – det gælder livet´s intervention på kost området, og formålet med manualen er, at alle medarbejderne/livsstilscoach i projektet klædes på til, at arbejde med kostområdet.

Manualen kan også benyttes af andre som skal i gang med intervention eller blot have inspiration på området.

Manualen er vigtig i vores daglige arbejde og kan bruges som et redskab af alle livsstilscoach. For at gøre den praktisk anvendelig vil der foreligge forskellige skemaer så som vægtskema, kostregistrering, døgnrytme osv.

Første del af manualen er den praksisnære del, som er bygget op omkring forandringscirklen(Prochaska og DiClemente) og fokus er på intervention og samtale med deltageren.

Anden del af manualen er en samling af relevante temaer og emner, som er vigtige i arbejdet med kost.

### Baggrund

Deltagerne i projektet har diagnosen skizofreni og en taljemål der er større end sundhedsstyrelsens anbefalinger, og de kan have forskellige livsstilssygdomme eller begyndende symptomer herpå.

Hovedparten af personer med skizofreni har vist sig at have ernæringsmæssige problemer. Det erfares, at mennesker med skizofreni ofte spiser mere energitæt kost med højere fedtindhold og lavere fiber-vitamin indhold end den generelle befolkning. (Verhaeghe, Maes et al 2011)

Vores målgruppe har en markant overdødelighed pga. sygdomme relateret til uhensigtsmæssig livsstil og lever 20 år kortere end baggrundsbefolkningen. (Andersen og Pedersen 2011)

Ligeledes er det en kendsgerning, at personer med en skizofreni diagnose oftest får antipsykotisk medicin for at dæmpe de svære symptomer samt medicin til at mindske bivirkningerne.

Vægtøgning er en kendt bivirkning til antipsykotisk medicin, grundet dens påvirkning af de appetit- og mætheds regulerende neurotransmittere. Velkendte bivirkninger er ligeledes mundtørhed og deraf øget tørst samt øget appetit, især for søde sager. Dette resulterer i mange tilfælde i overvægt og fedme. Heraf kan der opstå forstyrrelser i kroppens stofskifte som kan medføre udvikling af type 2-diabetes, forhøjet blodtryk og hjerte-kar sygdom.

Personer med en skizofreni diagnose kan være domineret af negative symptomer, hvilket bl.a. medfører inaktivitet og mindre overskud til at bevæge sig. Derved opstår problemet, at der spises mere end der forbrændes på en dag.

I projekt Change vil vi have fokus på deltagernes uhensigtsmæssige kostvaner og motiverer til en kostomlægning, for derved at forebygge overvægt og på sigt livsstilssygdomme.

### Metoder og referencer

I manualen tages der udgangspunkt i sundhedsstyrelsens anbefalinger på området, og nedenstående materialer anvendes, og er udleveret til hver livsstilscoach.

- Fødevarestyrelsens 8 kostråd
- Fødevarestyrelsens tallerkenmodel
- Hjerteforeningens Indkøbsguide
- Sundhedsstyrelsens – Små skridt til vægttab – der holder
- Sundhedsstyrelsens – 10 veje til vægttab
- Sundhedsstyrelsens – Du er måske for sød
- Fuldkornsmærket
- Nøglehulsmærket

Materialet giver gode værktøjer og inspiration til hvordan der kan arbejdes med emnerne og ændring af kostvaner ud fra små overkommelige skridt.

I kostinterventionen inddrages forskellige teoretiske referencer og metoder, og her nævnes kort 3 vigtige, som er gennemgående i hele manualen.

- Forandringscirklen(Prochaska og DiClemente), anvendes til at se hvor deltageren står i forhold til at gå ind i en forandringsproces, og derved hjælpe dem i gennem de nødvendige faser. Forandringscirklen anvendes i manualen som struktur i forhold til den første del, som har fokus på det praksisnære forløb med deltageren.
- Den motiverende samtale, af Miller og Rollnick anvendes for, at understøtte deltageren til at udvinde og udnytte sin egen motivation for ændringer i livsstil. Metoden omfatter redskaber til understøttelse af vedlige­holdelse af adfærdsændringer og håndtering af tilbagefald. Desuden støtte deltagernes engagement og personlige ønske om forandring. (Miller og Rollnick, 2004 og Silver, 2002)
- Kognitive metoder, som inddrages for, at afdække deltagernes tanker, følelser, adfærd og kropslige fornemmelser i relation til kost. Fokus er på identifikation af automatisk negative tanker og tankemæssig omstrukturering. (Rosenberg 2005,Greenberger og Padesky, 2008)

**Førovervejelsesfasen:**

I "før-overvejelsesstadiet" overvejer deltageren ikke at ændre kostvaner. I denne fase er deltageren ikke begyndt at overveje, at det er et problem med for meget mad, slik og sukkersødet drikke.

Dermed er der intet ønske om at ændre på noget.

De negative sundhedsmæssige konsekvenser ved overvægten bortforklares eller benægtes.

De ulemper, der er forbundet med overvægten, bliver nedtonet. I stedet fremhæves måske de fordele, der er forbundet med spisningen af de usunde ting: *"jeg kan lide smagen" eller ”det er hyggeligt”.*

I dette stadie er det vigtigt at lytte aktivt efter forandringsudsagn.

**Hovedopgave:**

- Målet er at få deltageren til at reflektere over sin situation og forhold til kost
- Afdække nuværende og tidligere erfaringer og vaner i forhold til kost og ernæring

**Strategi:**

- Vær ikke konfronterende eller argumenterende, spørg hellere:

*Kan du ikke fortælle mig lidt mere om de fordele der er ved at spise eller drikke det du gør?*

*Jeg er nysgerrig og vil så gerne vide mere i forhold til dit forhold til mad.*

*Hvordan det er nu og hvordan det har været tidligere i dit liv?*

*Hvad tænker du om mad og drikke?*

*Nogle forbinder mad med hygge og andre kan tænke om mad, at mad blot skal bruges til at overleve?*

*Har du altid haft sådan et forhold til mad og drikke, eller har det været anderledes tidligere i dit liv? Hvorfor var det anderledes der?*

*Har du lyst til, at jeg fortæller dig lidt om de konsekvenser der er ved at være overvægtig?*

*Har du lyst til at jeg fortæller dig lidt om hvad der sker i kroppen når du spiser for meget eller usundt samt hvorfor man kan få livsstilssygdomme af overvægt?*

*Har du lyst til jeg fortæller dig lidt om hvorfor du kan føle sig afhængig af søde sager?*

- Spørg ind til deltagerens dagligdags rutiner og vaner. Åbne og nysgerrige spørgsmål uden at dømme. Brug eventuelt en hverdaglivsbeskrivelse.
- Kom gerne godt omkring fordelene ved de ”uhensigtsmæssige” kostvaner, det kan nogle gange være med til at deltageren fremkommer med forandringsudsagn.

*Hvad spiser du så typisk til aftensmad?*

*Hvordan kan det være du vælger pizza?*

*Hvad er fordelene ved det?*

### Arbejdsark: ”Kostregistrering”

### Arbejdsark: ”Personlig kostplan”

### Arbejdsark: ”Kostanamnese skema”

**Arbejdsark: ”Fordele/ulempe”**

**Overvejelsesfasen:**

Deltageren anerkender, at der er et problem og tænker på forandring, men er ikke kommet længere og gør ikke noget for at komme videre.

I overvejelsesfasen er deltageren opmærksom på sin adfærd, og begynder at overveje fordele ved at ændre denne.

Overvejelsesfasen er vigtig i forhold til udvikling af deltagerens motivation for ændring.

For at afdække motivation og parathed kan der spørges ind:

- Vigtighed i forhold til ændring
- Tro på egne evner til at mestre ændring
- Parathed

Skema kan anvendes og deltagerens kan give sig selv tal på skala fra 1-10.

Opmærksomhed på forandringsudsagn.

Livsstilscoachen er i denne fase fortsat opmærksom på fordele der er ved de ”uhensigtsmæssige” kostvaner og anerkender disse vaners betydning.

Deltageren støttes i at forstå hvordan kostvaner er opstået og hvilken positiv effekt vanerne har haft, selvom de har været uhensigtsmæssige.

Der kan i denne fase også fokuseres på værdier og diskrepans mellem værdier og aktuel adfærd.

**Hovedopgave:**

- Fokus på fordele ved de ”uhensigtsmæssige” kostvaner og støtte deltager i at forstå vanernes nyttige funktion.
- Fokus på forandringsudsagn og undersøgelse af motivation for at ændre adfærd. Passe på ikke at opstarte ændring, hvis motivation er meget lav.
- Undersøgelse af deltagerens ambivalens i forhold til at ændre på kosten.
- Fremhæv deltagerens værdier og diskrepans

**Strategi:**

- Livsstilscoach er nysgerrig og undersøgende og kan i denne fase stille spørgsmål som kan understøtte hovedopgave i denne fase:

*Hvad betyder mad for dig?*

*Hvad betyder sundhed for dig?*

*Hvad er velvære for dig?*

*Hvilke fordele er der ved ikke at ændre vaner? Er der nogle ulemper ved at ændre vaner?*

*Hvilke fordele er der ved at ændre vaner?*

*Hvad forbinder du med sund mad?*

*Hvordan ser du selv på dine kostvaner?*

*Hvilke forestillinger har du om arbejdet med kost ændring?*

*Hvad vil du gerne af med/væk fra?*

*Hvordan vil en dag være hvis du skulle undvære (et eller andet usundt)? Hvad vil være det gode ved sådan en dag?*

*Har du oplevet at kosten styrer dit liv? Hvordan?*

*Ved du noget om de helbredsmæssige fordele du opnår ved at spise anderledes/sundere?*

*Har du selv et overblik over hvad du spiser?*

*Hvis du ikke ændre kostvaner hvad tror du det kan have af betydning for dig i fremtiden?*

*Hvad tror du der kan ske hvis ikke du beslutter dig for at ændre kost vaner?*

- Få deltageren til at reflektere over diskrepansen mellem ønsker/værdier og aktuelle adfærd.
- Brug eventuelt Skala-spørgsmål omkring vigtighed, tro på evner og parathed. (se vedlagte arbejdsark). Udfra svarene kan man bruge forandringscirklen til at tale med deltageren om, hvor han/hun er i sin proces og herudfra foreslå videre plan for forløbet.
- Brug eventuelt Beslutningsbalance (se vedlagte arbejdsark)
- Ingen forsøg på overtalelse!!!

**Arbejdsark: ”Vurdering - Skala-spørgsmål”**

**Arbejdsark: ”Forandringscirklen”**

**Arbejdsark: ”Fordele/ulempe”**

**Arbejdsark ”Analyse af problemepisode – 4 kolonner”**

**Arbejdsark ”Analyse af problemepisode – 5 kolonner”**

**Arbejdsark ”Analyse af problemepisode, spørgeskema”**

**Beslutningsfasen (forberedelsen):**

I forberedelsesfasen beslutter deltageren sig for, hvad, hvordan og hvornår man ønsker ændringen skal indtræde.

Det kan være relevant at arbejde med at optage en kostanamnese for at synliggøre rytme på dagen, nattespisning, mængder, forbrug af søde sager, drikkevarer mm.

Målet skal drøftes og deltageren skal støttes i, at forklare hvad det er, som han/hun gerne vil opnå ved at ændre på kostvaner.

Fokus på processen til at nå frem til målet kan være vigtig, at have opmærksomhed på.

**Hovedopgave:**

- At deltageren finder frem til ønsker og mål for ændringen af kosten, og støttes i at forudsige og forberede sig på barrierer og forhindringer på vejen mod målet.
- At udarbejde målsætning og handleplan.

**Strategi:**

- At stille spørgsmål kan være med til, at få deltageren til at reflektere over, og sætte ord på, hvad der kan få ham/hende til at ændre på kostvaner, selv når motivationen svigter.

*Hvad ønsker du at få ud af kostændringen?*

*Hvad tænker du er realistisk at ændre for dig?*

*Hvad tænker du kan minimeres eller gøres anderledes for at komme et skridt videre?*

*Har du gjort dig tanker om hvordan du ønsker at gribe ændringen an?*

*Hvornår på dagen spiser du usundt?*

*Har du før prøvet at undlade de usunde ting? Hvad følte du?*

*I hvilke sammenhæng spiser du uhensigtsmæssigt?(tv, computer, osv.)*

*Har du en fornemmelse af om du spiser mere når du er stresset, ked af det eller vred?*

*Spiser du med andre eller alene?*

*Hvad kunne du sætte i stedet for?*

*Hvilken støtte har du behov for?*

*Hvem kan støtte dig?*

- Hvad skal målet være på kort og lang sigt?
- Hvor sikker er du på at kunne gennemføre planen?

**Arbejdsark: ”Vurdering - Skala-spørgsmål”**

**Arbejdsark: ”Samtale om risikosituationer og fristelser - ”Når jeg… vil jeg i stedet…”**

### Arbejdsark: ”Sunde snack-mellemmåltider – alternativer til det søde”

**Arbejdsark ”Typiske saboterende tanker”**

**Arbejdark: ”Grunde til, at jeg vil tabe mig”**

**Arbejdsark: ”Tænk dig slank skema”**

**Arbejdsark: ”Belønningsaftale”**

**Arbejdsark: ”Netværkets betydning” og ”netværkscirklen”**

**Arbejdsark: ”Vægtregistreringsskema”**

**Arbejdsark: ”Målbeskrivelse”**

**Arbejdsark: ”Vejledende kostplan 9000 kJ”**

**Handlingsfasen:**

I denne fase fortager deltageren de konkrete ændringer og er i gang med at iværksætte handleplanen.

Vær parat med gode råd om midler til at nå målet, men lad deltageren selv afgøre om rådene kan bruges til noget. Lad hellere deltageren selv komme med forslag.

Anerkend og ros deltageren konkret for det han/hun har gjort godt. Snak om løsninger på de problemer der eventuelt kan opstå.

**Hovedopgave:**

- Hjælp deltageren med at nå frem til den/de mest egnede løsning(er) på opståede vanskeligheder.
- Styrk deltagerens selvtillid og ressourcer.

**Strategi:**

- Få deltageren til at identificere vanskelighederne:

*”Hvad har været vanskeligt ved at ændre på kostvaner?”*

*”Hvordan lykkedes det dig at komme videre?”*

- Anerkend og ros.
- Fokus på ressourcer og positive plan fremadrettet.

*Hvad nyder du allermest ved de ting du spiser?*

*Hvor længe tror du at du kan holde ud ikke at spise usundt?*

**Arbejdsark: Derfor vægttab - ”Hvad vil jeg gerne væk fra og hvor vil jeg gerne hen”**

### Arbejdsark: ”Sunde snack-mellemmåltider – alternativer til de søde”

**Arbejdsark: ”Blodsukker”**

**Arbejdsark: ”Mad og svingninger i blodsukker”**

**Arbejdsark: ”Dagens måltider”**

**Arbejdsark: ”Gode råd til dig der er i gang med et vægttab”**

**Arbejdsark: ”Belønningsaftale”**

**Vedligeholdelsesfasen**

Det er svært at ændre indgroede vaner og være vedholdende. Hjælp deltageren med at identificere risici for tilbagefald og strategier for forebyggelse af tilbagefald.

**Hovedopgave:**

- Fokus, at forebygge tilbagefald, dvs. blive ved med at holde fast i kostændringer.
- At kunne fortsætte ændring af adfærd til trods for tilbagefald, som er normale.
- Støtte deltageren og styrke ressourcer.

**Strategi:**

- Anerkend deltagerens indsats:

*”Jeg kan mærke på dig, at du er meget opsat på, at det skal lykkes denne gang”*

- Ros deltageren for det denne gør og undgå at fortælle at det kunne være bedre:

*”Jeg synes det er rigtig flot/sejt, at du holder fast !”*

- Få deltageren til at gentage hvorfor denne besluttede sig for at ændre på kostvanerne:

*”Hvorfor var det nu, du besluttede dig for at begynde at drikke mindre cola?”*

- Få deltageren til at beskrive sin indsats og sine vanskeligheder:

*”Hvad oplever du er det sværeste ved at...?”*

- Giv deltageren mulighed for at fortælle om sine succeser:

*”Hvad er du selv mest stolt af?”*

- Få deltageren til at identificere risiko for tilbagefald:

*”Hvad frygter du mest?”*

”Hvad vil du gøre, hvis du en dag føler at du ligeså godt kan opgive?”

Få deltageren til at tænke situationen igennem og gør opmærksom på at du måske netop i den situation kan støtte deltageren og at denne derfor skal komme selvom det er ved at gå skævt.

**Arbejdsark: Gør brug af relevante ark og skemaer, som er anvendt i tidligere faser.**

## Tilbagefaldsfasen:

De fleste der forsøger at ændre vaner vil opleve tilbagefald i perioder og tilbagefald skal ikke defineres som ”nederlag”, men som episoder man kan lære noget af.

Det er vigtigt at deltageren kommer videre og ikke låses fast i denne fase.

Anerkend det deltageren har gjort eller forsøgt at gøre indtil nu og målet skal eventuelt reformuleres.

**Hovedopgave:**

- Hjælpe deltageren videre i forandringscirklen.
- At lære af tilbagefaldet.

**Strategi:**

- Fokuser på deltagerens erfaringer:

*”Hvad var det der skete?”*

*”Hvad er det, der gør det så svært?”*

- Analyser årsagerne til tilbagefaldet og bliv opmærksom på nye strategier:

*”Hvad gik der galt?”*

*”Når du ser tilbage på forløbet, er der så noget du ville have gjort anderledes?”*

*”Hvad skal der til for at undgå et nyt tilbagefald?”*

*”Hvad skal der til for at komme igang igen?”*

- Snak med deltageren om forandringscirklen (se arbejdsark) og fortæl, at det er normalt at bevæge sig frem og tilbage i denne. Verden er ikke sort og hvid, men fuld af nuancer. Der er plads til at deltageren kan have et mål om sund kost, og i perioder godt kan have ”afstikkere”, hvor adfærden ikke er målrettet. Livet kan godt være både – og.

**Arbejdsark: ”Forandringscirklen”**

**Arbejdsark: Gør brug af andre relevante ark og skemaer, som er anvendt i tidligere faser.**

### Motivation

Motivation er ifølge Miller og Rollnick en dynamisk størrelse, der hele tiden forandrer sig under indre og ydre påvirkninger. Vi skal i Projekt Change være opmærksomme på, at vores deltagere har en skizofreni diagnose og i perioder kan føle nedsat evne til at mærke egne behov og ønsker.

Mange har måske oplevet gentagne nederlag, som kan have betydning for deres motivation for at ændre adfærd. Derfor kan de meget forståeligt have problemer med motivation og kan opleves passive og initiativløse.

Motivation og ønsket om forandring tager sit udspring i oplevelsen af en mangel, eller i et ønske om, at opnå noget man savner – et behov. Oplevelsen af et behov eller et ønske er således forudsætningen for, at man kan opleve motivation (Kristensen, Viggers og Christensen, 2011).

For at skabe udvikling skal vi i Change have for øje, at udfordringen tilrettelægges ud fra hvad den pågældende kan klare, dvs. at kravene skal ligge i næste udviklingszone. Hvis kravene er for store, vil deltageren opgive eller føle fiasko. Hvis de derimod er for små, vil der ikke ske nogen forandring/udvikling(Tjørnov et al 2003).

I forhold til deltagernes motivation på kost området er det vigtigt, at coachen fremhæver de små gevinster undervejs. Det er ofte set at folk fokuserer på den langsigtede gevinst ved kostændringen og glemmer de små ændringer. Derved kan motivationen svigte (Mikkelsen, 2000).

Motivation er ikke en konstant størrelse og kan dale undervejs, men en måde at holde motivationen oppe på, er ved at huske at belønne sig selv. Mange er rigtigt gode til at bebrejde sig selv, når de ikke overholder planen, men til gengæld dårlige til at klappe sig selv på skulderen, når det lykkes for én.

Indhold af belønning har stor betydning og skal holdes op mod det mål som man har. Hvis man har mål om vægttab, er det vigtigt, at man ikke belønner sig selv med en plade chokolade eller en kage, selvom det for mange er den mest nærliggende tanke. Tanker som: ”Nu har jeg overholdt kostplanen hele ugen, så har jeg fortjent et stykke kage!” kaldes tilladende tanker, der er med til at få én til at ”dumpe i” og bryde den aftale, man har lavet med sig selv. At belønne sig selv med noget, der dybest set er dårligt for én, er som at skyde sig selv i foden. Belønningen er ikke reel og under alle omstændigheder meget kortvarig, fordi man hurtigt fortryder, og i stedet bebrejder sig selv. Man skal derfor finde andre måder at belønne sig selv på som for eksempel at give sig lov til at sidde og slappe af med en god bog, lytte til god musik eller forkæle sig på forskellige måder (Vinding, 2010)

Livsstilscoachens opgave er, at udforske og styrke motivationen for forandring hos deltagerne. Jo stærkere motivationen er over tid og sted, jo større sandsynlighed er der for, at deltageren vil påbegynde og vedligeholde kost ændringen.

**Arbejdsark ”Belønningsaftale”**

**Ambivalens**

Ambivalens er betegnelsen for den indre konflikt som overvægtige personer oplever mellem ønsket om at fortsætte med at spise som hidtil og ønsket om at ændre kostvaner.

Der er mange grunde til at folk ikke ændrer adfærd. Det er ikke kun manglende viden, men oftest er det indgroede vaner. Vanerne er svære at omlægge da de kan have stor betydning for personens hverdag, fordi denne også skal ændre alle de normer, vaner, forestillinger som er knyttet til livsstilen og som opleves naturlige.

I Change er det vigtigt at vi kender til ambivalens, og er opmærksomme på vores egne gode hensigter i forhold til at vælge side. Vælger vi side kan vi risikere at deltageren begynder at argumentere for den anden side selvom det slet ikke er der de ønsker at bevæge sig hen.

Når folk er ambivalente reagerer de måske på en måde som virker ulogisk. Derfor er det vigtigt at vi undersøger og forstår ambivalensens dynamik ellers kan personens reaktioner virke mærkelige, uforklarlige og uforståelige. (Miller og Rollnick, 2004)

Under samtalen om kost skal vi have deltagerens adfærd for øje, som kan vise ambivalensen på forskellige måder.

Læg mærke til:

- Forbehold
- Argumenter for ikke at ville ændre på kosten
- Mistroiske udsagn
- Pessimisme
- Ønsker ikke snak om fremtiden
- Ønsker ikke at konkretisere udfordringerne
- Skifter emne eller glider af

(Lind og Jaspers, 2011)

I Projekt Change vil vi støtte deltageren til at undersøge fordele og ulemper ved begge sider af ambivalensen, og herefter støtte deltageren til at få konkretiseret hvad denne vil, hvordan og hvornår. Vi ønsker at hjælpe hen imod en løsning som for dem er acceptabel og realistisk. (Miller og Rollnick, 2004)

### Barrierer/forhindringer

Alle der skal i gang med en adfærdsændring vil i starten opleve både indre og ydre barrierer(Lind et al, 2009). Det blokerer ofte for, at man kan gå i gang og især holde fast. Barriere der kan være til stede:

- Besvær, eks lave mad selv i stedet for at købe mad
- Tid, det tager for lang tid at sætte sig ind i hvordan man skal gøre det anderledes
- Vaner, eks plejer at drikke sodavand til maden
- Holdninger, sund mad er dyrt og smager heller ikke så godt
- Manglende støtte fra netværk
- Manglende tillid til at man kan klare det

(Kristensen, Viggers og Christensen, 2011)

Vi vil i Change støtte deltageren i at beskrive og identificere de barrierer de måtte opleve og som kan stå i vejen for en adfærdsændring. Efterfølgende vil vi i fællesskab med deltageren finde løsninger på de eventuelle barrierer. Ofte er det nødvendigt at prioritere, hvilke barriere der arbejdes med på de forskellige tidspunkter gennem processen (Lind, Jasper og Kjær,2009) Vi skal passe på, at vi ikke kommer til at kaste os over flere barriere på en gang hvis deltageren ikke kan rumme det.

Til enhver tid vil vi lade deltageren komme med idéer til løsningsmuligheder og dette afprøves. Først når deltageren løber tør for idéer kommer vi på banen med vores råd.

**Netværk**

Mennesket er af natur et socialt væsen, der ønsker fællesskab. Derfor er netværk en vigtig ressource i en livsstilsændring som her kostændring.

Resultater af forskellige sociologiske undersøgelser viser, at der er en sammenhæng mellem socialt netværk og psykisk lidelse. Ofte skaber sygdommen delvis eller total social tilbagetrækning (Andersen og Pedersen, 2011). Et godt netværk kan fungerer som en buffer i stresssituationer og i andre svære livssituationer (Svabø, 1993). Det sociale netværk kan være med til at sætte personen i stand til at mestre vanskelige livssituationer bedre (Svabø, 1993). Et mindre ressource stærkt netværk kan virke modsat førnævnte og dermed hæmmende.

Vores opgave bliver derfor i Change at få et overblik over deltagerens sociale og fysiske netværk og i fællesskab få idéer til hvordan denne kan bruge sit netværk på en måde som gør det muligt at få støtte og opbakning til at gennemføre/vedligeholde en kostændring.

Livsstilscoachene skal under forløbet være opmærksomme på, at de netværk deltageren skaber er mulige for dem at opretholde efter endt projekt. Vi tænker det kan gøres ved at knytte bånd til allerede eksisterende aktiviteter eks, værested, motionscenter osv. i nærmiljøet.

Spørgsmål der kan stilles i arbejdet med at udforske netværket:

- Hvordan påvirker dine omgivelser din kostændring?
- Hvad skal budskabet til denne/disse personer være?
- Er der nogen der gør det svært for dig?
- Hvem tror du kan give dig denne støtte?
- Hvad skal du bede om for at få denne støtte?
- Skal du have hjælp til at bede om denne støtte?

**Arbejdsark: ”Netværkets betydning”**

**Arbejdsark: ”Netværkscirklen”**

**Kognitiv metode**

Den kognitive model går ud fra, at menneskers følelser og adfærd påvirkes af hvordan de opfatter forskellige hændelser.

”Det er ikke situationen i sig selv der bestemmer, hvad mennesker føler, men snarer den måde, hvorpå de konstruerer en situation” (Beck, 2006).

Situationen i sig selv kan derfor betragtes som neutral, men det er hvilke opfattelser eller tanker hos hver enkelt person, som er afgørende for hvordan situationer påvirker følelser, krop og adfærd. Nedenstående figur illustrerer samspillet mellem tanker, følelser, krop og adfærd i den kognitive model.


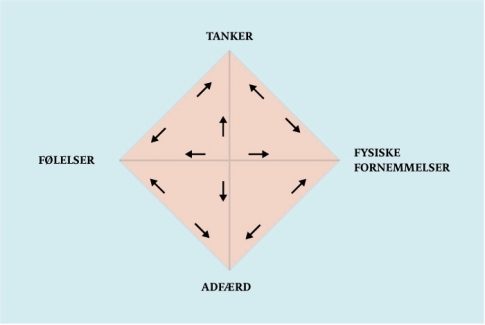


I den kognitive metode arbejder man med, at forholde sig til sine tanker, som eksempelvis kan være omstrukturering af negative tanker, som kan føre til ændret følelsesmæssige tilstand.(Greenberger, Padesky 2008)

Menneskers tolkninger af situationer er ifølge den kognitive model afhængig af antagelser om sig selv, andre og om verden. Antagelserne udvikles i barndommen og ændrer sig i løbet af livet.

I arbejdet med de kognitive metoder, er rådgiver og klient sammen om at identificere uhensigtsmæssige tanker – og adfærdsmønstre, og undersøger sammen alternativer – og mere realistiske tanker og handlemåder.

I arbejdet med kost, vil vi inddrage den kognitive metode og introducere deltagerne for sammenhængen mellem tanker, følelser, krop og adfærd, så de vil få en fornemmelse for hvordan de givne parametre påvirker hinanden.

Vi vil tage udgangspunkt i konkrete situationer og sammen med deltageren få en forståelse for problematikken samt for udløsende og vedligeholdende faktorer. Herefter vil det i mange tilfælde blive muligt for deltageren, at erstatte de uhensigtsmæssige tanker og adfærdsmønstre med mere postitive og mindre destruktive tanker.

**Vores arbejde hvor der fokuseres på kognitiv metode:**

- Udvikle en fælles forståelse af deltagernes problemer, for eksempel trøstespisning og overspisning
- Identificere negative automatiske tanker, adfærd, fysiske/fysiologiske reaktioner og følelser
- Udforske de automatiske negative tanker og deres relevans ved brug af spørgeark
- Støtte deltageren til at nuancere tankerne og danne nye mere realistiske alternative tanker, ved eventuelt at bruge kolonneskema

**Arbejdsark ”Analyse af problemepisode – 4 kolonner”**

**Arbejdsark ”Analyse af problemepisode – 5 kolonner”**

**Arbejdsark ”Analyse af problemepisode, spørgeskema”**

**Arbejdsark ”Dagskema for overspisning”**

**Arbejdsark ”Typiske saboterende tanker”**

**Arbejdark: ”Grunde til, at jeg vil tabe mig”**

**Arbejdsark: ”Tænk dig slank skema”**

**Arbejdsark ”Målbeskrivelse”**

### Appetitreguleringen

Betegnelsen appetitregulering bruges til at beskrive reguleringen af energiindtag i fht. energiforbruget. Hvornår, hvad og hvor meget vi spiser kontrolleres til dels af kognitive, sociale og kulturelle faktorer men en stor del af vores appetitregulering kontrolleres af komplekse fysiologiske systemer (Sloth, 2008).

Hjernen indeholder to forskellige centre, et sultcenter og et mæthedscenter. Det er i disse områder af hjernens hypothalamus, at appetitreguleringen bliver styret. Der findes mange forskellige fysiologiske faktorer, der i et kompliceret samspil påvirker hjernens sult- og mæthedscentre. Det er den samlede påvirkning fra alle disse signaler og den enkelte persons følsomhed overfor dem, der afgør, hvornår man bliver sulten, hvor sulten man bliver, og hvor meget man skal spise for at blive mæt igen.

Vi spiser både når kroppen har behov for mad(fysisk sult) men til tider spiser vi selvom kroppen ikke har behov for det (psykisk sult). Vi kan spise for at dæmpe negative følelser som uro, stress, ængstelse, vrede, sorg, kedsomhed, ensomhed og oplevelse af lavt selvværd. Vi opdrages med, at mad bruges som trøst, og der er næsten altid tale om søde og fede madvarer. Trøstespisning er således noget, de fleste kender til. For nogle mennesker fylder denne psykologiske funktion af mad og spisning alt for meget. Maden bliver brugt som en form for problemløsning i form af flugt fra ubehagelige tanker og følelser og psykisk smerte og erstatning for behov, som man ikke får opfyldt på anden vis for eksempel omsorg, tryghed og anerkendelse. Resultatet er ofte ikke kun overvægt, men også oplevelse af kontroltab, lavt selvværd og dårlig trivsel. For nogle ender det i en egentlig spiseforstyrrelse som Binge Eating Disorder (overspisning).

Søde og fede sager påvirker hjernens belønningssystem og dermed øger niveauet af dopamin og opioider i hjernen. Det giver psykisk ro og velvære. Der er tale om en kraftig negativ forstærkning, og det ender med at blive en meget fastgroet vane.
Derfor er det en fordel at fordele maden over dagen, så du holder energien oppe. Et måltidsmønster med 3 hovedmåltider og 2-3 mellemmåltider, holder dit blodsukker stabilt i løbet af dagen. På den måde er du mindre tilbøjelig til at falde for fristelsen til noget sødt/usundt.


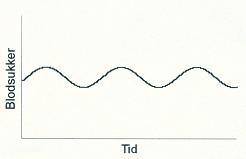

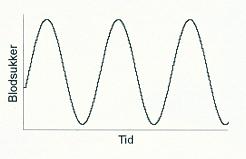


Det tager hjernen cirka 20 minutter at registrere, at man er mæt derfor kan man komme til at spise for meget hvis man spiser hurtigt.

Appetitten har stor betydning for hvor meget vi spiser. Den overordnede kostsammensætninger skal kunne forsyne hele kroppen med tilstrækkelig energi uden at hverken over- eller under-dosere vigtige næringsstoffer.

Mange af de deltagere vi kommer til at møde i Projekt Change får antipsykotisk medicin. Denne påvirker som regel appetitten således, at deltageren vil opleve øget sult eller en nedsat mæthedsfornemmelse og dermed øges deres kostindtag betydeligt (Holmsted & Hornnes, 2006)

Nogle former for antipsykotisk medicin har en sederende virkning hvilket kan føre til nedsat fysisk aktivitet og dermed nedsat kalorie forbrug.

I Projekt Change skal vi være opmærksomme på at deltagerne lærer sig hvor meget mad der er nødvendig samt opretholde en god balance, så vægtøgningen kan minimeres.Inhibitory neurotransmitters act by blocking cellular receptor sites, turning reactions off rather than turning them on.

### Kost og Motions påvirkning på vægttab

Vægttab kan enten ske ved at, man bruger mere energi end man indtager, eller at man indtager mindre energi end man bruger. Derfor er det tænkeligt, at det er en god idé udelukkende at spise mindre eller blot dyrke en masse motion. Sådan forholder det sig ikke da der er en sammenhæng mellem førnævnte faktorer.

At slanke sig uden at røre sig er en dårlig metode, eftersom mange af kroppens fysiske systemer er afhængig af en vis fysisk aktivitet.

Vægttab udelukkende ved at motionere er for de fleste ikke en oplagt mulighed. Der skal nemlig en meget stor indsats for blot at tabe ½ kilo pr uge.

Eks. For at forbrænde 3500 KJ skal man:

- - Gå i alm. tempo 3 ½ time
  - Cykle i afslappet tempo 3 ½ time
  - Boksetræning 1 ½ time

Og det skal gøres flere gange om ugen.

Regelmæssig motion hjælper til at man lettere kan ændre madvaner og forbrændingen bliver bedre. Motion bliver en del af belønningssystemet. Den bevirker at blodsukker kurven forbedres og gør det lettere at modstå sukkersulten.

Undersøgelser viser, at det er nemmere at holde vægten hvis man både forsøger at spise rigtigt og motionere (Lemmens, et. Al 2008).

For at man taber sig skal man være i underskud af energi, dvs. spise og drikke mindre energi end man har brug for. Energiunderskuddet bestemmer hvor meget man taber sig.

Eksempel:

Indtager man 2500 KJ mindre end forbruget pr. dag vil man pr. uge ca. tabe ½ kilo – 1. kg.

Anbefalingen er at man højst taber sig ¼ til ½ kilo pr uge.

Eksempel:

Energiunderskud skal dermed være 1000- 2000 KJ pr. dag.

Ved et mindre energiunderskud er gevinsten:

- Der er større chance for at vægttabet holder
- Der primært tabes fedt
- Følelsen af mæthed er højere dagen igennem
- Humøret er bedre
- Behovet for vitaminer og mineraler dækkes

Mange der beslutter sig for kostændring og vægt tab vil ofte gerne tabe sig hurtigt.

Det bør frarådes fordi:

- man vil miste relativ meget muskelmasse
- kroppen kan ikke nå at vende sig til de nye ændringer, hverken fysisk/psykisk
- risikoen for at tabet tages på igen
- vanskeligt at få dækket behovet for vitaminer/mineraler hvis energiunderskuddet er for stort

### Rygestop og vægtøgning

I forbindelse med et rygestop er der mange som tager på i vægt. Hos rygerne er nikotinens påvirkning af stofskiftet med til at holde vægten kunstigt nede. Som ryger har man et højere stofskifte og derved en højere forbrænding. Når man holder op med at ryge falder stofskiftet og derfor er det nemt at tage på i vægt (Skjødt, 2012).

Blodsukkeret stiger ved rygning og falder generelt ved rygestop. Lavt blodsukker øger rygetrangen og det er derfor vigtigt, at eks-rygeren lærer at stabilisere sit blodsukker. Blodsukkeret er generelt lavt når man vågner derfor at det vigtigt for en der lige er holdt op med at ryge at spise morgenmad kort efter de er vågnet. Det kan mindske abstinenserne. På grund af ændringer i blodsukkeret efter et rygestop er det ikke en god idé at starte en decideret slankekur hvor man indtager færre kalorier end kroppen har brug for. Det er til gengæld godt at være fysisk aktiv og gå udenom søde sager (Lind og Jaspers, 2011).

Når systemet får nikotin opstår der en kemisk tilfredsstillelse, hvor hjernens belønningssystem bliver snydt og tror, at forbruget giver noget godt og

behageligt.

Holder man op med at ryge uden at ændre kostvaner ses der en gennemsnitlig vægtøgning på 3-6 kilo som typisk sker i løbet af de første 1-3 måneder efter et rygestop. Den væsentligste årsag til vægtstigning hos nogle eksrygere er, at de ofte kommer til at trøstespise når de stopper med at ryge. De giver derved hjernens belønningssystem noget andet end nikotin som tilfredsstillelse (kræftens bekæmpelse)

Stofskiftet og forbrændingen finder et naturligt og stabilt leje efter 3-6 måneder(Skjødt, 2012).

Vægtøgning kan udskydes mens man anvender nikotinprodukter og bupropion. Efter ophør af produkterne ses det ofte at klienterne tager på.

De giftige og ætsende stoffer i røgen påvirker nerverne i lugte- og smagssanserne. Nerverne bliver bedøvet, trækker sig tilbage og nogle dør, hvorved evnen til at lugte og smage bliver dårligere.

Efter et rygestop kan skaderne blive forbedret, og eksrygeren får fuldstændigt

sin lugte- og smagssans tilbage igen. Det oplever eksrygeren ved, at denne begynder at have en bedre lugtesans og smage flere nuancer af forskellige madvarer.

Det er vigtigt at tænke på hvad man spiser som mellemmåltid og holde øje med portionsstørrelsen.

**Kostanamnese og selvregistrering**

Kostanamnese er betegnelsen for deltagerens egen redegørelse for sin kosthistorie.

Kostanamnesen bruges af personale indenfor sundhedsvæsenet, for at kunne fastslå de konkrete problemstillinger inden for kostområdet der kan være for den enkelte deltager. Kostanamnese bruges som redskab for en samtale omkring kost, hvor der deltaljeret bliver spurgt ind til indtag, mængder og regelmæssighed for indtag af mad og drikke. Der spørges ligeledes til døgnrytme, motion, rygning da alle disse områder kan have indflydelse på hvad og hvornår der spises og drikkes.

Samtalen kan slavisk følge et kostanamneseskema, det er også muligt at udlevere skemaer til selvregistrering hvor deltageren registrerer 2 hverdage samt en dag i weekenden.

Med udgangspunkt i kostanamnesen gives optimeringsforslag til:

- levnedsmiddel- og drikkevarevalg
- sammensætning af hovedkomponenter i en ret
- portionsstørrelser
- madlavningsmetodik
- måltidsmønster

##### **Arbejdsark ”Kostanamneseskema” og evt. ”selvkostregistrering”**

### Beregning af energibehov

Beregning af basal stofskiftet:

Mand på 70 kg: (100 kJ x 70 kg = 7000 kJ) skal herefter ganges med den relevante aktivitetsfaktor

Kvinde på 60 kg: (100 kJ x 60 kg = 6000 kJ) skal herefter ganges med den relevante aktivitetsfaktor

Fx: 70 kg x 100 x 1,4 (PAL 1,4 = lav aktivitet) = 9800 kJ

kJ = kilojoule, kcal = kilokalorier

1 kJ = 0,24

1 kcal = 4,2

Aktivitetsniveau:

| 1,2 | Sengeliggende |
| --- | --- |
| 1,4 – 1,5 | Stillesiddende arbejde med mindre gangaktivitet og ingen eller begrænset fysisk aktivitet i fritiden |
| 1,6 – 1,7 | Stillesiddende arbejde med en vis gangaktivitet, men ingen eller begrænset fysisk aktivitet i fritiden |
| 1,8 – 1,9 | Hovedsageligt stående arbejde (for eksempel husholdningsarbejde, butiksarbejde) |
| 2,0 – 2,4 | Tunge kropsarbejde eller meget høj fritidsaktivitet |
| + 0,3  - Tillægges de ovenstående værdier | Betydelig grad af sport eller anden hård fysisk aktivitet i fritiden (30-60 minutter 4-5 gange om ugen) |

Ved ønske om vægttab fratrækkes 2500 kJ (det giver et vægttab på ½ til 1 kg om ugen)

**Daglig energifordeling**

Energiprocentfordelingen (E%) i kosten er et udtryk for næringsstoffernes procentvise andel af den samlede energimængde.

Den daglige energifordeling opdeles i [kulhydrat](http://www.sundhedsguiden.dk/da/temaer/alle-temaer/kost-og-ernaering/generelt-om-kost-og-ernaering/kulhydrat/), [fedt](http://www.sundhedsguiden.dk/da/temaer/alle-temaer/kost-og-ernaering/generelt-om-kost-og-ernaering/fedt/) og [protein](http://www.sundhedsguiden.dk/da/temaer/alle-temaer/kost-og-ernaering/generelt-om-kost-og-ernaering/protein/), med procentfordelingen:
[Kulhydrat](http://www.sundhedsguiden.dk/da/temaer/alle-temaer/kost-og-ernaering/generelt-om-kost-og-ernaering/kulhydrat/) = 50-60% 
[Fedt](http://www.sundhedsguiden.dk/da/temaer/alle-temaer/kost-og-ernaering/generelt-om-kost-og-ernaering/fedt/) = 20-30% 
[Protein](http://www.sundhedsguiden.dk/da/temaer/alle-temaer/kost-og-ernaering/generelt-om-kost-og-ernaering/protein/) = 15-20%

Omregnes disse tal til kJ, ud fra det daglige energiindtag på fx 11.000kJ, vil næringsstoffernes andel være:
[Kulhydrat](http://www.sundhedsguiden.dk/da/temaer/alle-temaer/kost-og-ernaering/generelt-om-kost-og-ernaering/kulhydrat/) 55% (0.55x11.000)   = 6050 kJ
[Fedt](http://www.sundhedsguiden.dk/da/temaer/alle-temaer/kost-og-ernaering/generelt-om-kost-og-ernaering/fedt/) 25%        (0.25x11.000)   = 2750 kJ
[Protein](http://www.sundhedsguiden.dk/da/temaer/alle-temaer/kost-og-ernaering/generelt-om-kost-og-ernaering/protein/) 20%    (0.20x11.000)   = 2200 kJ
Samlet kJ tal                          =11.000 kJ

**Procentfordeling af kosten fordelt ud på dagen:**

Morgenmad 20-25 procent af det samlede daglige energibehov

Frokost 25-30 procent af det samlede daglige energibehov

Aftensmad 20-25 procent af det samlede daglige energibehov

Mellemmåltider ca. 15 -30 procent af det samlede daglige energibehov

**Energi (Forbrændingsværdi)**

1 gram protein giver 17 kJ (4 kcal)

1 gram kulhydrat giver 17 kJ (4 kcal)

1 gram fedt giver 37 kJ (9 kcal)

1 gram alkohol giver 29 kJ (7 kcal)

**Hvad er Body Mass Index (BMI)**

"Body Mass Index", BMI et måleparameter som kan give en ide om din idealvægt.

Hvordan måler man BMI? Udregning af BMI

For at udregne BMI skal man tage sin vægt og dividere med sin højde i anden. BMI beregnes altså med: vægt / højde2(vægt i kilogram og højde i meter).

Eksempel på hvordan man regner BMI ud

Mål for Højde: 170 cm (1,70 m)

Mål for Vægt: 80 kg

Hvad betyder BMI-tallet:

BMI under 18,5: Du vejer for lidt.

BMI 18,5-25: Din vægt er normal.

BMI 25-30: Du er overvægtig.

BMI over 30: Du er svært overvægtig.


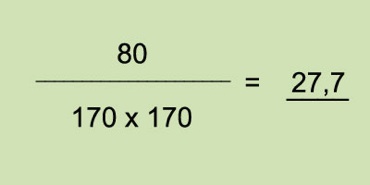


**Taljemål**

Alternativer til BMI-målet

BMI-målet er lidt upræcist, da det ikke tager højde for kropsbygning eller køn. Et alternativ til BMI er taljemål.

Taljemålet tager på den måde højde for både kropsbygning og køn i vurderingen af en persons fysiske sundhedstilstand.

Når du måler taljemål, så har højden ikke som ved beregning af BMI nogen særlig betydning.. Det der er vigtigt i vurderingen af taljemålet er det fedt du har på kroppen og i særdeleshed det fedt du har omkring din mave.

Du kan være æble eller pæreformet. Det mest usunde er bestemt at have fedtet på maven (æbleform).

**Æble eller pære**
To mennesker kan sagtens have samme BMI, men have vidt forskellige kropsform. Det er specielt æbleformen, hvor fedtet sidder rundt om maven, der medfører forøget risiko for diabetes og hjertekarsygdomme. Og målebåndet viser helt utvetydigt, hvor på kroppen man buler ud, og om man er æble eller pæreformet.

Hvis man er æbleformet vil fedtet omkring maven ophobes indvendig mellem tarmene og de indre øvrige organer. Er man derimod pæreformet, vil fedtet ophobe sig på sidebenene og bagdelen.

**Hvordan måler man sin talje**

Målebåndet føres rundt om livet. Anbring målebåndet mellem spidsen af hoftebenene og det nederste af brystkassen henover navlen. Aflæs derpå dit taljemål.


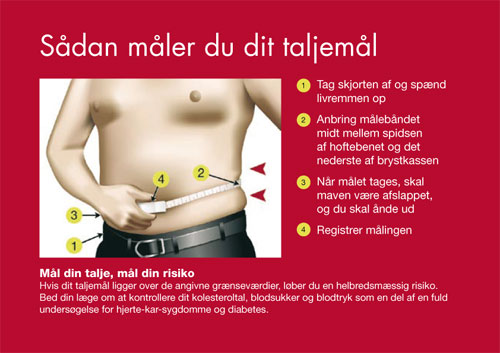


Grænser for taljemål - Europa - Kvinder og mænd

|  | Kvinder | Mænd |
| --- | --- | --- |
| Normal | Under 80cm | Under 94cm |
| Moderat overvægt | 80cm-88cm | 94cm-102cm |
| Svær overvægt | over 88cm | over 102cm |

**Kostvejledning**

##### **Samtale og undervisning individuelt og i grupper**

Det ses ofte at mennesker med en skizofreni diagnose har et større indtag af søde og fede sager. For at være realistisk er det vigtigt, at tænke plads til disse ting men med fokus på at begrænse omfanget af det.

Deltageren kan registrere deres kost over fx 3 dage og kostsamtalen kan tage udgangspunkt i dette.

Det kan ligeledes være relevant for de deltagere der er motiveret for at arbejde med kostændringer, at optage en kostanamnese for at synliggøre rytme på dagen, nattespisning, mængder, forbrug af søde sager, drikkevare mm.

**Formålet med at registrere kosten eller optage en kostanamnese er:**

- At tage udgangspunkt i deltagerens nuværende vaner
- At deltageren for en forståelse for madens betydning for sundhed og vægt.
- At deltageren definerer sin egen forståelse af sund mad
- At deltageren bliver bevidst om, hvorfor han/hun ikke spiser sundt
- At deltageren finder sine egen grunde til at spise sundt
- At deltageren kan kende forskel på fysisk og psykisk sult
- At deltageren kan finde/beskrive situationer hvor han/hun oplever psykisk sult

**Formål:**

Med udgangspunkt i kostanamnesen eller selvregistrering, kan arbejdsarkene om de enkelte måltider herunder benyttes til, at tale om de enkelte måltider. Eller der kan planlægges hjemmeopgaver ud fra arbejdsarkene ”Dagens måltider”.

**Vejledning – Individuelt**

**1. samtale**

- - Målet med samtalen er/kunne være, at finde årsagen til overvægten, introducere mulighederne i Change og undersøge, om deltageren er motiveret for at tabe sig. Afklare hvor deltageren befinder sig i forandringscirklen.

### Mål for kostvejledning individuelt som i grupper

At deltageren:

- - får viden om og forståelse for kostens betydning i forhold til regulation af blodglukose, blodlipider, blodtryk og kropsvægt, samt forebyggelse af disse
  - bliver motiveret til at spise sund kost
  - bliver bevidst om egne kostvaner
  - får relevant fødevarekendskab og foretager bevidste indkøb
  - får ideer til smags- og variationsmuligheder i tilberedningen af maden
  - lærer at omsætte anbefalingerne til praktiske handlinger/færdigheder
  - bliver bevidst om kostens betydning for egen livskvalitet
  - udvikler nysgerrighed for kostens indvirkning på blodglukose, blodlipider mm og er bevidst om kost og fysisk aktivitet som faktorer til regulation af disse
  - er bevidst om behandling og forebyggelse af kostrelateret sygdomme
  - kan opstille egne mål for kostvaner, eller samarbejder med livsstilscoachen om det i forhold til, hvad der er vigtigt og muligt at opnå
  - Deltageren bliver bevidst om sammenhæng mellem bestemte adfærdsmønstre og overvægten samt, bliver i stand til løbende at integrere mere hensigtsmæssige vaner
  - Få beskrevet et realistisk mål og sammensat en evt. kostplan med udgangspunkt i de foreliggende problemstillinger.

**Evaluering af:**

- - - kostindtag eventuelt med udgangspunkt i notaterne i kostplan og ”dagbogen”
  - - aktivitetsniveau (eventuel justering)
  - - adfærdsmodifikation eventuel inddragelse af 1 - 2 nye forhold (med udgangspunkt i tanker→følelser→adfærd)
  - - vægtudvikling (hvis deltageren ønsker det)
  - - delmål for vægtreduktion
  - - eventuelle spørgsmål, uddybning/repetition af emner der tidligere er gennemgået

(FaKD´s Rammeplan, Diætbehandling af svær overvægt hos voksne, 2009)

**Brug materiale fra SST mm.**

**Vejledning – Grupper:**

Der er ikke videnskabeligt belæg for, at gruppevejledning er mere effektiv end individuel vejledning. De undersøgelser der foreligger tyder dog på, at gruppevejledning er mindst lige så effektiv. Fordelen ved at tilbyde gruppevejledning er, at det kan give mulighed for at udveksle erfaringer, synspunkter og viden om det at tabe sig. Derudover identifikation, støtte, social netværk, inspiration og medansvar.

I gruppen ligger desuden en stor ressource i, at deltagerne ofte i modsætning til vejlederen måske tidligere har prøvet at tabe sig.

Positiv gruppedynamik og accept i en gruppe kan medvirke til, at deltagerens holdning til det at tabe sig påvirkes i positiv retning, blandt andet som følge af en eventuel øget selvaccept og forståelse hos den enkelte.

Det overordnede mål med gruppevejledningen er, at skabe et trygt og dynamisk forum, der muliggør udnyttelse af de ressourcer, der findes i gruppen og samtidig tilgodeser den enkelte deltager.

Det individuelle hensyn kan tilgodeses i et gruppeforløb ved indledende/opfølgende individuelle samtaler.

(FaKD´s Rammeplan, Diætbehandling af svær overvægt hos voksne, 2009)

**Coachens rolle i gruppeforløbet:**

- - Først i forløbet vil/kan det være nødvendigt, at coachen primært fungerer som egentlig vejleder/underviser. Hen ad vejen, i takt med gruppens indlæring og udvikling vil coachens rolle blive mere tilbagetrukket, og vil i stigende grad have en funktion af konsulentagtig karakter.

**Coachens grundlæggende opgaver er:**

- - - at sikre faglighed i forbindelse med forandring af kostvaner
  - - at være igangsætter i forbindelse med udnyttelse af de enkelte gruppedeltageres ressourcer
  - - at sikre deltagernes velbefindende i gruppen
  - - at sikre at regler for gruppens interaktion overholdes, herunder mødepræcision og mødedisciplin (fx en gruppekontrakt)
  - - at sikre at alle i gruppen kommer til orde, hvis de ønsker det.

(FaKD´s Rammeplan, Diætbehandling af svær overvægt hos voksne, 2009)

**Brug materiale fra SST mm.**

**Kost til deltagere med anden etnisk baggrund end dansk**

Mange i Danmark har rødder langt fra den vestlige kultur. Deres madkultur kan afvige fra det typiske danske valg af levnedsmidler, måltidsmønstre og måltidssammensætning.

Det er meget individuelt i hvilken grad man følger religiøse og kulturelle traditioner, det er derfor en fordel at få oplyst dette og med udgangspunkt i anbefalingerne vejlede ud fra den enkeltes behov og madkultur. (Anbefalinger for den danske institutionskost, 2011)

**Ernæringslære**


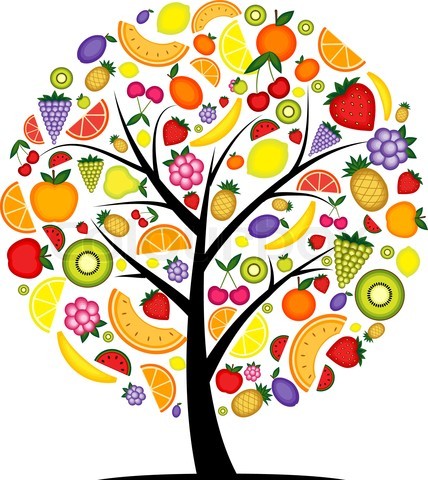


**Ernæringslære**

De nordiske lande har siden slutningen af 70érne i fællesskab udarbejdet Nordiske Næringsstofanbefalinger (NNA), som de danske anbefalinger følger.

Næringsstofanbefalingerne har til formål:

- Dække det primære behov for næringsstoffer, dvs. tilgodese individets fysiologiske behov for vækst og funktion.
- Give forudsætning for generelt godt helbred og nedsætte risikoen for kostrelaterede sygdomme.

Næringsstofanbefalingerne kan bl.a. anvendes:

- Som retningslinjer ved planlægning af kost til den enkelte samt grupper.
- Som grundlag for undervisning og oplysning om kost.

I CHANGE – det gælder livet tager vi udgangspunkt i de officielle anbefalinger og fokusere på at gøre de enkelte måltider så sunde som muligt. Det skal være konkret, praksisnært og handlingsorienteret.

**Energigivende stoffer**

Ved at spise en sund og varieret kost kan vi forebygge mange sygdomme.

Kosten består af de energigivende næringsstoffer kulhydrater, protein og fedtstoffer. Disse næringsstoffer skal indgå i vores daglige kost med en energiprocentfordeling (E%) på 50-60 E% kulhydrater, 10-20 E% protein og 25-35 E% fedtstoffer.

Vi har ligeledes brug for daglig tilførelse af vitaminer, mineraler og vand

E% betyder, at det f.eks. for fedtstoffernes vedkommende er 25-35% af den energimængde, vi totalt indtager.

**Protein**

Protein er essentielt for, at kroppen kan fungere optimalt og anvendes som en form for byggesten for muskler og væv. Da Kroppen kun har små reserver af protein, er det vigtigt, at den hele tiden tilføres tilstrækkelig protein.

Proteinrig kost egner sig både til vægttab, træning og [almindelig sund livsstil](http://www.sundhedslex.dk/livsstil.htm). Dette skyldes især, at proteiner ikke tilfører kroppen ligeså mange kalorier som f.eks. fedt.

**Proteinbehov og kost**

Menneskets proteinbehov er meget individuelt, men man kan vejledende rette sig efter følgende:

- Voksne har et proteinbehov på ca. 0,80 gram pr. kilogram kropsvægt
- Sportsfolk har et proteinbehov på ca. 1,20-1,70 gram pr. kilogram kropsvægt

Dette betyder f.eks., at regnestykket for proteinbehovet for en voksen på 80 kg ser således ud:

80 kg. x 0,80 = 64 gram protein pr. dag

For de fleste menneskers vedkommende bør protein udgøre 10-20% af det totale kalorieindtag i kosten pr. dag.

På dage eller i perioder med øget fysisk aktivitet stiger kroppens proteinbehov, hvilket betyder, at man har brug for en kost, der er mere proteinrig end normalt.

**Protein reparerer og bygger op**

Protein er en fundamental bestanddel af alle levende celler, der varetager
forskellige funktioner i menneskekroppen.

Det indgår som strukturel del af opbygning og reparation af bl.a.:

- Knogler
- Muskler
- Bindevæv
- Dannelsen af blodlegemer
- Antistoffer
- Immunsystemet

Der findes ca. 20 almindeligt forekommende aminosyrer i protein. 9 er essentielle, dvs. livsnødvendige og skal tilføres gennem kosten. 

For at kroppen kan udnytte proteinerne fra maden, må de først nedbrydes til aminosyrer, disse opbygges herefter til proteinstoffer, som menneskekroppen består af.

### 20 proteinrige fødevarer

Det er ikke kun kød og æg, der indeholder mange proteiner – der findes flere proteinrige vegetabilske produkter, end man umiddelbart skulle tro. Nedenfor findes en liste over 20 af de mest proteinrige fødevarer:

- Mælk
- Sojamælk
- Æg
- Ost
- Hytteost
- Kød
- Youghurt
- Fisk
- Fjerkræ
- Bønner
- Linser
- Tofu
- Kikærter
- Fuldkorn Bulgur
- Brød (især mørkt brød)
- Fuldkorn Pasta
- Nødder
- Kerner
- Tang

**Fedtstoffer**

Fedtstoffer – også kaldet lipider – udgør en af de essentielle næringsstoffer. Det meste af det fedt vi indtager er triglycerider (fedtsyrer), som først og fremmest fungere som energikilde.

Fedtsyrer med enkeltbinding kaldes for mættede fedtsyrer, mens fedtsyrer med en eller flere dobbeltbindinger kaldes for umættede fedtsyrer. Har fedtsyrerne en dobbeltbinding er de monoumættede (omega 9), og har de flere, er de polyumættede (omega 3 og 6).

Udover fedtsyrer indgår kolesterol i kostens fedt. Kolesterol er forstadium til binyrebark- og kønshormoner, D-vitamin og galdesyrer. Desuden er kolesterol en vigtig bestanddel af cellemembranen og er nødvendigt for transporten af triglycerider i blodet. Mennesket kan selv danne kolesterol og har således ikke behov for at få det gennem kosten.

Nogle fødevarer, som f.eks. grøntsager og frugter, indeholder ingen eller meget få fedtstoffer, hvorimod andre typer fødevarer har et højt indhold af fedtstoffer. Eksempler herpå kan være nødder, olier, smør og kød.

Navnet fedtstoffer får det muligvis til at lyde som noget, man ikke bør spise. Men fordelingen af fedtstof typerne er vigtig for en sund kost fordi:

- Fedtstoffer transporterer de fedtopløselige vitaminer A, D, E og K omkring i kroppe
- Fedtstofferne forsyner kroppen med essentielle næringsstoffer som f.eks. de fedtopløselige vitaminer og de essentielle fedtsyrer

- Essentielle fedtsyrer kan kun indtages via kosten eller via kosttilskud, og menes at have en gavnlig effekt på menneskets helbred og immunforsvar

- Fedtstoffer spiller en afgørende rolle i forbindelse med membraners struktur

- Fedtstoffer indlemmer og beskytter [kroppens indre organer](http://www.sundhedslex.dk/indre-organer.htm)

- Fedtstoffer oplagres i fedtvævet som en langvarig energi reserve. Overskydende fedt kan også ophobe sig omkring kroppens organer – især i organerne i bughulen (abdomen)

Det er nemt at indtage for mange kalorier, når man spiser fed mad eller fede madvarer. Mennesker der forsøger at få deres vægt under kontrol, bør derfor undgå fede fødevarer og fedtholdige måltider.

Alle mennesker har behov for fedtstoffer i deres kost, men nøglen til et godt helbred og en sund vægt er små mængder essentielle fedtsyrer.

### De to typer fedtstoffer

Fedtstoffer (lipider) kan opdeles i 2 hovedgrupper – mættede og umættede fedtsyrer.

#### Mættede fedtsyrer

Mættet fedtsyre er generelt fast ved stuetemperatur og stammer almindeligvis fra dyr (animalsk). Det findes i:

- spæk
- smør
- hård magarine
- ost
- sødmælk - og alle fødevarer, der indeholder disse ingredienser.

Eksempler herpå kan være:

- kager
- chokolade
- kiks
- tærter
- bagværk

Det mættede fedt er også det hvide fedtstof, man kan se på rødt kød samt under skindet på en kylling.

De præcise sammenhænge mellem mættet og umættet fedt i vores kost er endnu ikke fuldt forståede, men generelt kan man forbinde indtagelse af for meget mættet fedt med forøgede koncentrationer af kolesterol i blodet samt øget risiko for hjertesygdomme. At spise mindre mængder mættede fedtstoffer, hjælper til at minimere risikoen for hjertesygdomme.

#### Umættede fedtsyrer

Umættet fedt er almindeligvis flydende ved stuetemperatur og stammer generelt fra planter (vegetabilsk). Monoumættede fedtsyrer og flerumættede fedtsyrer tilhører begge denne gruppe.

Umættede vegetabilske olier er generelt sundere alternativer til mættet fedtstof og kan findes i:

- sesam
- solsikker
- soya
- olivenolie
- flydende margarine
- fede fisk såsom makrel, sardiner og laks

Når det er muligt, bør man vælge umættet fedt frem for mættet.

**Transfedtsyrer** - også kaldet hydrogeneret fedt – anvendes i fødevareindustrien, men opfattes mere og mere som værende usundt og der er lovgivningsmæssigt lavet retning for brugen af transfedtsyrer. Det industrielt fremstillede transfedt anvendes til bagværk, fastfood og snacks. Desuden findes det naturlige i okse og får kød samt mælkefedt.

**Anbefalede mængder**

De officielle anbefalinger er, at kosten ikke bør indeholde mere end 35% fedtstof, og at de mættede fedtsyrer ikke bør udgøre mere end 10% af de totale fedtstoffer.

For den gennemsnitlige kvinde betyder dette ca. 70 gram fedtstof totalt på en dag, og for mænd gælder ca. 95 gram.

I flere lande – herunder Danmark – viser undersøgelser, at den voksne del af befolkningen spiser den korrekte mængde fedt, men at andelen af mættet fedt er for høj (et gennemsnit på omkring 13% af de totale fedtstoffer i 2010).

For at mindske mængden af fedtstoffer i kosten kan du prøve følgende:

- Find alternativer til kager, kiks og snacks der ofte har et for højt indhold af fedtststoffer. Prøv i stedet frugt og morgenmadsprodukter (cerealer).
- Skær alt synligt fedt af kød og fjerkræ.
- Køb fedtfattige kødudskæringer samt hakket kød med en lav fedtprocent.
- Porchér (tilberedning i vand, som er lige under kogepunktet), damp, kog, grill eller bag fødevarer frem for at stege dem.
- Gå efter mælkeprodukter med et lavt fedtindhold.
- Hvis du anvender spæk, smør og margarine, så udskift disse med vegetabilsk olie og fedtfattige margarineprodukter

**Kulhydrat**

Kulhydrater er en vigtig energikilde og enkelte væv, blandt anden centralnervesystemet (hjerne og rygmarv), foretrækker glukose som energikilde.

Små mængder kulhydrat oplagres i organismen i form af glykogen i lever og muskler. Får kroppen ikke tilført tilstrækkeligt med energi, vil disse lagre være opbrugt i løbet af ½ til 1 døgn.

## Her finder du de vigtigste kulhydrater

De vigtigste kulhydrater i kosten findes i form af:

- Stivelse
- Kostfibre (groft brød, frugt og grønt)
- Sukkerarter (glukose)

Disse kulhydratformer nedbrydes i mave-tarmsystemet til glukose, og glukosen optages i blodet, hvorefter den:

- Føres til musklerne, og forbrændes til energi og varme
- Holder blodsukkeret stabilt
- Omdannes til glykogen, der lagres i lever og muskler

Under fysisk aktivitet omdannes glykogenet igen til glukose, der giver musklerne energi til at arbejde med. Glykogenet i musklerne er den bedste tilgængelige form for brændstof.

## Kulhydrater omdannes kun i ringe grad til fedt

På en normal kost kan mennesket kun i ringe grad omdanne [kulhydrat](http://www.sundhedsguiden.dk/da/temaer/alle-temaer/kost-og-ernaering/generelt-om-kost-og-ernaering/kulhydrat/) til fedt, idet de enzymer der medvirker i omdannelses processen har meget ringe aktivitet. 

En kost med meget få kulhydrater giver hurtigt et vægttab, men varer ikke længe, da det er vand der tabes. Årsagen er, at kroppen tømmer sine glykogen lagre, der binder en del vand.

## Daglige anbefalinger af kulhydrater

Kulhydrater bør udgøre 50-60% af det daglige indtag

- max 10% fra industrielt fremstillet sukker
- ca. 40-50% fra komplekse kulhydrater.

et højt indtag af komplekse kulhydrater, forbedrer den fysiske præstationsevne, mindsker risikoen for overvægt og sikrer en høj andel [kostfibre](http://www.sundhedsguiden.dk/da/temaer/alle-temaer/kost-og-ernaering/generelt-om-kost-og-ernaering/kostfibre-er-vigtige-for-din-sundhed/) i kosten.

Hvad er komplekse kulhydrater

Komplekse kulhydrater er med til at sikre et stabilt blodglukose niveau.

Komplekse kulhydrater er stivelse og fibre. De kulhydrater der tilfører kroppen vitaminer, mineraler, [kostfibre](http://www.sundhedsguiden.dk/da/temaer/alle-temaer/kost-og-ernaering/generelt-om-kost-og-ernaering/kostfibre-er-vigtige-for-din-sundhed/) og stivelse, er fødevarer såsom;

- groft brød
- rugbrød
- fiberbrød
- grønsager
- frugt
- bælgfrugteri form af både friske og tørrede (bønner, kikærter og linser)
- kartofler
- pasta
- ris(pasta og ris bør dog indtages i grovest mulige form, så som brune ris og fuldkornspasta)

## Kostfibre

Kostfibrene giver maden mere fylde og indeholder få fordøjelige kalorier. En fiberrig kost har således større volumen og giver dig en bedre mæthedsfornemmelse end en fiberfattig kost, så man føler sig mæt i længere tid. Derudover har de betydning for optagelse af næringsstofferne i mave og tarm.

Det er vigtigt altid at drikke rigeligt med vand, hvis du spiser en fiberrig kost for at undgå forstoppelse.

I gennemsnit bidrager kostfibre med 8 kJ pr. gram.

En voksen person bør spise mellem 25 og 35 g kostfibre dagligt.

### Fuldkorn

Hvorfor er fuldkorn sundt?

- - Fuldkorn er sundt, fordi alle de gavnlige mineraler, vitaminer, fedt, protein, kulhydrat og fibre er bevaret.

De gode egenskaber

- - Du har lettere ved at holde vægten, fordi fuldkorn giver en bedre fordøjelse, en øget mæthed og et stabilt blodsukker i længere tid.
  - Fuldkorn forebygger sygdomme, fordi de mange gavnlige indholdsstoffer medvirker til at mindske risikoen for type 2-diabetes, hjerte-kar-sygdomme og kræft.

Faldgruber

- - Følgende er ikke fuldkorn: Solsikkekerner, græskarkerner, sesamfrø, vilde ris og boghvede.
  - I almindeligt hvedemel bruges kun den del af kernen, der hedder frøhvide og er derfor ikke fuldkorn. Heller ikke cornflakes og couscous er fuldkornsprodukter, idet kun dele af kornet bruges.


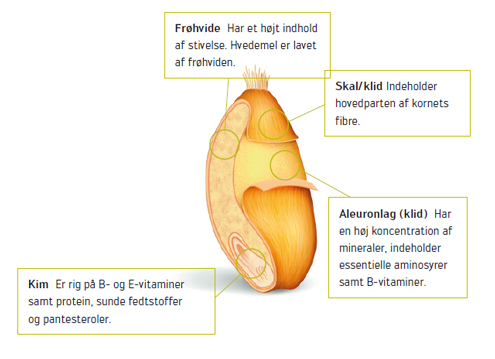


## Undgå kulhydrater fra

- hvidt brød
- sukker
- sodavand
- læskedrikke.

Et højt indtag af industrielt fremstillet sukker giver en lav næringstæthed, hvilket forklarer hvorfor højst 10% af kosten bør indeholde raffinerede sukkerarter.

### Sukker

### Sukkergruppen omfatter almindelig sukker, melis, druesukker, frugtsukker, sirup og honning.

Sukker findes i store mængder i marmelade, i sukkerholdige sodavand og andre læskedrikke samt i slik og kage/kiks.

Sukker indeholder praktisk taget ingen livsnødvendige næringsstoffer. Et højt sukkerindtag resulterer derfor i en tilsvarende nedsat indtagelse af mere lødige levnedsmidler og dermed af kostfibre og andre nødvendige næringsstoffer.

**Fakta om tørret frugt (pr. 100 g)**

Tørret frugt er ikke tilsat sukker, men indeholder store mængder naturligt frugtsukker og også vigtige næringsstoffer som kostfibre, vitaminer og mineraler. Når væsken er væk, er koncentrationen af frugtsukker i tørret frugt stort set lige så højt som i slik – næsten femseksdoblet i forhold til den friske frugt. Tørret frugt vinder sundhedsterræn i forhold til slik, fordi det er rigt på kostfibre og næringsstoffer, men på sukkerindholdet er der ingen forskel.

Friske figner indeholder 19,2 g simple kulhydrater i form af frugtsukker, men i den tørrede version kommer vi helt op på 63, 9 g.

I rå abrikoser er indholdet af simple kulhydrater i form af frugtsukker 9,4 g. Tørrede abrikoser har et sukkerindhold på 66,5 g.

Friske vindruer indeholder 14,7 g frugtsukker, men forvandlet til rosiner når vi helt op på 78,8 g.

(DTU Fødevareinstituttets hjemmeside www.foodcomp.dk)

Vitaminer

Vitaminer er livsnødvendige organiske stoffer, som kroppen ikke selv kan danne. De inddeles i fedtopløselige og vandopløselige vitaminer.

Fedtopløselige vitaminer:

- A -vitamin
- D- vitamin
- E- vitamin
- K-vitamin

Vandopløselige vitaminer:

- B-vitamin
- C-vitamin

| Vitamin: | Kilder: | Nødvendigt for: | Anbefalet dagsdosis: |
| --- | --- | --- | --- |
| [A-vitamin (retinol)](http://www.netdoktor.dk/vitaminer/vitamina.htm)   | | 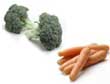 | | --- | |  | |  | | | --- | --- | --- | --- | | Kød, æg og mejeriprodukter. Desuden orange og mørkegrønne grøntsager. | Synet, vækst, immunforsvaret, tør hud, appetit og smag. Desuden for fosterets udvikling af organer. | Mænd og kvinder: 1 milligram. |
| [B1-vitamin (tiamin)](http://www.netdoktor.dk/vitaminer/vitaminb1.htm)   | | 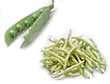 | | --- | |  | |  | | | --- | --- | --- | --- | | Kornprodukter, bælgfruger og linser. Desuden magert kød og mejeriprodukter. | Omdannelsen af kulhydrater til glukose, som er vigtigt for bl.a. hjernen, hjertet og musklers funktion. | Mænd: 1,4 mg. Kvinder: 1,1 mg. Ved graviditet og amning stiger behovet med 0,5 mg pr dag. |
| [B2-vitamin (riboflavin)](http://www.netdoktor.dk/vitaminer/vitaminb2.htm)   | | 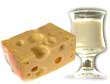 | | --- | |  | |  | | | --- | --- | --- | --- | | Mejeriprodukter, fuldkornsprodukter, kød, blad-grønt, bælgfrugter og fisk. | Vækst, hud, negle, hår, læber og tunge samt synet. | Mænd: 1,7 mg. Kvinder: 1,3 mg. Ældre kan nøjes med hhv. 1,5 og 1,2 mg. Gravide: 1,6 mg. Ammende: 1,7 mg pr. dag. |
| [B3-vitamin (Niacin)](http://www.netdoktor.dk/vitaminer/niacin.htm)   | | 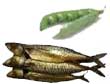 | | --- | |  | |  | | | --- | --- | --- | --- | | Kød, fisk, fjerkræ og fuldkornsprodukter. | Omsætningen af proteiner, fedt og kulhydrater til energi i kroppen. | Mænd: 16 -20 NE, og 1-2 NE mindre som ældre. Kvinder: 15 NE. Gravide: 17 NE. Ammende: 19 NE. |
| [B6-vitamin](http://www.netdoktor.dk/vitaminer/vitaminb6.htm)   | | 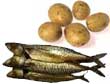 | | --- | |  | |  | | | --- | --- | --- | --- | | Magert kød, grøntsager, fuldkornsprodukter og mælk. | Omdannelse og udnyttelse af protein og fedt samt dannelse af arvemateriale. | Mænd: ca. 1,5 milligram pr. dag. Kvinder: 1,2 milligram pr. dag. |
| [B12-vitamin](http://www.netdoktor.dk/vitaminer/vitaminb12.htm)   | | 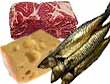 | | --- | |  | |  | | | --- | --- | --- | --- | | Magert kød og fisk samt æg og mejeriprodukter. | Omsætning af fedt og aminosyrer til protein, hvilket er vigtigt ved celledeling. Vedligeholder nerverne. | Mænd og kvinder: 2 mikrogram. Ammende: 2,6 mikrogram. |
| [C-vitamin](http://www.netdoktor.dk/vitaminer/vitaminc.htm)   | | 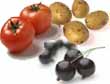 | | --- | |  | |  | | | --- | --- | --- | --- | | Citrusfrugter, bær, tomater, blomkål, kartofler og grønne blad-grøntsager. | Har betydning for kroppens immunforsvar, der blandt andet beskytter mod virus og bakterier. | Mænd og kvinder: 75 mg. Gravide: 85 mg. Ammende: 100 mg. |
| [D-vitamin](http://www.netdoktor.dk/vitaminer/vitamind.htm)   | | 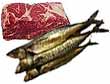 | | --- | |  | |  | | | --- | --- | --- | --- | | Æg, fede fisk og fede mælkeprodukter. | Øger optagelsen af calcium fra tarmsystemet og er nødvendigt for knoglernes og tændernes omsætning af calcium og fosfat. Sikrer balance i immunsystemet. | Mænd og kvinder: 7,5 mikrogram. Ældre, gravide og ammende: 10 mikrogram. |
| [E-vitamin](http://www.netdoktor.dk/vitaminer/vitamine.htm)   | | 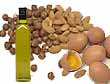 | | --- | |  | |  | | | --- | --- | --- | --- | | Fedtstoffer, kornprodukter, æg, ost og mælk. | Beskytter kroppens celler mod nedbrydning. Kan stimulere immunsystemet. | Mænd: 10 mg. Kvinnder: 8 mg. Gravide: 10 mg. Ammende: 11 mg. |
| [K-vitamin](http://www.netdoktor.dk/vitaminer/vitamink.htm)   | | 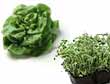 | | --- | |  | |  | | | --- | --- | --- | --- | | Mørkegrønne grøntsager som avocado, broccoli, kål, spinat og vindruer. Derudover lever, nyre, mejeriprodukter, kornprodukter, kød og frugt. | Blodets størkningsproces samt dannelsen af vigtige proteiner. | Mænd: 0,08 mg. Kvinder: 0,065 mg. |
| [Folsyre](http://www.netdoktor.dk/vitaminer/folinsyre.htm)   | | 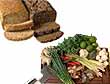 | | --- | |  | |  | | | --- | --- | --- | --- | | Bælgfrugter, grønne grønsager og lever. Desuden kornprodukter, frugt og mælkeprodukter. | Gavnligt for de røde blodlegemer og for cellernes deling. | Mænd og kvinder: 300 mg. Dog, kvinder ml. 18-30 år: 400 mg. Gravide og ammende: 500 mg. |
| [Pantotensyre](http://www.netdoktor.dk/vitaminer/pantotensyre.htm)   | | 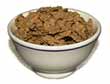 | | --- | |  | |  | | | --- | --- | --- | --- | | Kød, fuldkornsprodukter, broccoli, ærter og bønner er de bedste kilder. Derudover mælk, æg, lever og bælgfrugter. | Omdannelsen af maden til energi. Gavner desuden nervesystemet og produktionen af hormoner. | Mænd og kvinder: 4-7 milligram. |

**Mineraler**

Omkring 15 mineraler er livsnødvendige og må tilføres gennem kosten.

Mineraler her en lang række funktioner herunder:

- Byggeelementer i knogler og tænder (kalcium, magnesium og forfor)
- Regulatorer af kropsvæsker (natrium, klorid, kalium, magnesium og fosfat)
- Komponenter i mange enzymer og andre proteiner

| Mineral: | Kilder: | Nødvendigt for: | Anbefalet daglig dosis: |
| --- | --- | --- | --- |
| [Jern](http://www.netdoktor.dk/vitaminer/jern.htm)   | | 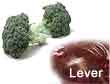 | | --- | |  | |  | | | --- | --- | --- | --- | | Kornprodukter, kød (bl.a. lever), frugt og grønt (bl.a. broccoli). | Jernet er ansvarlig for, at ilten transporteres rundt i kroppen og er derfor livsvigtigt. | Mænd og ikke menstruerende kvinder: 9 mg pr dag. Drenge: 11 mg. Piger og menstruerende kvinder: 15 mg. |
| [Zink](http://www.netdoktor.dk/vitaminer/zink.htm)   | | 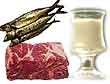 | | --- | |  | |  | | | --- | --- | --- | --- | | Kød, ost, mælk, fuldkornsprodukter. | Stofskiftets omsætning. Har stabiliserende virkning på arveanlæg og cellemembraner. Vigtig for dannelse og vækst af væv og organer samt for sårheling. | Mænd: 9 milligram. Teenagedrenge: 12 milligram. Kvinder: 7 milligram. Teenagepiger: 9 milligram. Gravide: 9 milligram. Ammende: 11 milligram. |
| [Jod](http://www.netdoktor.dk/vitaminer/jod.htm)   | | 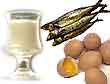 | | --- | |  | |  | | | --- | --- | --- | --- | | Stort set alle føde- og drikkevarer indeholder små mængder jod, men de dagligvarer, der indeholder mest jod, er: fisk og skaldyr samt mel æg og grøntsager | Dannelsen af hormon fra skjoldbruskkirtlen, som medvirker til regulering af kroppens stofskifte. | 150 mikrogram |
| [Fosfor](http://www.netdoktor.dk/vitaminer/fosfor.htm)   | | 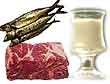 | | --- | |  | |  | | | --- | --- | --- | --- | | Mejeriprodukter, konrprodukter, kød og grønsager. | Energiomsætningen i kroppen. Byggesten for DNA og er vigtige for knogler og muskler. | Børn og unge: 700 milligram. Voksne: 600 milligram. Gravide: 700 milligram. Ammende: 900 milligram. |
| [Selen](http://www.netdoktor.dk/vitaminer/selen.htm)   | | 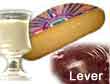 | | --- | |  | |  | | | --- | --- | --- | --- | | Kød ( især i lever og nyre), fisk, skaldyr, mælk, ost og æg, samt visse nødder, især paranødder. | Kan virke som antioxidant, der beskytter cellerne mod ødelæggelse. Også vigtig for dannelse af hormoner, som holder stofskiftet oppe. | Mænd: 50 mikrogram. Kvinder: 40 mikrogram. Gravide og ammende: 55 mikrogram pr. dag. |
| [Magnesium](http://www.netdoktor.dk/vitaminer/magnesium.htm)   | | 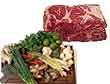 | | --- | |  | |  | | | --- | --- | --- | --- | | Kornprodukter, mælk og grønsager. Især bladgrønsager og mandler er rige på magnesium. | Stofskiftet, samt for at vi kan bruge vores muskler på normal vis, og at hjertet fungerer normalt. | Drenge og mænd: 350 mg. Piger og kvinder: 280 mg. |
| [Kobber](http://www.netdoktor.dk/vitaminer/kobber.htm)   | | 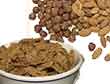 | | --- | |  | |  | | | --- | --- | --- | --- | | Indmad (specielt lever), fuldkornsprodukter, nødder og skaldyr. | Dannelse af hormorner og signalstoffer til brug i hjerne, nerver og binyrer. Beskytter mod frie radikaler, der er skadelige affaldsprodukter fra cellernes stofskifte. Godt for jernomsætning i røde blodlegemer. | Mænd og kvinder: 1,2 milligram per dag. |
| [Krom](http://www.netdoktor.dk/vitaminer/krom.htm)   | | 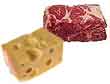 | | --- | |  | |  | | | --- | --- | --- | --- | | Kød, ost, nødder, mørk chokolade, fuldkornsprodukter, øl-gær, skaldyr mv. Det er nødvendigt at spise varieret for at få dækket behovet. | Krom er hjælpestof til insulin og fremmer sukkeroptagelsen i cellerne. Dette hjælper til at holde blodsukkeret nede. | Man kender ikke behovet for krom, men et indtag på 20-200 mikrogram per dag menes at være tilstrækkeligt. |
| [Mangan](http://www.netdoktor.dk/vitaminer/mangan.htm)   | | 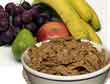 | | --- | |  | |  | | | --- | --- | --- | --- | | Fuldkornsprodukter, nødder, grøntsager og frugt. Desuden i te og kaffe. | Deltager i stofskiftets omsætning af kulhydrater og fedtstoffer ved neutralisering af frie radikaler. | Behovet for mangan er ikke kendt, men et indtag på 2,5 - 5,0 mg menes at være tilstrækkeligt til at dække alles daglige behov. |
| [Calcium](http://www.netdoktor.dk/vitaminer/calcium.htm)   | | 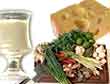 | | --- | |  | |  | | | --- | --- | --- | --- | | Mejeriprodukter som mælk og ost, samt i grove grønsager som broccoli, bønner, små fede fisk og kål. | Giver knoglevævet styrke. Derudover stor betydning for muskelsammentrækning, impuls-ledning i nerverne, størkning af blodet, blodtryk og måske vægtregulering. | Børn og unge: 900 mg. Voksne: 800 mg. Gravide og ammende: 900 mg pr. dag. |
| [Molybdæn](http://www.netdoktor.dk/vitaminer/molybdaen.htm)   | | 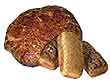 | | --- | |  | |  | | | --- | --- | --- | --- | | Findes i mælke- og kornprodukter. | Vigtig i svovl- og urinsyrestofskiftet samt kan muligvis nedsætte risikoen for huller i tænderne. | Behovet for molybdæn er ikke fuldstændigt kendt, men det menes at ligge på omkring 75 mikrogram per dag. På grund af usikkerheden er anbefalingerne dog noget højere, 75-250 mikrogram/dag,voksne. |

### Drikkevarer

Voksne skal drikke 1 ½ -2 liter væske pr. dag. Ved fysisk aktivitet øges væskebehovet med ca. ½ - 1 liter pr. times aktivitet.

Vand indtages i form af drikkevarer og i form af den faste fødes naturlige vandindhold.

Indtaget af mælk tæller med i væskeregnskabet og bør højst udgør ½ l om dagen (fedtindhold max. 0,7 pr. 100 g)

Det anbefales at begrænse indtaget af kaffe og the til 3 krus om dagen.

Vær opmærksom på koffeinholdige drikke. Disse drikke kan påvirke søvn, give uro i kroppen og blive vanedannende.

Alkohol

Alkohol indeholder energi svarende til 30 kJ pr. gram, men indeholder ingen næringsstoffer. Indtagelse af alkohol bør bølge anbefalingerne fra SST.

Grundet alkohols høje energiindhold, skal der en del motion til at forbrænde det:

Hvor meget skal jeg gå/løbe for at forbrænde*

- 1 alm. øl 1,8 km

- 1 stærk øl 2,2 km

- 1 lys øl 1,2 km

- 1 glas rødvin (15 cl) 1,6 km

- 1 snaps (4 cl) 1,3 km

- 1 dobbelt gin og tonic 2,4 km

* Værdierne er beregnet for en person på 70 kg

**Koffein**

Det er meget individeuelt hvordan vi reagerer på koffein. Koffein kan ændre kemien i hjernen. Når vi indtager koffein, er den akutte effekt en stigning i blodtrykket. Koffeinen gør også, at vi frigiver kortisol og adrenalin, to stresshormoner, der påvirker hele vores krop og speeder den op. Derudover blokerer koffeinen vores adenosin-receptorer.

Adenosin er et stof i hjernen, der produceres i løbet af dagen. Jo længere, vi har været vågne, jo mere adenosin har vi i hjernen, og når adenosinen sætter sig på vores adenosin-receptorer, oplever vi det som træthedssignaler. De fortæller os, at kroppen og hjernen har brug for søvn. Koffein blokerer adenosin-receptorerne og blokerer dermed vores træthedsfornemmelse, så kroppen og hjernen er stadig trætte, men vi kan ikke mærke det. Koffein kan derfor forstyrre vores søvn.

### Indholdet i fødemidler

- Én kop kaffe indeholder omtrent 50 til 150 mg koffein.
- I [chokolade](http://da.wikipedia.org/wiki/Chokolade) findes koffein sammen med theobromin og andre opkvikkende stoffer.
- Cola- og energidrikke er tilsat syntetisk fremstillet koffein.

## Koffeins væsentligste bivirkninger

- Øger [blodtrykket](http://da.wikipedia.org/wiki/Blodtryk)
- Øger [pulsen](http://da.wikipedia.org/wiki/Puls)
- Udvider [bronkierne](http://da.wikipedia.org/wiki/Bronkier)
- Virker opkvikkende på [centralnervesystemet](http://da.wikipedia.org/wiki/Centralnervesystemet)
- Virker [urindrivende](http://da.wikipedia.org/wiki/Urin)
- Øger [tarmens](http://da.wikipedia.org/wiki/Tarm_(anatomi)) [peristaltik](http://da.wikipedia.org/wiki/Peristaltik)

<http://da.wikipedia.org/wiki/Koffein>


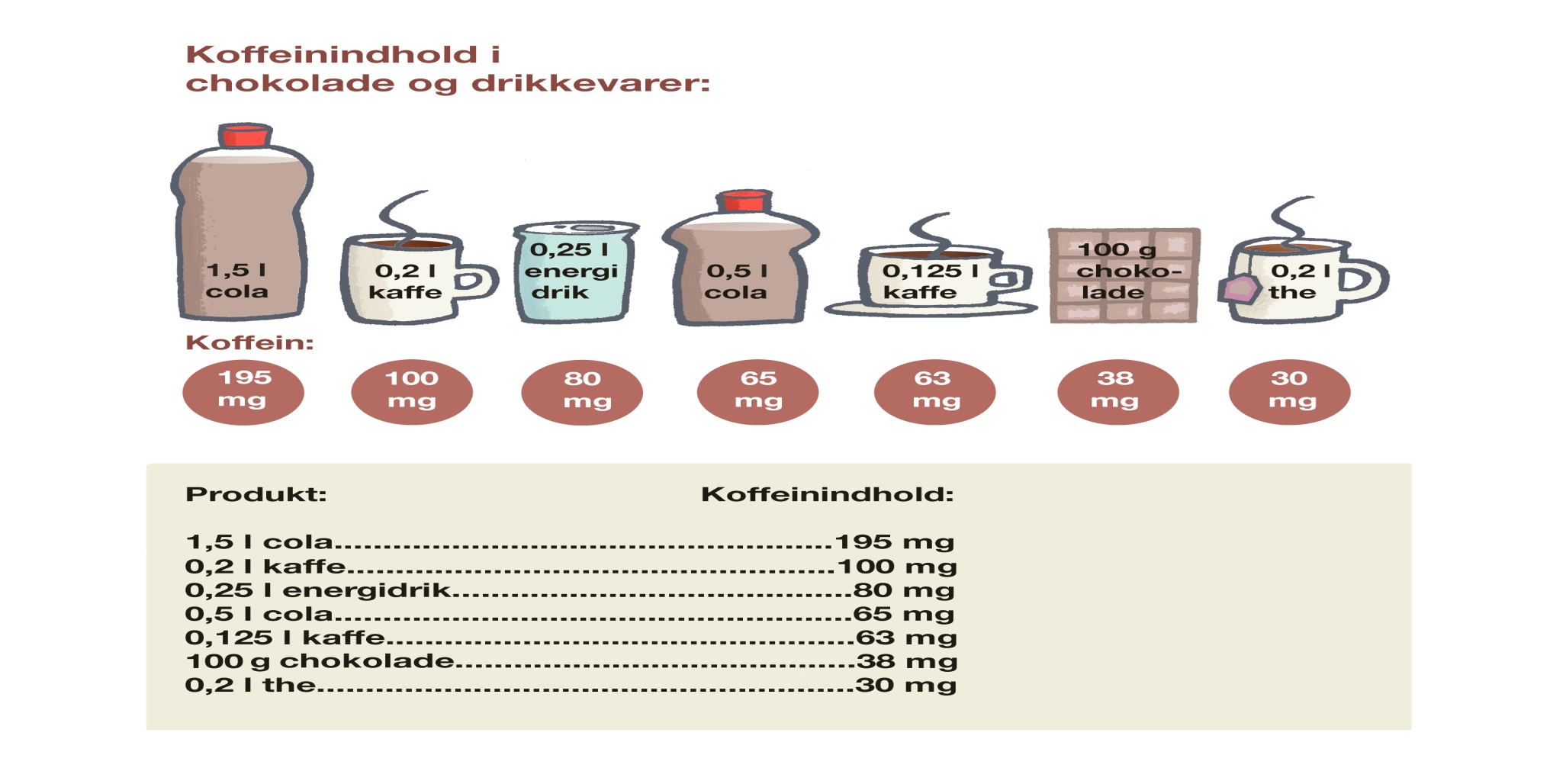


http://www.foedevarestyrelsen.dk/Nyheder/Nyheder/2010/Sider/Ny_information_til_skoleboern_om_energidrikke.aspx

**Sødestoffer**

I mange light produkter erstattes det raffinerede sukker med en række sødestoffer for at nedsætte energiindholdet. De mest almindelige sødestoffer er saccharin, aspartam, sobitol og xylitol.

Den mest omtalte er aspartam (E951) også kendt som NutraSweet (det amerikanske navn). Aspartam bruges i sodavand, læskedrikke, yoghurt, marmelade, is og slik. Desuden må aspartam bruges i marinader til fisk og krebsdyr, saucer, slankekostprodukter, vitamin- og mineraltabletter, øl og cider.

Aspartam indeholder det samme antal kalorier pr. gram, som sukker, men da det er ca. 200 gange sødere end sukker, behøver man kun at tilsætte meget små mængder aspartam for at opnå en sød smag. Derfor har fødevarer og drikkevarer, sødet med aspartam et lavere energiindhold end tilsvarende produkter sødet med sukker( Fødevarestyrelsen 2011).

Sødestoffet aspartam er sammensat af to aminosyrer, som findes naturligt i proteinerne i vores kost. Det fordøjes i den menneskelige organisme på samme måde som proteiner, idet sødestoffet er fremstillet af aminosyrer, som er proteinbestanddele.

Aspartam er godkendt som tilsætningsstof i EU efter en sundhedsmæssig vurdering. Godkendelsen beror på omfattende videnskabelige undersøgelser af stoffet, der viser, at det ikke er sundhedsskadeligt for mennesker, herunder at stoffet ikke kan give skader på arveanlæg, forstyrrelser i forplantningsevnen eller kræft.

Den daglige acceptabel indtagelse af fx aspartam er fastsat til 40 mg dagligt per kilo kropsvægt. En liter sodavand sødet med aspartam må højst indeholde 600 mg aspartam. En person, der vejer 60 kilo, kan drikke 4 liter sodavand med aspartam eller spise ca. 1 kilo slik med 2.000 mg aspartam/kg, før den acceptable daglige indtagelse bliver overskredet. Det er dog vigtigt at være opmærksom på, at mange andre dagligvarer også indeholder aspartam. (DTU Fødevareinstituttet).

Søde – men sukkerfri produkter kan snyde hjernen. Den søde smag får hjernen til at tro at der kommer sukker, og det udløser en uforholdsmæssig stor mængde insulin. Det får blodsukkeret til at falde og udløser en sultfølelse (Ingvar og Eldh, 2010). Derfor vil en sødede drik få kroppen til at kompensere med mad som har et stort kalorie indhold( fedtrig kost) og der vil ske en overkompensering (Knudsen).

Spiser og drikker vi sødede produkter gennem længere tid, vil det på sigt forårsage problemer med kroppens ligevægtssystem. Det holder op med at koble mad med insulinbehov. Konsekvensen bliver, at insulin frigøres for sent, og at blodsukkeret stiger meget brat – selvom der indtages almindelig kost (Ingvar og Eldh, 2010).

## REFERENCELISTE

Andersen, L og Pedersen, B (2011), Fysisk aktivitshåndbogen - forebyggelse og behandling

Astrup, Arne m.fl. (2002) Menneskets ernæring

Becks, Judith S(2006) Kognitiv terapi – teori, udøvelse og refleksion

Borg T & Runge U & Tjørnov J (2003) Basisbog i ergoterapi – aktivitet og deltagelse i hverdagslivet

Cold, J (2007) Kost og psykiatri om sammenhæng mellem kost og psykisk sygdom

DTU Fødevareinstituttet, Den lille levnedmiddeltabel, 4 udgave, 1. oplag, 2012

Garrow Js. et. al (2003)Human Nutrition and Dietetics

# Greenberger, D & Padesky, C (2008) Følelser med fornuft

Holmsted, K & Hornnes, N (2006) Fysisk aktivitet som forebyggelse af vægtøgning hos patienter med skizofreni

Hedergaard, Gustav (2002) Human ernæring

Henning, Margrethe (2001) Projektrapport, Pårørendearbejde i voksenpsykiatrien

Hilsted, Jannik & Borch-Johnsen, Knut & Sandahl Christiansen Jens: (2. udgave 2011) Diabetes

Ingvar, Martin & Eldh, Gunilla (2008) Hjernen styrer vægten

Lind, M & Jaspers, K & Kjær, N (2009) Rygeafvænning

Kristensen, S & Viggers, L & Christensen, Stubbe A (2011) Din guide til et varigt vægttab

Mikkelsen, Brændgaard Per (2000). Spis som du vil

Miller, R William & Rollnick Stephen (2004) Motivationssamtalen

# Olsen N & Sigurdarson S & Hansen S (2002) Kost og Motion til psykisk syge

Vidensråd for Forebyggelse, Kafferapporten. Juni 2012

Anbefalingerne for den danske institutionskost, 2011

Nordic Nitrition Recommendationer, 2004

FaKD´s Rammeplaner – Diætbehandling af svær overvægt hos voksne

Kostkompasser – Vejen til en sund balance, Fødevarestyrelsen 2010

10 veje til vægttab – sundhedsstyrelsen

Små skridt til vægttab – der holder - Sundhedsstyrelsen

Dansk cardiologisk Selskab og Dansk Endokrinologisk Selskab: Diabetes og hjertesygdom. DCS vejledning 2008. Nr. 2

[www.foedevarestyrelsen.dk](http://www.foedevarestyrelsen.dk/) , (2011) Sødestoffer

[www.dagenssundhed.dk/kost-og-ernæring](http://www.dagenssundhed.dk/kost-og-ernæring) Charlotte Hartvig, Styr på sødestofferne

[www.videnskab.dk](http://www.videnskab.dk/) Alicja Mortensen(2012) Er sødemidlet aspartam i light-sodavand skadeligt?

[www.foodcomp.dk](http://www.foodcomp.dk/) DTU Fødevareinstituttet

<http://da.wikipedia.org/wiki/Koffein>

[www.go-motion.dk](http://www.go-motion.dk/) Knudsen Tom (2003) **Feder kunstige sødemidler - ja eller nej?**

[www.hjerteforeningen.dk](http://www.hjerteforeningen.dk/) Anne Skjødt (2012)

www.op- i - røg.dk kræftensbekæmpelse

Artikler:

Prochaska & Diclemente (år) Towards a comprehensive model of change

Verhaeghe, Maes et al., (2011) Effectiveness and cost-effectiveness of lifestyle interventions on physical activity and eating habits in persons with severe mental disorders

Sloth, Birgitte (2008) Appetitregulering – et komplekst system
