# Supplementary material for: Protocol for CHANGE: a randomized clinical trial assessing lifestyle coaching plus care coordination versus care coordination alone versus treatment as usual to reduce risks of cardiovascular disease in adults with schizophrenia and abdominal obesity
Source: BMC Psychiatry. 2015 May 23;15:119. doi: 10.1186/s12888-015-0465-2 (PMC4460642; doi:10.1186/s12888-015-0465-2)
Supplement: Additional file 3: — Physical activity manual. [file 12888_2015_465_MOESM3_ESM.doc]

**Indholdsfortegnelse**

1. Baggrund, Formål, Metode

2. Samtale omkring fysisk aktivitet

3. Førovervejelsesfasen

4. Overvejelsesfasen

5. Forberedelsesfasen

6. Handlingsfasen

7. Vedligeholdelsesfasen

8. Tilbagefald

*9.* Arbejdsark

**Teori og metode**

10. Sundhedsstyrelsens anbefalinger for fysisk aktivitet

11. Definitioner på fysisk aktivitet, intensitet og Borgskala

12. Fordele ved at bevæge sig lidt mere og fordele ved høj intensitet

13. Fysisk aktivitet og energiforbrug

14. Forholdsregler ved opstart på fysisk aktivitet

15. Fysiske test ved baseline: Taljemål, BMI, konditest, Physical Activity Scale (PAS).

16. Skizofreni & fysisk aktivitet, motion og psyke, kropslige sansemæssige forstyrrelser

17. Kroppen i forandring - hvad sker der i kroppen når man træner ?

18. Netværkets betydning for deltagelse i motionsaktiviteter

19. Målsætning - SMART

20. Træningsdagbog

21. Træningsprogrammer

22. Testning

23. Motion i nærmiljøet

24. Fysisk aktivitet i grupper

25. Vidensbank med referencer (SST, forskningsartikler, links til hjemmesider m.m.)

**Baggrund**

Denne manual omkring fysisk aktivitet ”Fysisk aktivitetsmanualen” er udarbejdet i forbindelse med ”Projekt CHANGE: Det gælder livet”, en lodtræknings undersøgelse af systematisk intervention for at bedre psykisk syges helbredstilstand.

Formålet med projekt CHANGE: Det gælder livet er at undersøge, om en individualiseret og målrettet livsstilsintervention og øget fokus på behandling af fysiske sygdomme kan bedre den fysiske helbredstilstand hos patienter med skizofreni. Projektet fokuserer på rygestop, fysisk aktivitet, sunde kostvaner og monitorering af helbredstilstand.

Patienter med diagnosen skizofreni som har været indlagt, har en 20 år kortere forventet levetid end personer i baggrundsbefolkningen, som aldrig har været indlagt på en psykiatrisk afdeling. Den vigtigste årsag er formentlig, at patienter med skizofreni ofte har en usund livsstil herunder især et meget lavt fysisk aktivitetsniveau. Hertil kommer at mange patienter med skizofreni ikke i tilstrækkelig grad undersøges og behandles for somatiske sygdomme (f.eks. type 2 diabetes, hypertension, og dyslipidæmi) samt at nyere antipsykotisk medicin kan give øget risiko for vægtøgning og forstyrret hjerterytme.

Deltagerene i projektet får tilknyttet en livsstilscoach. Livsstilscoachens opgave er støtte deltageren i at gennemføre livsstilsændringer som har betydning for deres helbred. Herudover at sikre at deltageren undersøges og behandles for somatisk sygdom og indikatorer på øget risiko for hjertekarsygdom, diabetes, KOL og andre livsstilsrelaterede sygdomme.

**Formål**

Formålet med denne manual er dels at beskrive hvorledes fysisk aktivitet bliver brugt som intervention i projekt CHANGE: *Det gælder livet*, og herudover at udarbejde en praktisk anvendelig manual med konkrete redskaber til livsstilscoachens kliniske arbejde omhandlende fysisk aktivitet.

Første del af manualen er lavet som inspiration til hvorledes livsstilscoachen kan komme i dialog med deltageren omkring fysisk aktivitet samt støtte deltageren i gennem de forskellige faser i motivationsprocessen.

Anden del vil fortrinsvis være baggrundsviden og materiale som livsstilscoachens teoretiske fundament bygger på.

**Metode**

Idet følgende beskrives kort den *Assertive tilgang* og *Motivational Interviewing* da disse metoder danner grundlaget for livsstilscoachens arbejde.

**Assertiv tilgang**

Baggrunden for udviklingen af metoden er erkendelsen af, at den svært psykisk syge ikke blot har brug for et katalog over muligheder, men også en opsøgende behandling og støtte og ledsagelse for at kunne udnytte de muligheder, lokalsamfundet indeholder.

ACT ift. kontakten til deltagerne i CHANGE:

- Den assertive tilgang muliggør fastholdelse af kontakt gennem en opsøgende, fleksibel og vedholdende og respektfuld tilgang.
- Den faste tilknytning til en enkelt ”livsstilscoach” gennem et år, er med til at sikre at alliancen til deltageren i høj grad fastholdes.
- Understøtte at deltagerne møder i gruppetilbud i CHANGE.
- At tilbyde fleksibel kontakt ved f.eks. hjemmebesøg, møder i lokalområdet eller via mail/telefon.
- At stå for individuel tilpasset kontakt med den enkelte deltager. I kritiske perioder kan kontakten være mere hyppigt.
- Kontakt aftales på deltagerens præmisser.
- Samarbejde med den behandlingsansvarlige enhed.
- Understøtte kontakten til praktiserende læge, for at sikre behandling af somatiske lidelser.
- At engagere og arbejde aktivt med deltagerens netværk i bred forstand (f.eks. bofællesskab, familie, arbejds og fritidskontakter), for at sikre at deltageren vedligeholder ændringer af vaner i forhold til motion, kost og rygning.

**Motivational interviewing (MI)**

Motivational interviewing på dansk motivationssamtalen, er en evidensbaseret tilgang, som indeholder en lang række teknikker, der samlet skal understøtte, at deltageren bliver i stand til at udvinde og udnytte sin egen motivation for ændringer i livsstil. Metoden omfatter desuden redskaber til understøttelse af vedligeholdelse af adfærdsændringer og håndtering af tilbagefald.

MI anvendes lige fra 1. samtale og hele vejen gennem forløbet, og er gennemgående i samtlige interventionstilbud i CHANGE.

I MI er det essentielt, at man arbejder på at forstå klientens perspektiv, syn på sig selv og sit liv.

Som livsstilscoach arbejdes der med MI, som består i, at man gennem et samarbejde med deltageren frembringer dennes argumenter for forandring i en samtaleånd, der bygger på deltagerens autonomi.

- Samarbejde ikke konfrontation
- At frembringe ikke installere
- Autonomi ikke autoritet

MI bygger overordnet på 4 principper, som er vigtige i arbejdet som livsstilscoach.

De 4 principper er:

- Udtryk empati
- Udvikling og tydeliggørelse af diskrepans
- Støtte troen på egen mestring
- Undgå diskussion og gå med modstanden

For yderligere baggrund omkring MI henvises til bogen: ”Motivationssamtalen”; William R. Miller & Stephen Rollnick, Hans Reitzels Forlag, København 2004

**Del 1. Samtale omkring fysisk aktivitet**

Når patienten kommer til samtale omkring fysisk aktivitet, har livsstilscoachen resultaterne vedrørende PAS (Physical Activity Scale), kondital, taljemål, lipidstatus, m.m. Disse resultater kan gennemgås og danne udgangspunkt for samtalen. Formålet med samtalen/samtalerne er at undersøge og fremkalde deltagerens motivation for ændring i forhold til fysisk aktivitet. Sammen med deltageren skal livsstilscoachen undersøge mulighederne for, at indplacere et større element af fysisk aktivitet i hverdagen, som er attraktiv, acceptabel, realistisk og meningsfuld for deltageren. Herudover kan livsstilscoachen informere om (hvis deltageren ønsker det) den eventuelle identificerede sundhedsmæssige risiko ved et lavt fysisk aktivitetsniveau og lavt kondital m.m. samt hvorledes fysisk aktivitet kan bidrage til en øget sundhed.

Overordnet arbejdes der på at understøtte motivationsprocessen hen imod et øget fysisk aktivitetsniveau udfra følgende:

- Undersøg deltagerens perspektiv på sundhed. Hvilke værdier og holdninger har deltageren i forhold til sundhed og fysisk aktivitet? Hvad er sundhed for deltageren? Denne snak er vigtig i den/de første samtaler med deltageren. (se evt. 1. samtale)
- Klarlæg og opnå accept af behovet. Deltageren skal opleve/erkende og acceptere et behov. Snak evt. om test resultater - hvad tænker deltager om disse?
- Forklar resultater (husk at spørge om lov) og om sammenhæng mellem fysisk aktivitet og sundhed - både generelt og specifikt i forhold til deltagerens diagnoser.
- Undersøge nuværende og tidligere erfaring med fysisk aktivitet, idræt/sport/motion.

Afdæk aktuelle fysiske aktivitetsniveau ved hjælp af f.eks. Physical activity scale (PAS) eller hverdagsbeskrivelse.

- Klarlæg holdningen til fysisk aktivitet.

Arbejd med skala-spørgsmål. (vigtighed, evne og parathed)

- Undersøg ønsker, behov og muligheder for fysisk aktivitet
- Undersøg og afdæk ambivalensen, herunder barrierer for fysisk aktivitet samt nogle strategier for at overvinde disse. (beslutningsbalancen).
- Tag stilling til risikofaktorer og kontraindikationer (se afsnit: ”Forholdsregler ved opstart på fysisk aktivitet”)
- Fastlæg aktivitetsform og -niveau sammen med deltageren.
- Undersøg behov for hjælp og støtte. Hjælp med at løse eventuelle praktiske problemer og giv støtte, ros og anerkendelse undervejs.
- Lav SMART(e) mål
- Udarbejd plan for igangsætning/afprøvning.

## Førovervejelses-fasen

Deltageren er ikke parat til at ændre adfærd. Det er ofte andre, feks bostøtte, primærbehandler, læge eller familie der har rejst problematikken og lagt op til at deltageren gør noget ved f.eks vægten og et lavt fysisk aktivitetsniveau.

Deltageren udviser modstand mod forandring og modvilje mod at tale om det: *”Hvorfor skal jeg altid høre om...?”*

Deltageren med overvægt mener måske ikke, at fysisk inaktivitet er årsag til sundhedsmæssige problemer, og/eller det kan være en demonstration af, at denne selv bestemmer. Det kan være baseret på en personlig, men realistisk overvejelse af fordele og ulemper ved at fortsætte hidtidige vaner. Det kan dog også være baseret på en urealistisk vurdering af de risici, der er forbundet med at være fysisk inaktiv, samt manglende tro på egen evne i forhold til at ændre vane. En opfattelse der kan have sin rod i tidligere skuffelser og erfaringer med at være fysisk aktiv og/eller dyrke motion.

**Hovedopgave:**

- Målet er at få deltageren til at reflektere over sin situation.
- Afdække nuværende og tidligere erfaringer med fysisk aktivitet og motion/idræt

**Strategi:**

- Vær ikke konfronterende eller argumenterende, spørg hellere:

*”Dyrker du, eller har du dyrket, nogen form for fysisk aktivitet? - hvis ja i såfald hvad og hvordan og hvorfor?”*

*”Hvorfor stoppede du?”*

*”Hvordan har du det med at være fysisk aktiv?”*

*”Har du overvejet at blive mere fysisk aktiv/dyrke motion?” , -*

*”Hvad var grunden til at du tænkte på at blive mere fysisk aktiv/dyrke motion?”*

- Spørg om nødvendigt både ind til dagligdags rutiner, som kræver fysisk aktivitet (rengøring, indkøb, trappegang, cykling), og til eventuelle erfaringer med systematisk træning, fx sport, hvor pulsen kommer op, hvor man bliver meget forpustet.
- Deltagere der ikke er opmærksomme på, at der kan være en sammenhæng mellem fysisk inaktivitet og livsstilsrelaterede sygdomme kan man sige:

*”Der er en sammenhæng mellem fysisk aktivitet og forhøjet blodtryk (eller overvægt m.m.).*

*- Er det noget du gerne vil have, at jeg fortæller noget mere om?”*

- Det er vigtigt at information om risici følges op af konkrete tilbud om støtte og vejledning.

**Arbejdsark: Registrering af deltagers erfaring med fysisk aktivitet**

## Overvejelses-fasen

Deltageren anerkender, at der er et problem og tænker på forandring, men er ikke kommet længere og gør ikke noget for at komme videre. Deltageren er indstillet på, at der skal ske noget - **bare ikke lige nu***: ”Jeg ved godt, at jeg burde komme i gang med at motionere. Jeg gør det også en dag, men ikke lige nu. Der er så meget andet for tiden”.*

Denne fase og forberedelsesfasen er de vigtigste for motivationen – her dannes grundlaget, lysten, viljen og modet til at gå i gang.

Undersøg, om deltageren har barrierer for at være fysisk aktiv, fx nedsat bevægefunktion, smerter m.m. Herudover om angst, negative symptomer og/eller sansemæssige kropslige forstyrrelser (se afsnit omkring skizofreni & fysisk aktivitet) kan gøre det svært at være fysisk aktiv.

Undersøg **vigtighed, evne og parathed** ift. ændring.

**Hovedopgave:**

- Afklaring af deltagerens ambivalens i forhold til fysisk aktivitet, herunder barrierer for fysisk aktivitet samt strategier til at overvinde disse. Reflekter forandringsudsagn og diskrepans.

**Strategi:**

- Prøv at finde ud af hvordan deltageren opfatter problemet og suppler eventuelt dennes viden med din sundhedsfaglige vurdering:

*”Hvordan vil du selv beskrive dit problem?”* eller *”Hvad tror du, det kan komme til at betyde for dig?”, ”Er du interesseret i at høre noget mere om det?”*

Spørg, om deltageren er interesseret i at forbedre sin kondition. Snak eventuelt med deltageren om fordelene ved at have en god kondition: glæde ved at bevæge sig, mere overskud i hverdagen, mindre sygdomsrisiko, bedre vægtregulering osv. Når deltageren er konditionstestet, kan man tage en snak om overensstemmelsen mellem testresultatet og patientens egen vurdering.

*”Hvordan synes du, at din kondition er?”*

Formålet med ovenstående spørgsmål er at vurdere deltagerens forhold til sin egen kondition samt at vurdere, om dette forhold er realistisk.

- Hvordan bedømmer deltageren selv sin prognose:

*”Hvad tror du, der vil ske, hvis du ikke gør noget ved det?”*

*”Hvad frygter du mest?”*

- Få deltageren til at reflektere over diskrepansen mellem ønsker og aktuelle adfærd.
- Brug eventuelt Skala-spørgsmål omkring vigtighed, evner og parathed. (se vedlagte arbejdsark). Udfra svarene kan man bruge forandringscirklen til at tale med deltageren om, hvor denne er i sin proces og herudfra foreslå videre plan for forløbet.
- Brug eventuelt Beslutningsbalance, (se vedlagte arbejdsark)
- Ingen forsøg på overtalelse!

Som opfølgning på at skabe klarhed over beslutningsbalancen og for at påbegynde en konkretisering af de praktiske konsekvenser i forbindelse med opstart på fysisk aktivitet kan man spørge:

*”Hvis du nu besluttede dig for at begynde med at løbe...” (eksempelvis):*

*”Hvad ville du miste ved det? - eller hvad skulle du undvære?” (ulemper)*

*”Hvad ville det medføre af positive ting?” (fordele)*

## Afslutningen på samtalen:

Opsummer hvad der er blevet talt om og spørg eventuelt:

- *”Hvad har været vigtigst for dig af de ting, vi har talt om i dag?”*
- *”Hvad kunne du tænke dig at gå videre med efter vores samtale?”*
- *”Vil du være med til, at vi mødes igen om en uge?”*

*”WORKING WITH AMBIVALENCE IS WORKING WITH THE HEART OF THE PROBLEM”*

Miller & Rollnick

## Arbejdsark: Skala-spørgsmål

**Arbejdsark: Beslutningsbalance**

**Arbejdsark: Forandringscirklen**

**Skala-spørgsmål**

**Hvor vigtigt er det for dig at du... ? (fx blive mere fysisk aktiv, tabe dig.. ..)**

**0_________________________________________________________________________________10**

**Hvordan vurderer du dine muligheder/evner/tiltro for ændringen....? (fx blive mere fysisk aktiv, tabe dig...)**

**0__________________________________________________________________________________10**

**Hvor parat er du til at gå i gang med at...? (fx dyrke motion....)**

**0________________________________________________________10**

**Beslutningsbalance**

| **Fordele ved det aktuelle (fx ikke at dyrke motion)** | **Ulempe ved at ændre vaner (fx at dyrke motion)** |
| --- | --- |
| **Fordele ved at ændre vaner (fx at dyrke motion)** | **Ulemper ved det aktuelle (fx ikke at dyrke motion)** |

**Når du nu efterfølgende kigger på skemaet med dine gode overvejelser omkring fordele og ulemper ved en eventuel ændring (eller ved ingen ændring), hvad tænker du så?**

**Hvad er dine 3 hovedgrunde til at…? (f.eks. at begynde at løbe):**

**1.___________________________________________________________**

**2.___________________________________________________________**

**3.___________________________________________________________**

## Forberedelsesfasen

Deltageren har besluttet, at der skal gøres noget og skal definere hvad han/hun gerne vil opnå. Målet skal fastlægges før, man begynder at diskutere, hvad der skal gøres. Deltageren skal forklare, hvad det er han/hun vil opnå med at at blive mere fysisk aktiv . Hvis deltageren er motiveret, men har svært ved at komme i gang, kan man spørge ind til handlinger fremfor motiver: Hvordan vil deltageren nå sine mål ?

Hvis deltageren ønsker det, kan man informere om fysiske aktivitetstilbud m.m.

**Hovedopgave:**

- At stimulere til at deltageren finder konkrete og realistiske løsninger på sine problemer i forbindelse med opstart på fysisk aktivitet.
- Mål og plan drøftes med deltageren.

**Strategi:**

- *”Hvordan vil du gribe det an?”* - Bekræft deltageren i at dennes ressourcer og færdigheder er tilstrækkelige til at gennemføre det aftalte.
- *”Hvilke former for motion kunne du tænke dig at dyrke?”*
- *”Hvad er det første der skal ske?”* - Få deltageren til at være helt konkret, også i detaljerne.
- *”Hvordan er dine muligheder?” -* Stimuler deltageren til at overveje realistiske muligheder og alternativer.
- *”Hvilke problemer tror du der kan opstå?”* - Forudse problemer og giv støtte til at finde løsninger.
- *”Hvad kunne gøre det lettere for dig at gennemføre det?”*
- *”Hvem eller hvad kunne hjælpe dig ? - og hvordan?”* Netværket og livsstilscoach kan eventuelt være en afgørende ressource. Hjælp deltageren til at identificere og mobilisere sine muligheder for støtte. (se afsnit ”X” omkring ”Netværkets betydning”)
- *”Hvis du en dag har mest lyst til ikke at røre dig, hvad kan du/jeg/andre så gøre for at få dig med ud at være aktiv alligevel? - Nævn 3 ting, som du selv kan gøre for at få dig til at holde fast ved motionen”.*

Formålet med spørgsmålet er at få deltageren til at reflektere over og sætte ord på,

hvad der kan få ham/hende til at blive ved med at være fysisk aktiv, selv når motivationen svigter.

Foretrækker deltageren at motionere alene eller sammen med andre? Hvis deltageren foretrækker at være aktiv sammen med andre, så vurder, om det er af betydning, at denne kender de øvrige personer.

Prøv at afdække vigtigheden af afstand til stedet, hvor der er mulighed for at være

fysisk aktiv, samt om økonomi spiller en rolle for motivationen. Spørg også, om

det er vigtigt at have nogen at følges med fra hjemmet eller bostedet til

træning (evt. bostøtte/en ven).

- *”Hvad skal målet være for din motion på kort sigt og på lang sigt?”*

Formålet med spørgsmålet er at kunne lave en motionsplan, der motiverer deltageren bedst muligt for deltagelse i fysisk aktivitet og motionstilbud.

Her kan der spørges om, *hvilke drømme/visioner deltageren ville have for sin egen*

*fysiske form, hvis der ikke var nogen begrænsninger*.

Skitser eventuelt en motionsplan sammen med deltageren i form af et ugeskema. Opstil gerne mål og målbare delmål med en aftalt tid. Det kunne fx være: Om 14 dage (dato) kan jeg være med til at spille bold i 20 minutter uden at stoppe. Eller: Om en uge kan jeg gå en aftentur med et enkelt hvil.

Benyt eventuelt skalaspørgsmål:

- *”Hvor sikker er du på at kunne gennemføre din motionsplan?”*

Formålet med spørgsmålet er at vurdere deltagerens tro på at kunne gennemføre

motionsplanen og få diskuteret evt. barrierer samt nogle strategier for at overvinde

dem. Hvis patienten er usikker eller ikke mener at kunne gennemføre motionsplanen, tales

om barrierer og strategier for at overvinde disse, og motionsplanen justeres efter dette.

## Arbejdsark: Målsætning med SMART mål

MÅLBESKRIVELSE Dato:

SMART mål Navn:

| Navn på mål: | |
| --- | --- |
| Hvordan hænger dette mål sammen med mine andre mål og værdier i livet? | |
| S  Specifikt | Formuler målet så præcist og detaljeret som muligt. |
| M  Målbart | Hvordan kan jeg se at målet er opfyldt? Er der delmål undervejs? |
| A  Attraktivt | Hvor attraktivt er målet (1-10)? Er det et middel til at nå andre mål ? |
| R  Realistisk | Hvad taler for og i mod at målet kan nås? Hvor realistisk er målet (1-10)? |
| T  Tidsbestemt | Hvornår er målet opfyldt? (dato, tid, periode) Hvad med delmål? |
| Hvem og hvad kan støtte mig i at nå målet? - og hvordan ? | |
| Hvad er det første skridt, jeg skal tage for at nå målet? | |

**Handlingsfasen**

Deltageren er i gang med fysisk aktivitet. Informér om muligheder (se afsnittet: Motion i nærmiljøet), som deltageren ikke selv er opmærksom på. Vær parat med gode råd om midler til at nå målet, men lad deltageren selv afgøre om rådene kan bruges til noget. Lad hellere deltageren selv komme med forslag. Anerkend og ros deltageren konkret for det denne har gjort godt. Snak om løsninger på de problemer der eventuelt kan opstå.

**Hovedopgave:**

- Hjælp deltageren med at nå frem til den/de mest egnede løsning(er).
- Styrk deltagerens selvtillid og ressourcer.

**Strategi:**

- Få deltageren til at identificere vanskelighederne:

*”Hvad har været vanskeligt ved at lave fysisk aktivitet?”*

*”Hvordan lykkes det dig at komme videre?” -* Anerkendelse og ros.

- Fokuser på ressourcer:

*”Hvad er du god til?”*

- Deltageren, ikke du skal vurdere, om rådene kan bruges:

*”Tror du, du kan bruge mine råd til noget?”*

*”For nogle kan det være en hjælp at....Tror du det er noget du kan bruge?”*

Tilbyd støtte og aftal ny tid. Følg op på mål og delmål (jvf. SMART målsætning) og juster om nødvendigt. Motionsdagbog, ugeskema eller lignende kan kan for nogle fungere motiverende og som redskab til selvevaluering og give feedback.

*”Hvad vil du gøre til vi mødes igen?” -* Præcisér hvad planen er.

**Arbejds ark: Træningsdagbog og ugeskema**

## Vedligeholdelsesfasen

Det er svært at ændre indgroede vaner og være vedholdende. Hjælp deltageren med at identificere risici for tilbagefald og strategier for forebyggelse af tilbagefald.

**Hovedopgave:**

- Det gælder om at forebygge tilbagefald, dvs. blive ved med at være fysisk aktiv. Støtte deltageren og styrke ressourcer.

***Strategi:***

- Anerkend deltagerens indsats:

*”Jeg kan mærke på dig, at du er meget opsat på, at det skal lykkes denne gang”*

- Ros deltageren for det denne gør og undgå at fortælle at det kunne være bedre:

*”Jeg synes det er rigtig flot/sejt, at du er begyndt at gå en tur hver dag!”*

- Få deltageren til at gentage hvorfor denne besluttede sig for at være fysisk aktiv:

*”Hvorfor var det nu, du besluttede dig for at begynde at cykle frem for at tage bussen?”*

- Få deltageren til at beskrive sin indsats og sine vanskeligheder:

*”Hvad oplever du er det sværeste ved at...?”*

- Giv deltageren mulighed for at fortælle om sine succeser:

*”Hvad er du selv mest stolt af?”*

- Få deltageren til at identificere risiko for tilbagefald:

*”Hvad frygter du mest?”*

*”Hvad vil du gøre, hvis du en dag føler at du ligeså godt kan opgive?”*

Få deltageren til at tænke situationen igennem og gør opmærksom på at du måske netop i den situation kan støtte deltageren og at denne derfor skal komme selvom det er ved at gå skævt.

- **Fokuser på kropslige erfaringer** (se arbejdsark: ”Subjektiv oplevelse af fysisk aktivitet”). Herved kan man gennem konkret refleksion i forhold til hvad deltageren mærker i kroppen, være med til at øge kropsbevidstheden hos denne, samt få vigtig information om deltagerens kropslige erfaringer (”forandrings-oplevelser”) i relation til fysisk aktivitet. Deltageren får samtidig mulighed for at verbalisere sine kropslige oplevelser. Disse erfaringer kan bruges som pædagogisk redskab i motivationsarbejdet. Spørgsmålene kan bruges både som hjemmearbejde og når man konkret laver fysisk aktivitet med deltageren.

**Arbejdsark: Subjektiv oplevelse af fysisk aktivitet**

**Subjektiv oplevelse af fysisk aktivitet**

Navn:........................................................................... Dato:.......

Før fysisk aktivitet: Hvordan har du det i kroppen lige nu?

.......................................................................................................................................

........................................................................................................................................

........................................................................................................................................

.......................................................................................................................................

Marker på skalaen:

0..........1..........2..........3..........4..........5..........6..........7..........8..........9.........10

0 = Så dårligt som du kan have det.

10 = Så godt som du kan have det.

Efter fysisk aktivitet: Hvordan har du det i kroppen lige nu?

........................................................................................................................................

........................................................................................................................................

........................................................................................................................................

......................................................................................................................................

Marker på skalaen:

0..........1..........2..........3..........4..........5..........6..........7..........8..........9.........10

0 = Så dårligt som du kan have det.

10 = Så godt som du kan have det.

Hvordan har det været for dig at være med til fysisk aktivitet i dag?

........................................................................................................................................

........................................................................................................................................

........................................................................................................................................

........................................................................................................................................

## Tilbagefald

De fleste der forsøger at ændre vaner hen imod en mere fysisk aktiv livsstil vil opleve tilbagefald i perioder. Tilbagefald skal ikke defineres som ”nederlag”, men som episoder man kan lære noget af. Der er vigtigt at deltageren kommer videre og ikke låses fast i denne fase. Anerkend det deltageren har gjort eller forsøgt at gøre indtil nu. Målet skal eventuelt reformuleres.

**Hovedopgave:**

- Hjælpe deltageren videre i forandringscirklen.
- At lære af tilbagefaldet.

***Strategi:***

- Fokuser på deltagerens erfaringer:

*”Hvad var det der skete?”*

*”Hvad skal der til for at holde fast i din beslutning om at komme afsted til...næste gang der opstår problemer?”*

*”Hvad er det, der gør det så svært?”*

- Analyser årsagerne til tilbagefaldet og bliv opmærksom på nye strategier:

*”Hvad gik der galt?”*

*”Når du ser tilbage på forløbet, er der så noget du ville have gjort anderledes?”*

*”Hvad skal der til for at undgå et nyt tilbagefald?”*

*”Hvad skal der til for at komme i gang igen?”*

- Snak eventuelt med deltageren om forandringscirklen (se arbejdsark) og fortæl at det er normalt at bevæge sig frem og tilbage i denne. Fortæl om forskellen på en ”svipser” og et tilbagefald. Hvad tænker deltageren om dette ?

**Arbejdsark: Forandringscirklen**

**Del 2. Teori**

I denne del beskrives overordnet dele af de teoretiske referencer som danner udgangspunktet for livsstilscoachens arbejde med fysisk aktivitet. I projekt CHANGE: det gælder livet, arbejdes der udfra Sundhedsstyrelsens anbefalinger for fysisk aktiviteti forhold til voksne og overvægtige:

**Sundhedsstyrelsens anbefalinger for fysisk aktivitet**

**Anbefalinger til voksne (18-64 år)**

(kilde: SST hjemmeside)

- Vær fysisk aktiv mindst 30 minutter om dagen. Aktiviteten skal være med moderat til høj intensitet og ligge ud over almindelige kortvarige dagligdags aktiviteter. Hvis de 30 minutter deles op, skal aktiviteten vare mindst 10 minutter.
- Mindst 2 gange om ugen skal der indgå fysisk aktivitet med høj intensitet af mindst 20 minutters varighed for at vedligeholde eller øge konditionen og muskelstyrken. Der skal indgå aktiviteter, som øger knoglestyrken og bevægeligheden. Almindelige kortvarige dagligdags aktiviteter defineres i denne sammenhæng som de aktiviteter, man hyppigt udfører i dagligdagen af kort varighed (under 10 minutter) uanset deres intensitet.

Fysisk aktivitet ud over det anbefalede vil medføre yderligere sundhedsmæssige fordele. Fysisk aktivitet dækker over alle former for bevægelse, der øger energiomsætningen. Fx bevægelse på arbejdspladsen, bevægelse i hjemmet, aktiv transport og indkøbsture i supermarkedet m.m.

Moderat fysisk aktivitet dækker alle former for ustruktureret aktivitet/motion, hvor pulsen skal op, og hvor du kan tale med andre imens. Eksempler på fysisk aktivitet af moderat intensitet: cykle og gang til og fra arbejde, havearbejde, trappegang, en joggetur og motionsidræt. Minimumsgrænsen for moderat fysisk aktivitet svarer til en gennemsnitshastighed på 4 km/t.

Fysisk aktivitet af høj intensitet kan være planlagt træning/fysisk aktivitet, der gennemføres to gange om ugen af 20–30 minutters varighed for at forbedre og/eller vedligeholde kondition. Høj intensitet betyder, at pulsen stiger, så du føler dig forpustet og har svært ved at føre en samtale. Eksempler på fysisk aktivitet af høj intensitet kan være svømning, løb, spinning, styrketræning og boldspil m.m.

De 30 minutters fysisk aktivitet kan indgå som en del af tilværelsen og i forbindelse med éns vanlige gøremål. Det kræver således ikke nødvendigvis, at man iklæder sig træningstøj eller tilmelder sig et fitnesscenter. Fysisk aktivitet er også at cykle eller gå til arbejde og supermarked, at tage trappen, at udføre fysisk anstrengende havearbejde, at gøre rent eller at lege aktivt med sine børn mv.

Målrettet motion eller idræt, hvor de store muskelgrupper aktiveres hyppigt og kraftigt, vil selvfølgelig sikre de bedste sundhedsmæssige resultater. Men mindre gavner også. Det handler især om at få nye vaner i hverdagen: mere cykling/gang og mindre bil/buskørsel samt flere trapper og færre elevatorer

**Anbefalinger til overvægtige**

Ønsker du at reducere din kropsvægt, anbefaler Sundhedsstyrelsen en kombination af regelmæssig fysisk aktivitet og lavere energiindtag.

**Forebyggelse af overvægt og svær overvægt**

Fysisk inaktivitet øger risikoen for overvægt og svær overvægt, hvilket medfører andre sundhedsmæssige konsekvenser. Efterlever du Sundhedsstyrelsens anbefalinger for fysisk aktivitet, forebygger du en række livsstilssygdomme relateret til overvægt og svær overvægt. Effekten er størst, hvis fysisk aktivitet kombineres med nedsat energiindtagelse. Fysisk aktivitet forebygger følgende følgetilstande for overvægt og svær overvægt:

- Type 2 diabetes (forbedre insulineffekten)
- Dyslipidæmi (forhøjet koncentration af triglycerid og kolesterol i blodet)
- Forhøjet blodtryk

**Sundhedsstyrelsen anbefaler at:**

- Alle overvægtige og svært overvægtige er fysisk aktive mindst 30 minutter af moderat intensitet, helst alle ugens dage. De 30 minutter kan opdeles i mindre perioder, fx 15 minutter om morgenen og 15 minutter senere, eller 3 gange 10 minutter i løbet af dagen.
- De 30 minutters fysisk aktivitet kan indgå som en del af tilværelsen og i forbindelse med éns vanlige gøremål. Det kræver således ikke nødvendigvis, at man iklæder sig træningstøj eller tilmelder sig et fitnesscenter.
- Muskler, led og sener skal vænne sig til fysisk aktivitet. Start forsigtigt i langsomt tempo og med korte ture.
- Starte med transportmotion: gang og cykling til og fra arbejde, skole, supermarkedet m.m. samt tage trappen i stedet for elevatoren.
- Starte i fladterræn, da det er hårdt for ankler, knæ og hofter, at være fysisk aktiv.
- Huske at bruge ordentligt fodtøj (løbesko eller vandresko), der minimerer anstrengelser på knæ, ankler og hofte. Dvs. tykke såler med god stødabsorbering, snøring som giver mulighed for individuel tilpasning og støtte, og god plads til tæerne i både bredde og højde.
- Tempoet kan øges, når du kan mærke, at konditionen bliver bedre, og dit kredsløb er klar til større udfordringer.
- Fritidsmotion i form af have- og husarbejde, naturudflugter, leg og dans er næste skridt.
- Hvis du ønsker at starte et mere struktureret motionsprogram, bør du vælge aktiviteter, der belaster kredsløbet og de store muskelgrupper. Eksempler på motionsformer: svømning, dans, golf, gymnastik, aerobic, badminton og styrketræning (cirkeltræning).
- Hvis du træner efter et struktureret motionsprogram, bør du de første 5-6 uger ikke motionere mere end tre gange i ugen af 30 minutter.
- Lytte til din krop. Har du ondt i musklerne har kroppen brug for hvile. Har du feber, har kroppen brug for ro.
- Det ikke er nødvendigt, at motionere til udmattelse for at komme i bedre kondition.
- Regelmæssig fysisk aktivitet opretholder konditionen og vægten.
- Du finder en træningsmakker eller flere.

**Sammenhængen mellem overvægt, inaktivitet og fysisk aktivitet**

Overvægt og svær overvægt har både helbredsmæssige, psykologiske og sociale konsekvenser.

- Selvom genetikken spiller en rolle i udviklingen af overvægt og svær overvægt, er der en sammenhæng mellem et for højt energiindtag og et for lavt energiforbrug. Stillesiddende arbejde, energibesparende indretning af arbejdspladser og private hjem, brug af motoriserede transportmidler og inaktiv fritid er afgørende parametre i denne sammenhæng.
- Inaktive har større kropsvægt, BMI og fedtprocent end fysisk aktive.
- Regelmæssig fysisk aktivitet påvirker kropssammensætningen i positiv retning. Styrketræning og udholdenhedstræning øger muskelmassen på bekostning af fedtmassen. Øget muskelmasse fører til øget stofskifte. Øget stofskifte medfører reduktion i kropsvægt.
- Regelmæssig fysisk aktivitet forbedrer evnen til at forbrænde fedt under muskelarbejde.
- Fysisk aktivitet har en positiv indvirkning på appetitreguleringen og insulinfølsomheden.
- Studier har vist, at individer der forsøger at opnå vægtreduktion ved fysisk træning og som fortsætter med at være fysisk aktive efter vægtreduktion, har nemmere ved at opretholde vægttabet.
- Samtidig er der dokumentation for, at sygdomsrisikoen forbundet med overvægt er stærkt reduceret, hvis overvægtige er i god fysisk form.

# Definitioner af fysisk aktivitet

De enkelte deltagere i CHANGE er i forskellig fysisk tilstand når de starter i projektet. Der vil være deltagere, som er fysisk aktive i løbet af dagligdagen og deltagere som dyrker motion og træning som en fast del af deres hverdag.

Erfaringen viser også at en stor del af deltagerne lever et inaktivt liv og hvor fysisk aktivitet ikke indgår som en del af hverdagen.

Der kan være forskellige opfattelser af hvad der er motion og hvad der er fysisk aktivitet, men i CHANGE anvendes Sundhedsstyrelsens definitioner, som er følgende:

1. Begrebet **fysisk aktivitet** dækker ethvert muskelarbejde, der øger energiomsætningen.
   - - - 1. **Motion** bruges både i forbindelse med ustruktureret aktivitet og mere bevidst, målrettet,
         2. regelmæssig træning.
2. **Træning** er planlagt og struktureret fysisk aktivitet, der gennemføres jævnligt for at
3. vedligeholde og/eller forbedre fysisk form og velbefindende.
4. I modsætning hertil er begrebet **inaktivitet**, som betegner en tilværelse uden bevægelse.
5. Inaktive personer indgår ikke i nogen form for fysisk udfoldelse, hverken struktureret eller
6. ustruktureret.

# Definitioner af intensitet i fysisk aktivitet

Når vi møder vores deltagere i projektet er det livsstilscoachens opgave sammen med deltageren at undersøge og planlægge hvilken form for fysisk aktivitet, som er motiverende og hensigtsmæssig for deltageren.

Livsstilcoachen er opmærksom på de fordele og ulemper der er ved forskellige træningsformers intensitetsgrader, og anvender denne viden til at planlægge et hensigtsmæssigt forløb med deltageren, som er motiverende og realistisk.

Vi vil i CHANGE bruge **Borg-skalaen** som er et subjektivt værktøj til at styre og vurdere træningsintensiteten med. Skalaen er anvendelig i forhold til deltageren, og vi vurderer at den er nemmere at anvende og knap så besværlig som andre træningsintensitets redskaber. Det vil være nemmere at instruerer deltageren i hvilken intensitet man skal træne med, for at få det mest optimale udbytte af træningen.

Rent praktisk i CHANGE vil det kun være nødvendigt at skulle skelne mellem nogle udvalgte niveauer på skalaen. Specielt udgør Borg 15 grænsen mellem det, som de fleste vil opfatte som moderat eller hård træning. Samtidigt er det også det niveau, de fleste skal stræbe efter at opnå i deres almindelige motionsaktiviteter. Ved at bruge beskrivelserne i skemaet nedenfor bliver det rimeligt enkelt at vurdere for deltageren, hvor man er henne og dermed kunne vurdere f.eks. hvor hårdt man træner i forhold til anbefalingerne. (kilde:motion-online.dk)

**Skema med Borgskala:**

Sundhedsstyrelsen definerer de 2 intensitetsgrader moderat og høj intensitet, som følgende:

**Moderat**:

1. Fysisk aktivitet, hvor man bliver lettere forpustet, men hvor samtale er mulig.
2. 64-74 % af maksimalpuls, eller
3. 12-13 på Borgs anstrengelsesskala

.

**Høj**:

1. fysisk aktivitet, hvor man bliver forpustet og kun kan tale i korte sætninger.
2. 77-93 % af maksimalpuls, eller
3. 14-16 på Borgs anstrengelsesskala

**Eksempler på aktiviteter med moderat og høj intensitet**

Kilde: Sundhedsstyrelsen, Center for Forebyggelse: Fysisk aktivitet – håndbog om forebyggelse og behandling. 2003

## Fordele ved at bevæge sig lidt mere

Inaktivitet og stillesiddende adfærd har helbredsmæssige konsekvenser, som er påvist ved flere studier (Stillesiddende adfærd – en helbredsrisiko, vidensråd for forebyggelse 2012).

En stor del af deltagerne i projektet lever med inaktivitet og stillesidden adfærd, så det er et vigtigt fokusområde at støtte deltagerne i at mindske stillesidning og inaktivitet, som typisk kan foregå i flere timer i træk ved eksempelvis computeraktivitet eller tv kiggeri.

Forskning peger i retning af sundhedsmæssige gevinster ved at bryde inaktivitet og stillesidning ved at ændre på små vaner i hverdagen, hvilket også fremgår hos Sundhedstyrrelsen:

- Personer, som normalt er inaktive, kan forbedre deres helbred og fysiske velvære ved at bevæge sig regelmæssigt.
- Personer i alle aldre, børn, voksne, ældre, kvinder som mænd, opnår positive fysiologiske ændringer som følge af fysisk aktivitet.
- Fysisk aktivitet behøver ikke at være anstrengende for, at man opnår sundhedsmæssige fordele.
- Fysisk aktivitet har mange positive effekter på kroppens funktioner. Virkningerne på hjerte, kredsløb og muskler har i mange år været kendt. Det er også værd at bemærke motions positive indflydelse på stofskiftet og på hormon- og immunsystemet.

Det er vigtigt at vi som livsstilscoaches er vidende om de sundhedsmæssige fordele der er ved fysisk aktivitet og også små skridt er nyttige.

Vejledning kan handle om at bevæge sig lidt hver halve time og fordele ved at bevæge de store muskelgrupper, eller mere aktivitetsorienteret at bruge trappen, gå som transport og så videre.

Det anses for sandsynligt at en stor del af deltagerne ikke er bekendt med viden om de fordele ved, at lidt fysisk aktivitet også er nyttigt og dette kan derfor anvendes som motiverende faktor.

Begrebet hverdagsmotion kan introduceres, som inspiration kan Sundhedsstyrelsens regnestykke over en inaktiv hverdag og aktiv hverdag anvendes. Her er det tydeligt at de små aktiviteter har stor nytte i det endelige energiregnskab i KJ.

## Fordele ved at træne med høj intensitet

Forskellige træningskategorier har forskellige sundhedsmæssige effekter, som illustreres i nedenstående model.

Denne viden kan livsstilscoachen anvende som motiverende faktor i arbejdet med deltagerne i projektet.

Sundhedsstyrelsen beskriver, at høj intensitet i træning kan vedligeholde eller øge konditionen og muskelstyrken. Høj intensiv træning kan være i kort varighed med korte intervaller, som anbefales til utrænede deltagere, men hvor intervallerne kan øges i takt med øgning af kondition og styrke. Det handler om at få sved på panden og mærke pulsen slå hurtigt.

**Borg skalaen** kan anvendes til vurdere om træningen er moderat eller høj intensiv.

Livsstilcoachen skal støtte deltageren i at udarbejde et **individuelt træningsprogram** hvis dette ønskes, og inspiration kan hentes på:

- Motion-online.dk
- Gomotion.dk

Forskning viser at træning med høj aerob intensitet, som for eksempel i intensive holdspil såsom fodbold og floorball, har flere positive effekter på sundhed sammenlignet med for eksempel interval løb og styrketræning. Herudover viser resultater fra Institut fra Idræt, at deltagerne i intensive holdspil oplever mindre selvoplevet belastningsgrad, sammenlignet med for eksempel styrketræning og interval løb. Dette ses i nedenstående graf.

Kilde: **Krustrup et al. 2009, Elbe et al. 2010**

Viden fra ny forskning kan anvendes i motivationsarbejdet deltagerne, og livsstilscoach vil forsøge at støtte deltagerne i træning indenfor de intensive holdspil, som udover de helbredsmæssige gevinster også byder på sociale fordele for deltagerne.

# Fysisk aktivitet og energiforbruget

Øget fysisk aktivitet i forbindelse med dagligdagens aktiviteter kan føre til et øget energiforbrug. Skemaet viser hvor meget, man typisk forbrænder ved forskellige aktiviteter.

**Se skema på næste side:**

Det er vigtigt at understrege, at øget energiforbrug ikke nødvendigvis medfører vægttab. Når man bevæger sig mindskes fedtet i kroppen, måske samtidig med at muskelmassen forøges - og derfor viser vægten det samme. En vigtig effekt af motion, også i forhold til vægtregulering er, at motion er med til at genoprette og bevare kroppens balance, så appetitten og stofskiftet kommer til at fungere bedre. Ud fra et sundhedsmæssigt synspunkt er det bedre at være fysisk aktiv med et par kilo for meget end slank og fysisk inaktiv.  Kilde: www.altomkost.dk

**Forholdsregler ved Fysisk aktivitet:**

Vi vil i CHANGE være opmærksomme på kontraindikationer ved fysisk aktivitet med deltagerne.

Alle deltagerne gennemgår en grundig lægelig undersøgelse ved baseline, og livsstilscoachen gennemgår dele af denne undersøgelse sammen med deltageren, så grundlaget for en god og sikker opstart på fysisk aktivitet er til stede.

Er der usikkerhed omkring deltagerens helbredstilstand bør dette drøftes med kollegaer i CHANGE-teamet, og evt. kan læge inddrages.

I Sundhedsstyrelsens håndbog for fysisk aktivitet, er der konkrete eksempler på kontraindikationer ved forskellige somatiske lidelser, som alle livsstilscoaches bør være opmærksomme på, inden opstart af motion.

Det er en opgave for den enkelte livsstilscoach at orientere sig i anbefalinger fra Sundhedsstyrelsen, og specielt hvis der er somatiske lidelser tilstede, at læse i ”Håndbog for fysisk aktivitet”, hvilke forholdsregler der bør tages ved opstart af fysisk aktivitet. Link: [www.sst.dk](http://www.sst.dk/)

I Håndbogen for fysisk aktivitet er der beskrivelse af hvordan fysisk aktivitet kan fungere som behandling for forskellige somatiske lidelser. Ved hver enkelt lidelse er der beskrevet baggrund, effekt af fysisk aktivitet, evidens, anbefalede træningsformer og kontraindikationer.

Nedenstående er uddrag fra udvalgte lidelser, som kunne være særligt relevante at være opmærksomme på for vores målgruppe:

**Kontraindikationer ved hypertension :**

I henhold til retningslinjer fra ACSM bør personer med blodtryk >180/105 mmHg først indlede farmakologisk behandling, inden regelmæssig fysisk aktivitet indledes (relativ kontraindikation)

ACSM anbefaler forsigtighed ved meget intensiv dynamisk træning eller styrketræning med meget tunge løft.

Ved tung styrketræning kan meget høje tryk opnås i venstre hjertekammer, hvilket kan være potentielt farligt. Særligt for patienter med venstresidig hypertrofi gælder tilbageholdenhed med kraftig styrketræning. Andre forholdsregler vil afhænge af comorbiditet.

**Kontraindikationer ved KOL:**

Ustabil angina pectoris. Træningen bør tage hensyn til konkurrerende sygdomme

**Kontraindikationer ved diabetes 2:**

Overordnet er faren ved at undlade fysisk aktivitet større end faren ved at udføre

fysisk aktivitet, men der gælder specielle forsigtighedsregler.

Fysisk aktivitet udskydes ved blodsukker >17 mmol/l, indtil det er korrigeret.

Det samme gælder ved lavt blodsukker <7 mmol/l, hvis patienten er i insulinbehandling. Ved hypertension og aktiv proliferativ retinopati frarådes hård intensitetstræning

eller træning involverende Valsalva-lignende manøvrer. Styrketræning

udføres kun med lette vægte og med lav kontraktionshastighed.

Ved neuropati og truende fodsår afstås fra kropsbærende aktiviteter. Gentagne

belastninger af neuropatiske fødder kan medføre ulcerationer og frakturer.

Løbe-/gå-bånd, lange gå-/joggingture og step øvelser frarådes, mens ikke-kropsbærende

fysisk aktivitet anbefales f.eks. cykling, svømning og roning.

Man skal være opmærksom på patienter med autonom neuroleptika, der kan

have svær iskæmi uden iskæmisymptomer (”stum iskæmi”). Disse patienter

har typisk hvile-takykardi, ortostatisme og dårlig termoregulation. Der er risiko

for pludselig hjertedød. Henvisning til kardiolog, arbejdselektrokardiogram

eller myokardiescintigrafi skal overvejes. Patienterne skal instrueres i at

undgå fysisk aktivitet under kolde/varme temperaturer samt sørge for sufficient

hydrering ved fysisk aktivitet.

**Kontraindikationer ved metabolisk syndrom:**

Ingen generelle, men træningen skal tage højde for konkurrerende sygdomme.

Ved iskæmisk hjertesygdom afstås fra intensive arbejdsintensiteter (Borg-skala

15-16). Ved hypertension udføres styrketræning med lette vægte og med lav

kontraktionshastighed.

**Kontraindikationer ved hyperlipædemi:**

Ingen generelle, men forholdsregler vil afhænge af comorbiditet. Ved hypertension

udføres styrketræning med lette vægte og med lav kontraktionshastighed

**Kontraindikationer ved skizofreni:**

Ingen generelle.

**Kontraindikationer ved overvægt:**

Ingen generelle, men træningen skal tage højde for comorbiditet. Ved iskæmisk

hjertesygdom afstås fra de korte intensive arbejdsintensiteter. Ved hypertension

udføres styrketræning med lette vægte og med lav kontraktionshastighed.

Træningsstart for personer med overvægt

1. Start blidt på motion

Planlæg en blid start på træningen, både tidsmæssigt og belastningsmæssigt. Vælg f.eks. 10 minutters træning i stedet for en hel time.

Lad være med at starte med hård belastning. I træningsmaskiner skal man ikke lægge helt så meget vægt på, som man måske godt kunne klare. Hellere en anelse i underkanten end det modsatte.

2. Bær ikke egen vægt

Vælg motionsformer, hvor man ikke bærer ens egen vægt, mens man motionerer. Det betyder, at man lettere kan træne i længere tid og har mindre risiko for skader. Det kan være:

- Cykling, hvor man sidder på sadlen
- Kondicykling
- Spinning
- Roning
- Svømning
- Vandaerobic eller andre vandaktiviteter
- Fitnesstræning, hvor man sidder i maskiner
- Gymnastik med liggende og siddende øvelser.

Gradvist kan man supplere med motion med vægtbæring, f.eks. gang, stavgang, jogging og idrætsgrene med løb.

3. Kombiner forskellige motionsformer

Ved at kombinere forskellige slags motion spreder man belastningen på flere dele af kroppen, og dermed spreder man risikoen for overbelastningsskader.

Lav kortvarig træning, gerne uden for meget vægtbæring, i flere motionsformer. Det kan være sjovt at prøve forskellige motionsformer – man kan blive overrasket over, hvad man tænder på.

4. Træn regelmæssigt

Planlæg at træne flere gange om ugen. Én gang træning om ugen er for lidt (men kan selvfølgelig være en rigtig god start), hvis man effektivt vil forebygge og behandle f.eks. KOL, hypertension, dyslipidæmi, type-2 diabetes og metabolisk-syndrom. (se evt SST anbefalinger for behandling de specifikke sygdomme).

To-tre gange motion om ugen, måske endda oftere, er passende, hvis det er de blide og ikke-vægtbærende motionsformer, man har gang i.

Skriv træningen i din kalender. Du har en aftale med dig selv, så den er vigtig…

5. Flyt fokus væk fra vægttab

Lad være med kun at tænke på at tabe dig. Ret i stedet blikket mod velvære og sundhed.

Det kan nemlig godt være, at man i starten kommer til at tage lidt på, fordi der sker nogle positive ting i ens krop. Man får måske mere muskelmasse og større blodvolumen, som øger vægten. Men det er kun godt!

6. Byg din træning gradvist op

Al motion og træning skal bygges gradvist op. Det er den måde, vi bliver stærkere på.

Med små og gradvise trin øger man belastningen. En tommelfingerregel siger max. 10 procent pr. uge. (dette gælder både trænings tid og længde (f.eks km)).

Vær opmærksom på ømheder, som bider sig fast. Ømhed er en ”rød lampe”, et tegn på overbelastning. Er man øm, må man geare lidt ned og kun træne der, hvor man ikke har smerter.

Også med små skridt når man langt i det lange løb. (Kilde: www.vorespuls.dk)

**Fysiske test ved baseline: Taljemål, BMI, konditest, Physical Activity Scale (PAS).**

| **Taljemål** |  |
| --- | --- |
| Et af de enkleste mål for om man er overvægtig er at måle sit taljemål. Samtidigt er der en tæt sammenhæng mellem taljemål og risiko for de fleste livsstilssygdomme.  Det er specielt et relevant mål fordi man ved, at især det fedt der sidder omkring maven er skadeligt for helbredet. Livvidde er ofte et bedre mål for fedttab end vægt og BMI - specielt i forbindelse med træning - da øgning af muskelmasse osv. ikke øger livvidden.  Tag et målebånd og mål stående i afslappet tilstand omkredsen 2 cm over navlehøjde.  Hvis navlen hænger lidt lavt, kan man i stedet måle i en vandret linie der findes ved at mærke sig frem til punktet midt mellem hoftebenskammen og det nederste ribben (på siden af kroppen).  **Men husk** ...  at individuel kropsbygning kan gøre at man bliver uretfærdig dømt og at den hellige grav ikke nødvendigvis er velforvaret selv om ens livvidde er i det grønne felt. Det vigtigste er stadig om man er tilstrækkeligt fysisk aktiv.   |  | **Let forøget risiko:** | **Forøget risiko:** | | --- | --- | --- | | **Mænd** | > 94 cm | > 102 cm | | **Kvinder** | > 80 cm | > 88 cm |     BMI   |  | **Fedme klassificering** | **BMI (kg/m2)** | | --- | --- | --- | | Undervægtig |  | -18.5 | | Normal |  | 18.5-24.9 | | Overvægtig |  | 25.0-29.9 | | Fedme | I | 30.0-34.9 | |  | II | 35.0-39.9 | | Ekstrem fedme | III | 40 |   BMI-tallet fortæller dig normalt ikke noget du ikke ved i forvejen og skal altid tolkes med et vist forbehold. Hvis du er meget muskuløs, eller fra naturens side er bygget ligesom et ”egetræ”, så kan det godt lade sig gøre at have et BMI på 30 uden reelt at være overvægtig. Selv hvis du ikke er specielt muskuløs og ligger i kategorien "overvægtig", så kan du sagtens have en fuldt tilfredsstillende sundhedsprofil, hvis blot du motionerer meget. Faktisk er det farligere at have et BMI på 23 og være i dårlig form, end det er at have et BMI på 28 og være i god form. Ofte er det desværre sådan, at dem der har høje BMI også er dem der motionerer mindst - og det er naturligvis en dårlig kombination. | |

Kondital

| Konditallet findes ved at tage den maksimale iltoptagelse (i ml/min) og dividere denne med kropsvægten. Konditallet angives i enheden: ml/kg/min.  Det hjælper ikke noget med en stor motor, hvis det motoren skal flytte er meget tungt. Konditallet er derfor et godt udtryk for hvor god man er til at transportere sin egen kropsvægt.  Konditallet kan variere fra 20 ml/kg/min for utrænede op til over 90 ml/kg/min for topatleter.  Nedenstående 2 tabeller gælder for almindelige mennesker der ikke dyrker idræt på konkurrenceplan: |
| --- |

Mænd Kvinder

Vurdering af overvægtiges kondital

| Konditallet er desværre ikke helt retfærdigt ift. til personer med forskellig kropsstørrelse. Store personer har sværere ved at opnå et højt kondital end små personer. Hvis man skal skal lave en sammenligning, der er mere retfærdig, skal man udregne "et kondital", hvor man dividerer iltoptagelsen med kropsvægten opløftet med eksponenten 0,73 ( ). Denne måde at beregne det på, er dog kun brugt i forbindelse med forskning. Hvis du skal vurdere konditallet for en overvægtig person, bør du kigge på artiklen "[Retfærdig vurdering af overvægtiges kondition](http://www.motion-online.dk/konditionstraening/testning/retfaerdig_vurdering_af_overvaegtiges_kondition/)  " Kilde og link: http://www.motiononline.dk/Konditabel_BMI_korrigeret.pdf  **Vedlagt skemaer med BMI korrigerede tabeller** |
| --- |

**Undersøgelse af deltagerens aktuelle fysiske aktivitetsniveau**

**Physical Activity Scale (PAS):**

|  | **Bevægelse i dagligdagen** | | | |
| --- | --- | --- | --- | --- |
|  | **Det følgende handler om, hvor meget du bevæger dig såvel på arbejde som i fritiden. Med bevægelse menes her alle aktiviteter, hvor du får rørt dine muskler og bruger dine kræfter. Der tænkes altså ikke kun på motion, idræt eller lignende.** | | | |
| Dagligt  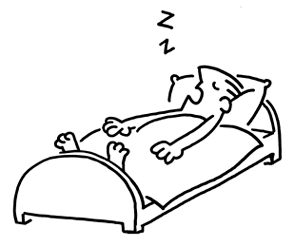 | | Hvor mange timer og minutter sover du ca. på et almindeligt hverdagsdøgn?  *(medtag middagslur)* | Timer | Minutter |
| Dagligt  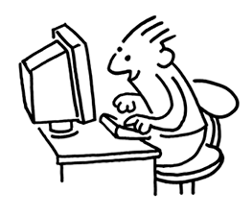  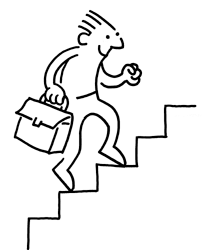 | | I **dit arbejde (eller hvis du er under uddannelse)**, hvor mange timer og minutter **om dagen** bruger du typisk på:  Stillesiddende arbejde?  Stående eller gående arbejde?  Hårdt fysisk arbejde?  *(f.eks. tunge løft eller trappegang)*  Jeg arbejder ikke/er ikke under uddannelse 🞏 | Timer  Timer  Timer | Minutter  Minutter  Minutter |
| Dagligt  **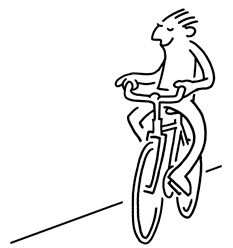** | | Hvor mange timer og minutter bruger du **dagligt** på cykling eller gang i forbindelse med transport til og fra arbejde/uddannelse?  Jeg arbejder ikke/er ikke under uddannelse 🞏 | Timer | Minutter |
| Dagligt  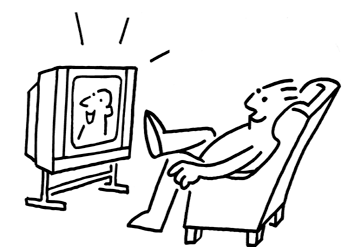 | | I **din fritid**, hvor mange timer og minutter **om dagen** bruger du ca. på at se TV, sidde ned og slappe af, læse og lytte til musik eller lignende? | Timer | Minutter |
| Ugentligt  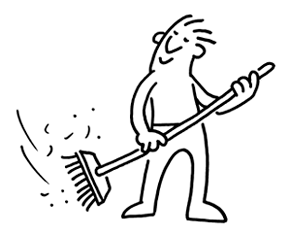 | | I **din fritid**, hvor mange timer og minutter bruger du **om ugen** på let fysisk aktivitet som f.eks. gåture, let rengøring, feje og rive i haven eller let anstrengende motion som f.eks. yoga, bowling eller lignende?  *(medtag ikke transport til og fra arbejde)* | Timer | Minutter |
| Ugentligt  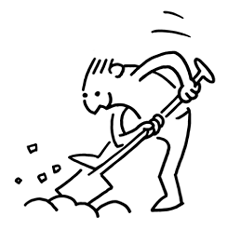 | | I **din fritid,** hvor mange timer og minutter bruger du **om ugen** på havearbejde, bære ting op af trappen eller moderat anstrengende sport som f.eks. gymnastik, svømning, cykling, styrketræning eller lignende? *(medtag ikke transport til og fra arbejde)* | Timer | Minutter |
| Ugentligt  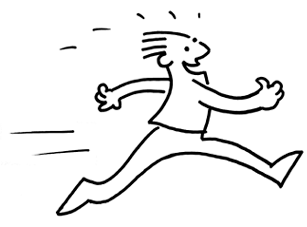 | | I **din fritid**, hvor mange timer og minutter **om ugen** bruger du på anstrengende sport og motion som f.eks. løb, jogging, fodbold, tennis, aerobic eller lignende? *(medtag ikke transport til og fra arbejde)* | Timer | Minutter |

**Skizofreni og Fysisk aktivitet**

**- Motivation og barrierer og ambivalens**

Målgruppens udfordringer i forbindelse med fysisk aktivitet:

- ***De negative symptomer*** indenfor skizofreni indebærer blandt andet initiativløshed, hvilket kan virke som en barriere i forbindelse med fysisk aktivitet. Det kan for mange være en stor udfordring at komme ud af døren.
- Der kan forekomme social tilbagetrækning og ***social fobi***  hos mange, og træning i gruppe kan både være udfordrende og for nogle føles tiltrængt for at skabe sociale kontakter.
- ***Antipsykotisk medicin*** har en sløvende effekt og giver ofte bivirkning i form af et lavere stofskifte, samt manglende mæthedsfornemmelse, hvilket kan føre til ***overvægt.*** Her udover kan medicinen give bivirkninger i form af muskelstivhed, rysten, øget tendens til krampe samt rastløshed, ufrivillige bevægelser og svimmelhed m.m.
- En stor del af patienter med skizofreni er præget af inaktivitet og ***manglende kropskontakt*** og nogle lider tillige af ***angst***. Disse to faktorer kan gøre det til en ubehagelig og angstprovokerende oplevelse, at opstarte fysisk aktivitet. Patienten skal langsomt opbygge erfaring med kroppens signaler og opøve evnen til at skelne imellem eksempelvis angstsignaler med signaler fra en krop i gang med fysisk aktivitet. Eksempelvis kan det at opleve høj puls og hurtig vejrtrækning under træning minde om et angstanfald, og dermed være en barriere for at være fysisk aktiv.
- Patientgruppen kan som en del af deres sygdomsbillede være præget af ***kropslige sansemæssige forstyrrelser*** som eksempelvis oplevelse af ekstrem kulde/varme, elektriske stød, abnorm tyngde/letheds i en del af kroppen eller hele kroppen. Det kan gøre det vanskeligt at overskue at lave fysisk aktivitet og sænker derfor lysten til dette. (Skema: Undersøgelse af kropslige sansemæssige forstyrrelser).

Disse barrierer kan fejlagtigt tolkes som manglende motivation for deltagelse i fysisk aktivitet. Undersøgelser viser dog, at patienter med psykisk sygdom er mindst lige så motiverede for at få en mere aktiv livsstil som alle andre.

Der er stor forskel på hvordan den enkelte patient er præget af sin sygdom og hvilken støtte, der er behov for i forhold til at øge sit fysiske aktivitetsniveau. Tilbuddet skal derfor være individualiseret og tilpasset den enkelte.

Kilder: A. Moltke ”Kroppens ambivalens”, www.sst.dk, Evalueringsrapport ”Fysisk aktivitet til unge med skizofreni, dec.2012., Artikel, Ph.d.stud., L.Nyboe Jacobsen ”Psykisk syge skal motionere mere for at forebygge livsstilssygdomme”.

Hovedopgaver:

Ved ambivalens omkring fysisk aktivitet

- undersøgelse af ambivalensen sammen med deltageren, samt handlemuligheder i forhold til den oplevede ambivalens.
- Støtte til struktur samt mestring i forhold til de kognitive udfordringer patienten måtte have.
- Tilpasning af fysisk aktivitet med hensyn til deltagerens fysiske og psykiske sårbarhed og ressourcer.

Motivation og barrierer i forhold til fysisk aktivitet

- Undersøgelse af motivation og barrierer i forhold til fysisk aktivitet vha. MI´s skaleringsredskab til undersøgelse af vigtighed, tro på egne evner og parathed.
- Afdæk deltagerens specifikke barrierer for fysisk aktivitet: negative symptomer, depression, angst, overvægt (det kan være vanskeligt at dyrke nogen form for motion, hvis BMI ligger over 40. Motionsmanualen, s.21), social fobi, kropslige sansemæssige forstyrrelser (Se skemaet for undersøgelse af kropslige sansemæssige forstyrrelser) m.m. Afdæk herefter specifikt patientens motivation for fysisk aktivitet.
- Ved længerevarende inaktiv livsførsel, kan det være svært at huske hvordan det føles at være fysisk aktiv. Her giver det mening, at lave aftalt og afstemt fysisk aktivitet med deltageren,så denne skaber sine egne forandringsoplevelser. Hertil kan benyttes et pædagogisk og motiverende redskab til løbende bevidstgørelse omkring den umiddelbare effekt af fysisk træning (*Subjektiv oplevelse af fysisk aktivitet)*
- Afdækning af tidligere oplevelser med fysisk træning og kendskab til motionstilbud i nærmiljøet.
- Som skrevet under "Netværkets betydning" skal det sociale netværk for den enkelte deltager undersøges mhp. at afdække den støtte, der findes her.
- Med deltagerens accept, informeres om muligheder for fysisk aktivitet i deltagerens nærmiljø, effekter af fysisk træning, og eventuelle forbehold.
- Samarbejde omkring realistisk målsætning i forhold til at arbejde med fysisk træning.

**Motion og psyke**

Denne beskrivelse er hentet fra sundhedsstyrelsens pjece, ”Motion og psyke” og gælder for den almene befolkning:

Fysisk aktivitet har positiv indvirkning på ens velvære. Når man er fysisk aktiv styrker det både den fysiske og mentale sundhed.

- Giver overskud til dagens gøremål
- Forbedrer éns humør
- Forebygger og mindsker depression
- Øger éns selvværd
- Gør det nemmere at spise sundt
- Forbedrer søvn
- Forbedrer livskvalitet
- Mindsker spændinger
- Dæmper angst og stress (får erfaring med at høj puls og at svede ikke er ”farligt”.)
- Kan danne tilgængelige rammer for social samvær/netværksdannelse
- Kan bidrage med positive oplevelser af kroppen
- Stimulerer sanseindtryk fra kroppen

Dette kan også gælde for patienter med skizofreni. Som tidligere beskrevet i ovenstående afsnit har denne målgruppe dog som følge af deres lidelse, nogle specielle udfordringer i forhold til at være fysiske aktive. Kommer patienten i gang med fysisk aktivitet kan denne opleve en positiv effekt på nogle af de symptomer, der ofte fungerer som barrierer.

Følgende er taget fra Ane Moltke bog, ”Kroppens ambivalens”, samt erfaringer fra OPUS projektet, der mundede ud i evalueringsrapporten: ”Fysisk aktivitet til unge med skizofreni” og beskriver nogle af de effekter patienter med skizofreni oplevede i et forløb med fysisk aktivitet.

- **Fra uro til ro**

- Patienter med skizofreni oplever ofte uro, hvilket både er fysisk og mentalt funderet. Der kan være tale om en følelse af uro i ben og bryst, en knude i maven og generelt anspændthed. Mental uro kan give sig udtryk i springende og hurtige tanker, hørehallucinationer eller langsomme tanker og evt. tankestop. Udover det kan uro opstå som en bivirkning til medicin. Dette kan fx. give sig udtryk i trippen med fødder eller trommen med fingre. Følelsesmæssigt kan uro give sig udtryk i irritation, nervøsitet, stress og angst. Uro var i Opus-projektet en af de væsentligste årsager til at patienterne meldte afbud. I de situationer valgte patienterne med uro blandt andet at gå i seng eller havde besvær med at komme ud af døren.

Fysisk træning kan skabe mere ro kropsligt og mentalt. Efter fysisk aktivitet kan man opleve kropslig tyngde, en behagelig træthed, føle sig afslappet, rolig og varm i kroppen. Tankerne beskrives som mindre springende, mere klare og der opleves mindre stress end før fysisk aktivitet.

- **Energi og handlekraft**

- Patienterne føler ofte lavt energiniveau og tyngde i hoved og krop. Der er nedsat kontakt med egen krop og nedsat ansigtsmimik i form af manglende eller begrænset øjenkontakt og de er verbalt fåmælt eller tavs. Fysisk aktivitet arbejder i positiv retning med patientens negative symptomer. Oplevelsen af, at mangle impuls og opleve stilstand bliver erstattet af en følelse af energi og handlekraft. Fra at have en oplevelse af tyngde vækkes kroppen ved hjælp af den fysiske aktivitet og føles efterfølgende levende, summende og bliver tydeligere for patienten. Flere patienter kan samtidig opleve at blive fysisk træt og samtidig have mere overskud. Disse to oplevelser står ikke i modsætning til hinanden. Det nyoplevede overskud hjalp flere deltagere i Opus projektet til at skabe mere mening og struktur i hverdagen. Der udover giver følelsen af energi og handlekraft patienten kontakt med en kendt identitetsfølelse fra før sygdommen. Dette kan for nogen føles som at få en side af sig selv igen.

- **Bekymring og tilfredshed.**

- Tidligere negative kropsoplevelser eller afbrudte forløb med fysisk aktivitet er med til at præge forventningerne til forestående træning og vække en vis nervøsitet og bekymring. Patienterne kan føle sig trætte, uoplagte, sløje, mærke influenzalignende symptomer og/eller have påtrængende tanker af paranoid karakter ved fx. gruppe aktivitet. Efter endt træning, vil disse forventninger og bekymringen vendes til at føle velbehag, tilfredshed og stolthed over at have gennemført, på trods af sygdommen. Fremmødet, udførelsen og den efterfølgende behagelige kropsoplevelse giver selvværd og øger troen på sig selv. De tidligere negative oplevelser af afbrudte forløb og svingende tilstand vil dog stadig præge patienterne og skabe yderligere bekymring før næste træningsgang. De positive kropsoplevelser er dog med til at styrke motivationen og overvinde barrierer ift. fysisk aktivitet.

- **Overvældelse og tryghed**

- Indtryk fra omgivelserne kan virke overvældende (musik, mange mennesker, boldspil) og kan give ubehag, skabe forvirring og en følelse af kaos, samt give hallucinationer. Kendte og overskuelige rammer og et socialt miljø præget af åbenhed og tolerance har betydning for følelsen af tryghed og samling. Udfordringerne indtil dette er opnået, samt selve træningen er en proces, hvor den enkelte oplever sig selv og sine grænser og lærer sig selv og sin krop bedre at kende. Dette er med til at skabe en følelse af tryghed.

- **Være i tankerne eller i nuet**

- Det var vanskeligt at være i kroppen og nuet under træning, når der opstod tankemylder, hørehallucinationer og forstyrrende løsrevne tanker. Samtidig oplevede patienterne at der under træning var fravær af belastende tanker og at dette fortsatte 20 min efter endt træning. Boldspil og afspænding nævnes i A. Moltkes bog, som to former for træning, hvor patienter oplevede tilstedeværelse i kroppen og nuet. Fysisk aktivitet beskrives som en pause fra tankerne.

**”Kropsfænomenologien beskriver,**

**at det at være i verden**

**er at være i verden som krop**

**og bruger vendingen,**

**`At være sin krop.´”**

**Kropslige sansemæssige forstyrrelser**

I evalueringsrapporten, ”Fysisk aktivitet til unge med skizofreni”, Dec.2011, gav næsten halvdelen af de inkluderede patienter udtryk for, at forstyrrede kropslige oplevelser medfører, at de er mindre fysisk aktive. Patienterne gav der udover udtryk for, at få betydeligt færre kropslige forstyrrelser efter interventionsperioden med fysisk aktivitet. Der er erfaringen fra klinisk praksis, at dette kan mindske patientens fysiske aktivitetsniveau og lyst til at bruge kroppen. Spørgeskemaet herunder giver et indblik i hvilke kropslige sansemæssige forstyrrelser, der kan være tale om hos patienter, der lider af skizofreni.

**Undersøgelse af kropslige sansemæssige forstyrrelser**

Scoring af spørgsmål:

0 Fravær af det respektive symptom

1 Tidligere, men ikke nuværende oplevelse af symptomet

2 En ikke generende grad af symptomet

3 En klart patologisk grad af symptomet

4 En ekstrem grad af symptomet.

**Morfologisk ændring**

”Som om”-oplevelser eller reelle oplevelser af formindskelse eller sammensnøring af enkelte

kropsdele, eller oplevelse af kropsdele eller hele kroppen bliver tyndere, kortere, større, bliver presset ned eller forsvinder.

0 Ingen oplevelse af morfologisk forandring

1 Har måske oplevet morfologiske forandringer, men kan kun huske det vagt eller kun oplevet det få gange tidligere i livet

2 Har oplevet morfologisk forandringer(mindst 3 gange), men ikke regelmæssigt og er ikke generet heraf

3 Morfologiske forandringer er tilstede dagligt i længere perioder eller hyppigt. Giver nogen gene i dagligdagen

4 Næsten konstant tilstede. Opleves stressende, som kilde til lidelse og dysfunktion

**Kropslig fremmedoplevelse**

Kroppen eller dele af den oplevelse som ændrede, fremmede, livløse, isolerede, adskilte fra

hinanden, fejlplacerede eller ikke-eksisterende.

0 Ingen kropslig fremmedoplevelse og har aldrig oplevet dette

1 Har måske oplevet kroppen som fremmed, men kan kun huske det vagt eller har kun oplevet det få gange tidligere i livet.

2 Har oplevet kroppen som fremmed/ændret, men dette har ikke været regelmæssigt, og har ikke været generet heraf. Har oplevet dette mindst 3 gange.

3 Har hyppige oplevelser af kropslig fremmedoplevelse som giver nogen gene i dagligdagen

4 Næsten konstant tilstede. Opleves stressende, angstprovokerende, og er kilde til lidelse og dysfunktion.

**Cenestiske oplevelser**

Usædvanlige sensoriske oplevelser i hele eller dele af kroppen (ofte i ekstremiteter) som

følelsesløshed, stivhed, vattet eller død følelse, usædvanlige smerteoplevelser, migrerende,

termiske, elektriske fornemmelser, oplevelse af abnorm tyngde (lethed, svæven, løft af kroppen), svimmelhed, dysæstesier.

0 Ingen cenestesier og har aldrig oplevet nogle af disse

1 Har måske haft cenestesier, men kan kun huske dette vagt eller kun oplevet det få gange tidligere i livet

2 Har haft cenestesier, men ikke vedvarende og ikke været generet heraf . Mindst oplevet dette 3 gange

3 Hyppige centestesier, som giver nogen gene i dagligdagen.

4 Næsten konstant tilstede. Opleves stressende og er kilde til lidelse og dysfunktion

**Kropslig desintegration**

Oplevelser af kropslig desintegration eller opløsning, som om kroppen falder fra hinanden, går i

stykker eller forsvinder

0 Har Ingen oplevelser af kropslig desintegration og har aldrig oplevet dette

1 Har måske oplevelser af kropslig disintegration, kan kun huske det vagt eller har kun oplevet dette få gange tidligere I livet

2 Har oplevet at kroppen gik i opløsning/faldt fra hinanden/gik i stykker eller forsvandt. Ikke generet heraf. Mindst oplevet dette 3 gange

3 Hyppige oplevelser af kropslig desintegration, som giver nogen gene/lidelse

4 Næsten konstante oplevelser af kropslig desintegration. Opleves stressende og er kilde til lidelse og dysfunktion.

**Hypokondriske ideer**

Overdreven bekymring for den kropslige sundhed og urealistiske forestillinger om fysisk sygdom

0 Ingen overdreven bekymring for den kropslige sundhed og har aldrig haft dette

1 Har måske overdreven bekymring for den kropslige sundhed og kun været optaget heraf få gange tidligere i livet

2 Mener at have en fysisk sygdom, men kan lejlighedsvis overbevises om det modsatte

3 Overdreven reaktion på forestillinger om betydningen af mindre kropsligt ubehag.

Overdreven frygt for fysisk sygdom.

4 Invaliderende eller bizarre hypokondriske ideer (fx at kroppen rådner, eller at man ikke har haft afføring i flere måneder)

**Motoriske forstyrrelser**

Oplevelser af at motorisk forstyrrelse som motorisk svækkelse, motorisk blokering,

de -automatisering af bevægelse.

0Ingen oplevelser af ændret motorisk funktion og har aldrig haft det (kroppen fungerer somvanligt)

1 Har måske haft oplevelser af at kroppen fungerer anderledes, kan kun huske dette vagt eller har kun oplevet dette få gange tidligere i livet

2 Lejlighedsvis oplevelse af forandret motorisk funktion. Mindst oplevet dette 3 gange

3 Hyppige oplevelser af motorisk forstyrrelse, som er generende i dagligdagen.

4 Næsten konstante motoriske forstyrrelser. Opleves stressende og er kilde til lidelse og dysfunktion

**Kroppen i forandring**

Dette kan eventuelt bruges som **arbejdsark** og inspiration til en samtale med deltageren omkring, hvad der sker i kroppen, når man er fysisk aktiv.

Fysisk aktivitet forebygger og behandler :

- hjertekar sygdomme
- nogle kræftformer
- type 2-diabetes.
- knogleskørhed
- forhøjet blodtryk
- overvægt

Motion vedligeholder og opbygger muskelvævet.

Større muskelmasse har flere fordele. Dels forbruges der mere energi og dels giver det en mere spændstig krop.

Fysisk aktivitet gør :

- kroppen stærkere,
- medvirker til en bedre nattesøvn,
- styrker immunforsvaret,
- stimulerer fordøjelsen, og
- holder maven i orden og regulerer appetitten.
- øger kognitionen (tænkning, erkendelse, anvendelse af viden)

Fokus på samtalen kunne være:

- Hvad klienten registrerer i sin krop i forbindelse med fysisk aktivitet.
- Fysisk aktivitet kan forandre klientens oplevelse af sig selv og sin krop.
- Fysisk aktivitet kan øge klientens kontakt med sin krop og øge kropsbevidstheden.

Spørgsmål til samtalen kunne desuden være:

*”Hvordan har du det i kroppen når du er fysisk aktiv?”*

*”Er der nogen dele af kroppen som føles ubehagelige eller hvor der er smerter?”*

*”Er der nogle dele af kroppen, hvor du oplever en forbedring?”*

*”Hvordan oplever du sult/mæthed?”*

*”Hvordan er dit energiniveau og din søvn?”*

*”Er der noget af det som du registrerer i din krop som bekymrer dig?”*

Hvis klienten er meget fokuseret på vægttab:

Eksempel*: ”Flyt fokus væk fra vægttab og ret i stedet blikket mod velvære og sundhed.*

*Det kan godt være, at du i starten kommer til at tage lidt på, fordi der sker nogle positive ting i din krop. Du får mere muskelmasse og større blodvolumen, som øger vægten. Men det er kun godt! Du vil i starten kunne opleve muskelømhed, men dette går over på et par dage og er et tegn på at musklerne arbejder/opbygges.”*

**Netværkets betydning for deltagelse i motionsaktiviteter.**

Vi vil i CHANGE have fokus på netværkets betydning for den enkelte deltager, da vi ved, at der kan være mange faktorer der motiverer mennesker til motion, og at netværket har stor betydning.

Når deltageren udtrykker ønske om, at opstarte motion, vil vi sammen undersøge og afklare om deltageren har ressourcer eller hindringer i sit netværk, som enten forstyrrer eller motiverer deltageren til motion.

Eksempler på personer i netværket der kan påvirke en deltagers udvikling i forhold til motionsaktiviteter er: Søskende, forældre, bedsteforældre, venner, venners forældre, sportsklubmedlemmer eller måske lærere.

Det er en nyttig viden, for livsstilscoachen og deltageren at afklare deltagerens ressourcer/hindringer i netværket, da ressourcer i netværket kan forstærke de positive forandringsskridt.

CHANGE kan betragtes som et intensivt tilbud i den tid vi har kontakt til deltageren, men hvis rammerne for resten af døgnets timer er barrierer for forandring må man sammen med deltageren og netværket se på mulighederne for at skabe rammer der understøtter deltagerens ønske om forandring.

Nedenstående er eksempler på, hvordan et netværk kan undersøges og skaleres, og kan anvendes i den individuelle kontakt eller som øvelse i gruppe.

**Eksempler på personer i netværket der støtter eller forstyrrer.**

**På en skala fra 0-6, hvor 6 er meget god støtte.**

**Situation:**

Hvor en deltager i CHANGE, har brug for støtte og hjælp til at komme ud og løbe

| Grupper/personer | Støtte | Forstyrrelse |
| --- | --- | --- |
| Kæreste | 6 | 1 |
| Volleyball | 2 | 0 |
| Veninder | 4 | 2 |
| Primærbehandler | 3 | 0 |
| Forældre | 2 | 0 |
| Familie | 0 | 0 |

Kilde: Opus bogen Kap. 11 og Sheldon Rose, Kognitivt gruppearbejde i praksis

Vi skal i CHANGE havde fokus på netværkets betydning for den enkelte deltager, da vi ved, at der kan være mange faktorer der motiverer mennesker til motion. Så derfor skal vi ved de første samtaler, undersøge og afklare om deltageren har ressourcer eller erfaringer i sit netværk, som kan motiverer eller fastholde deltageren og bruges i motionssammenhæng. Kap. 11 i OPUS, bogen beskriver netværkets betydning og vigtigheden af et godt pårørende samarbejde. Det er væsentligt og vigtigt, at tale med deltageren om deres holdning til gruppe/holdtræning, da vi både har praktisk erfaring og flere undersøgelser viser at mange mennesker bliver motiveret, når de er tilknyttet en motionsgruppe eller træner på et hold.

**Værdier, netværk og fysisk aktivitet**

Det er væsentligt for at ændre motionsvaner at undersøge hvilke netværk deltageren føler sig forankret i og hvilke værdier der er i ens netværk omkring fysisk aktivitet.

Føler man sig hjemme i et træningscenter eller har man sine nære kontakter på det lokale værtshus? Taler man om mennesker der træner som sunde forbilleder eller som frelste, hellige individer man ikke kan identificere sig med?

Er sport på tv en anledning til at drikke øl og spise junkfood eller er sportsstjerner helte og idealer man spejler sine håb og drømme i?
De tanker og følelser vi har afspejles i vores handlinger og som mennesker lærer vi på godt og ondt ved at spejle os i hinanden. Uanset hvilke værdier vi har som individer er det også vigtigt hvilke værdier der er i vores netværk.

At gå imod strømmen kan føles ensomt og indtil man er forankret i nye vaner (tid, motivation, vilje og erkendelse) er der kraftig risiko for tilbagefald.

Forslag til at tale med deltagere om værdier er, at anvende øvelser fra MI.

Øvelser med billeder kan anvendes (f.eks. ”Ønskekortene” fra Sundhedsstyrelsen).

**SMART-mål**

SMART-mål er punkter som livsstilscoachen kan bruge til at hjælpe deltageren med målsætning.

Med SMART-mål bliver der skabt en synlig sammenhæng mellem deltagerens målsætninger og resultater.

**SPECIFIKT:**
Første overvejelse omkring målbeskrivelsen er specifikationsgraden. Formuler målet så præcist og detaljeret som muligt.

**MÅLBART:**
Nogle glemmer, at gøre sig klart hvad ’beviset’ vil være for at de har nået deres mål. Måske fordi man fokuserer på nye problemer undervejs.

’Målbart’ handler om hvordan deltageren ved, at målet er nået. Hvad er ’beviset’ for, at målet er nået?

Er det et resultat der kan måles og vejes? Er det en følelse?

Er målet stort eller ligger langt ude i fremtiden kan det være en hjælp at dele målet op i delmål – dette kan være mere tydeligt for deltageren når man har været hele SMART-processen igennem

**ATTRAKTIVT:**

Det er vigtigt, at udforske om målet virkelig er attraktivt. Nogle gange sætter vi os mål som ikke er særlig attraktive, men som snarere er et middel til at nå andre mål. Dette kan medføre skuffelser fordi man ikke helhjertet satser på målet.

Til at illustrere hvor attraktivt målet er, kan bruges skalering fra 1-10

**REALISTISK:**

I vurderingen af om et mål er realistisk at nå, må man se på, om de nødvendige menneskelige, økonomiske og øvrige ressourcer - herunder motivation og tid - er til stede.

I afdækningen af om målet er realistisk kan bruges skalering fra 1-10. Dette kan være en øjenåbner hvis man netop har brugt samme metode i forhold til hvor attraktivt målet er og diskrepansen er stor.

Måske har deltageren brug for at erhverve flere kompetencer eller der skal iværksættes praktiske foranstaltninger før målet er realistisk.

**TIDSBESTEMT:**
Det er meget vigtigt, at der fastsættes en tydelig og overskuelig tidsramme for, hvornår et mål skal være nået. Tidsplanen er med til at konfrontere deltageren til, at tage stilling til sin vilje for at nå målet.

En variant af SMART-mål hedder SMARTE-mål (E for effekt)

Det kan være en stor motivationsfaktor, at tale om effekten af et mål. Hvad opnår deltageren ved at nå målet? Er der nogen sidegevinster?

Det er væsentligt, at deltageren kan forestille sig hvilke konsekvenser målet har. Hvis man hjælper deltageren til, at nå et mål uden at tage øvrige livsomstændigheder i betragtning kan man risikere at deltageren falder tilbage i gamle vaner eller får et nyt problem på halsen.

Det kan være en fordel at dele et mål op i mere overskuelige delmål, for herved at opnå flere succeser som f.eks. kan belønnes undervejs i forløbet.

Når man hjælper deltageren til målsætning er det vigtigt, at have MI-ånden med og have respekt for deltagerens autonomi. De udsagn som deltageren selv formulerer, er mere motiverende end hvad andre siger (BEM’s selv-perceptions teori) Coachens fornemmeste opgave er, at stille de rigtige spørgsmål …

MÅLBESKRIVELSE Dato:

SMART mål Navn:

| Navn på mål: | |
| --- | --- |
| Hvordan hænger dette mål sammen med mine andre mål og værdier i livet? | |
| S  Specifikt | Formuler målet så præcist og detaljeret som muligt. |
| M  Målbart | Hvordan kan jeg se at målet er opfyldt? Er der delmål undervejs? |
| A  Attraktivt | Hvor attraktivt er målet (1-10)? Er det et middel til at nå andre mål ? |
| R  Realistisk | Hvad taler for og i mod at målet kan nås? Hvor realistisk er målet (1-10)? |
| T  Tidsbestemt | Hvornår er målet opfyldt? (dato, tid, periode) Hvad med delmål? |
| Hvem og hvad kan støtte mig i at nå målet? - og hvordan ? | |
| Hvad er det første skridt, jeg skal tage for at nå målet? | |

**Træningsdagbog**

Træningsdagbog, ugeskema eller lignende kan kan for nogle fungere motiverende og som redskab til selvevaluering og give feedback.

Nedenfor er vedlagt et enkelt ugeskema som deltageren kan udfylde alene i mellem mødegangene (eller evt. sammen med livsstilscoachen). Det er meget individuelt for deltageren om det er meningsfuldt at registrere fysisk aktivitet og/eller træning og på hvilken måde dette kan gøres.

*(se vedlagte ugeskema)*

For nogle af deltagerene kan det at benytte en mere avanceret træningsdagbog være motiverende og lærerigt. Det er op til den enkelte livsstilscoach sammen med deltageren, at vurdere hvor detaljeret træningsdagbogen skal udfyldes.

Et af de vigtigste formål med en træningsdagbog er, at kunne kigge tilbage på sin træning og vurdere, hvad man gjorde rigtigt og hvad man evt. gjorde forkert. Et andet formål er, at den hjælper med at holde motivationen og selvdisciplinen oppe. Træningsdagbogen her (fra ”Motion-online”) er generel, og kan bruges også selv om man laver flere forskellige træningsaktiviteter.

*(se vedlagte skema)*

**Vejledning til udfyldelse af træningsdagbog:**

**1. / 2. træning: Hvis man træner mere end en gang om dagen skrives træning nr. 2 på ved: ”2. træning”.**

**Træningsform: Eksempelvis; gåtur, svømning, styrketræning, fodbold etc.**

**Tid: Effektiv træningstid i minutter.**

**Intensitet: Angives Borgskalaen (se skema) eller i procent (0-100) . 100 % er den hårdeste træning man kan forestille sig. Ved 20% får man dårligt nok sved på panden.**

**Oplagthed: Angives i procent fra 0 til 100. 100% = superoplagt, 20%= så gider man næsten ikke.**

**Vægt: Vejning foretages evt. kun en gang om ugen eller måneden.**

**Søvn: Angives i timer. Ud for f.eks. onsdag skal man skrive hvor mange timers søvn man fik natten mellem tirsdag og onsdag.**

**Bemærkninger: Skader, sygdom, stress, positive oplevelser under træning etc...**

**Træningsprogrammer**

Der findes en lang række træningsprogrammer på nettet, man kan bruge som inspiration og som med fordel kan printes ud til deltagerene. Vær dog kritisk og oplys deltageren om at programmerne kun er vejledende og ofte skal tilpasses den enkelte. Det er sjældent, at et program kan (eller skal) følges slavisk. Vi har som livsstilscoaches en vigtig opgave i at hjælpe og støtte deltageren i, at mærke efter i kroppen og reagere på de signaler kroppen sender i forhold til at forebygge overbelastning og skader.

Gode hjemmesider kan f.eks. være:

www.vorespuls.dk

www.motion-online.dk

www.gomotion.dk

**Testning**

For nogle deltagere kan det virke motiverende at få foretaget en konditest i forbindelse med opstart med fysisk aktivitet og undervejs i forløbet. Hertil kan f.eks. benyttes:

- 6 minutters gangtest
- 1,6 km gangtest
- Coopers løbetest (til deltagere som kan løbe i 12 minutter!)

En nærmere beskrivelse af de enkelte test kan findes på www.motion-online.dk. Her findes også linket til en smartphone-app med to af de ovenstående konditest.

**Motion i nærmiljøet**

**Kost og motionstilbud - Århus Kommune:**

**Idræt på banen-**

**Socialpsykiatrien Katrinebjergvej**

Træningscenteret 81

8200 Århus N

tlf.: 87132150

www.katrinebjergvej.dk

Kontaktperson Niels Iversen : 28870166

http://www.idraet-paa-banen.dk/

Ingen visitation -forskellige idrætsaktiviteter til udsatte voksne og evt. ledsagere- eget styrketræningslokale, desuden tilbud om fællesspisning, gågruppe, bordtennis etc.

**Fysioterapeutisk ambulatorium**

Århus Universitetshospital Risskov.

Skovagervej 2

8240 Risskov

tlf: 78471580.

Træningscenteret motionshulens åbningstider:

man-ons - fre 8.30 -13.45

tirs og tors 8.30 -16.00

Søndag 9.00 -12. 00

Desuden ”mild bevægelse” og afspænding.

**Frem-aktiv:**

Idrætstilbud for personer med misbrug, hjemløse, psykisk syge og andre udsatte borgere.

Henvisning er ikke nødvendig. Borgeren skal ikke ringe i forvejen, men møder bare op på et af de nedenstående steder.

- Christiansbjerghallen
- Århus Bowlinghal
- Møllestien
- Gellerup
- Yderligere oplysninger

 tlf: 51 57 63 22/ 51 57 61 22

http://www.aarhus.dk/sitecore/content/Subsites/FolkesundhedAarhus/Home.aspx

https://www.sundhed.dk/sundhedsfaglig/sundhedstilbud/region-midtjylland/kommuner/aarhus/traening/idraetstilbud-for-socialt-udsatte-borgere/

**Dalgasskolen:**

Lilleskole for voksne

Dalgas avenue 12-8000 århus C

Tlf: 86124840

Mail: dalgas@dalgasskolen-aarhus.dk

http://www.dalgasskolen-aarhus.dk/

kontaktperson ang. kost og motion : Ulla Rasmussen

”Sund kost”gruppe, løbegruppe, motion, styrketræning, dans...

**Kultur og kontaktsted Kragelund**

Dybbrovej 21,8270-Højbjerg

tlf: 87131848

www.kragelund.dk

Kontaktperson Gitte mobil 51335702

Tilbud om motion, styrketræning, gåture, køkkengruppe (fællesspisning)

http://www.aarhus.dk/da/borger/kultur-idraet-og-fritid/Aktivitetstilbud/Psykiatri-og-udsatte/Kultur-og-Kontaktsted-Kragelund.aspx

**IDA-idrætsdaghøjskole**

Gøteborgalle 9

8200 Århus N

TLF: 86165977

www.idaa.dk, info@idaa.dk

Dagtilbud/undervisning rettet mod uddannelse eller jobsøgning.

Mange tilbud om Idræt og sund livsstil.

**Aktivitetscenter og kontaktsted Annagade**

Skt. Annagade skole

8000 Århus C

TLF: 86192644

mail: annagade@msb.aarhus.dk

www. aktivitetescenter-annagade.dk

Idræt på banen 28870106

Åbent tilbud uden visitation,evt. med ledsager.

Tilbud om svømning, gåture, fællesspisning, køkkengruppe etc...

**Århus Kommune-**

**Folkesundhed Århus**

Ceres alle 13

8000 Århus C.

tlf: 87134035

folkesundhed@mso.aarhus.dk

www.folkesundhedAarhus.dk

Tilbud om sund livsstil, rygestop (også individuelt) , div. træningsforløb , forløb omkring KOL,diabetes og hjerte/kar sygdomme. Alle kan møde op og lave en aftale med sundhedskonsulent.

**Klostergadecenteret:**

Primært tilbud til ældre, div. kost og motionstilbud. (varmtvandsbassin , møllestien)

Klostergade 37,  8000 Aarhus C, Danmark
8619 2530

|  |
| --- |

**FO århus**

Frederiksgade 78c

8000 Århus C.

td@fo-aarhus.dk

Kontakt ved oprettelse af kostgrupper :Torben Dreier , skoleleder

87464530

**http://www.fo-aarhus-dk.**

**FOF i Århus:**

Mulighed for kurser til mennesker med psykisk/fysisk handicap, små hold og individuelle hensyn.

| Fredensgade 36,  8000 Aarhus C, Danmark Tlf.8612 2955 |
| --- |

http://www.fof.dk/Kurser.aspx?enhed=2&menu=11

**DGI-huset.:**

Værkmestergade 17
8000 Århus C
86 18 00 88
[info@dgi-huset.dk](mailto:info@dgi-huset.dk)

**Mange** forskellige motions og trænings tilbud ( også nogle få hensynstagende)

http://www.dgi-huset.dk/content/view/79/144/

**CSV-Aarhus:**

Undervisning for unge og voksne med psykiske vanskeligheder.

Aarhus kommune . Nørre Allé 31 . 8000 Århus C . Telefon 86 20 79 79 . Mail: [csv@msb.aarhus.dk](../../../../../Global/Contactform)

Tilbud om motion og bevægelse.

**Silkeborg:**

Idræt om dagen.

Idræt for brugere af psykiatrien: yoga , volleyball, zumba, gåture, bowling.

http://www.if-silkesind.dk/

**Socialpsykiatri syddjurs:**

http://www.syddjurs.dk/indhold/aktivitetstilbud-i-social-psykiatrien

**Gode hjemmesider:**

http://www.sind.dk/aarhus_amt1

Dansk idrætsforbund:

www.dif.dk

www. hjerteforeningen.dk

www.cancer.dk

www.diabetes.dk

www.sundhed.dk

**Fysisk aktivitet i grupper i CHANGE**

**Baggrund**
Der kan være mange fordele ved at lave gruppetilbud med fysisk aktivitet til deltagerne i CHANGE:

- Man kan rumme flere deltagere på en gang.
- Faste ugentlige tilbud kan hjælpe med at give struktur i hverdagen.
- Der kan opstå en gruppedynamik hvor tilknytningsforhold og tryghed danner ramme om fysisk udfoldelse. Deltagerne lærer hinanden at kende hvor det ikke er sygdom der er i fokus, eller de begrænsninger sygdommen giver. Man bliver i stedet for en del af et fællesskab med et positivt indhold og får en ny identitet som ’en der træner’.
- Deltageren møder andre som oplever lignende problematikker og -udfordringer, hvilket kan mindske følelsen af, at være alene og give håb om forandring i takt med at de oplever hvorledes andre mestrer udfordringer. Deltagerne kan på den måde opleve og bruge hinanden som rollemodeller.
- Netværksdannelse (ringe/hente aftaler m.m.) og forventninger fra gruppens øvrige deltagere om fremmøde. Det kan fungere som støtte til at møde op til aktiviteten. Der kan på sigt opstå mulighed for at etablere makkerordninger/trænings”buddies” udover de faste gruppetilbud og forhåbentlig udover CHANGE interventionernes løbetid.

**Struktur og rammer**

Struktur er vigtig i forhold til motionstilbud i gruppe. Dette kan både være til eksisterende idrætstilbud i byen, eller hvis CHANGE teamet etablerer et idræts- eller motionstilbud.
Deltagerne skal være bekendte med strukturen for aktiviteten. Struktur og forudsigelighed er vigtig, for at minimere ambivalens og for at negative symptomer som apati og meningsløshed gør det for svært for deltageren at komme af sted til aktiviteterne.

Gør deltagerne opmærksomme på hvad de forventes at medbringe – f.eks. indendørs sko i sportshaller, drikkedunk, håndklæde, skiftetøj m.m.

Det er vigtigt, at deltagerne når det er muligt, involveres i indholdet og rammerne for aktiviteten, for herved at føle ejerskab samt gøre aktiviteten meningsfuld og vedkommende. Herudover kan deltagerne eventuelt være med til at formulere en gruppekontrakt: At i talesætte forventninger om engagement, disciplin, fremmøde m.m.

**3. halvleg**

Den 3. halvleg giver mulighed for at deltagerne at sætte ord på dagens oplevelse af aktiviteten. Spørgsmål kunne her være; Hvad skete der i kroppen? Hvordan føltes og mærkes det? Hvad var godt ved at du kom i dag?
Denne ’evaluering’ er vigtig i forhold til, at deltagerne formulerer udsagn, der gør det nemmere at huske hvorfor man prioriterer at komme til træningen – også de dage hvor det er svært og det regner udenfor. 3. halvleg kan også indeholde, at man drikker noget sammen efter aktiviteten eller spiser et stykke frugt.

**Intensitet og niveauforskelle**
Gruppeaktiviteter der har et element af leg gør, at de fysiske udfordringer/anstrengelser træder i baggrunden. Ved aktiviteter som fodbold er der bedst erfaringer med homogene hold (mænd og kvinder for sig). Har man blandede hold, kan man opnå samme intensitet ved at spille floorball. Der er deltagernes fysik ikke på samme måde en faktor, der er ingen kropstacklinger, men stadig leg, hold og boldspil.
Niveauforskelle kan gøre visse former for aktiviteter svære at gennemføre i en gruppe. Det er svært at få volleyball eller badminton til at fungere hvis deltagernes færdigheder er meget forskellige.
Under fysisk aktivitet er vi i samme båd og et ulige forhold mellem coach og deltager udviskes. Det kan være en succesoplevelse for en deltager, at vise holdet hvordan man udfører en del af spillet (server m.m.) hvor de vanlige roller er byttet om.

**Motivation**Det er forskelligt hvad der motiverer os som individer til at være fysisk aktive.
Det kan være resultater der kan måles, en følelse af velvære, det sociale aspekt, en bevidsthed om at være sund. Det kan også være muligheden for at købe gear og gadgets, deltage i konkurrencer, eller en vej til andre mål, f.eks. vægttab eller som hjælp til at opbygge sociale færdigheder.

Motivationsfaktorerne er vigtige at have med i betragtning, når man tilbyder fysisk aktivitet i grupper. Deltagelse kræver, at man i en vis grad kan og er motiveret for at indgå i sociale sammenhænge.

I grupper/holdspil kan konkurrenceelementet udleves ved at deltage i turneringer. Holdånden kan styrkes ved at have pokalhylder, hente medaljer hjem eller have fælles klubtøj.

**Events**Changeprotokollen nævner deltagelse i events og udflugter som en motiverende faktor. Events kan forstås som allerede eksisterende arrangementer i psykiatrisk regi (Sund By løb i København, Sct. Hans Løb i Roskilde, Psykiatriløb Risskov, Idrætsfestival for Sindslidende i Vejle mm.)
Her er der mulighed for individuelle mål (at gennemføre på bestemt tid mm.) men også ofte præmier til den organisation/klub der har flest gennemførende deltagere.
Man kan også tilmelde sig turneringer via DAI (Dansk Arbejder Idræt) og lignende paraplyorganisationer. Gennem idrætsforeninger for sindslidende er der også mange turneringer og stævner.

I forhold til CHANGE deltagere er det relevant at lave fællesture, der kan give anderledes oplevelser i forhold til fysisk aktivitet. F.eks. en fast dag om måneden hvor coaches på skift arrangerer events for alle de CHANGE deltagere der har lyst. Det kan være tur i skøjtehallen, ridetur, cykelture, foredrag med inspirerende rollemodeller eller andre arrangementer med et vist socialt indhold.
Formålet er, at inspirere deltagerne til at være opsøgende i forhold til aktiviteter der ligger udenfor rammerne af hvad CHANGE interventionen tilbyder.

**Deltagernes tidligere erfaringer med fysisk aktivitet i grupper**I den individuelle kontakt med deltageren spørger livsstilscoachen ind til tidligere succesoplevelser med fysisk aktivitet. Tilbud om fysisk aktivitet i grupper kan således både vække gode og dårlige minder. Hvis deltageren har dårlig erfaring med fysisk aktivitet kan det være nødvendigt at arbejde individuelt med deltageren før denne er i stand til at forpligte sig til fysisk aktivitet i grupper.

**Skizofreni og sociale sammenhænge**Sociale færdigheder kan deles op i forskellige områder

1. Nonverbale færdigheder
2. Grundlæggende verbale færdigheder
3. Højere udviklede verbale færdigheder
4. Selvbeskyttende færdigheder

Det er ikke meningen, at vi som coaches skal lave social færdighedstræning, men man bør være bevidst om, at kognitionen, hukommelse og de eksekutive funktioner kan være påvirkede af skizofreni sygdommen. Har man ikke tilstrækkelige sociale færdigheder, kan det være svært at have øjenkontakt, tale klart og tydeligt, indgå i sociale sammenhænge og styre sine impulser i forhold til vrede, kritik m.m. Fysisk aktivitet i grupper kan "trigge" situationer hvor deltagerne kan opleve manglende sociale færdigheder og gøre at man reagerer uhensigtsmæssigt eller i affekt.
Det er en god idé på forhånd at aftale spilleregler i gruppen. Et par eksempler på dette kunne være, at man giver besked hvis man forlader aktiviteten og at der er plads til alle uanset engagement og evner.

Litteratur:

- http://www.sst.dk/publ:Fysisk aktivitet – håndbog om forebyggelse og behandling, Fysisk aktivitet i psykiatrien, Erfaringer fra et landsdækkende implementeringsprojekt, 2009, Motion og psyke, en vejledning for større psykisk velvære. - Protokol: Projekt CHANGE: Det gælder livet.- Isaksen, Dureta et al, (2010, )Bachelorprojekt: 'Det er sådan et frirum’ – fysisk aktivitet i psykiatrien - 2010:
https://www.ucviden.dk/student-portal/da/studentprojects/det-er-saadan-et-frirum(71270ae5-bd33-447b-99dd-853c715d3336).htm - Moltke, Ane: Kroppens ambivalens, 2010 - Oestrich, Irene H.:Selvtillidstræning og udvikling af sociale færdigheder - Mod angst og psykiske vanskeligheder, psykiatrifondens forlag, 1998. - Gerlach, Jes: Skizofreni og andre psykoser, 2011, Psykiatri fondens forlag.

**Vidensbank med referencer (SST, DSAM, forsknings artikler, hjemmesider m.m)**

Litteratur, baggrund, artikler mm.:

Motivationssamtalen; William R. Miller & Stephen Rollnick, Hans Reitzels Forlag, København 2004
Fysisk aktivitet – håndbog om forebyggelse og behandling, Sundhedsstyrelsen

Fysisk aktivitet i psykiatrien, Sundhedsstyrelsen SST (Undervisningsmateriale 2006) og
Fysisk aktivitet i psykiatrien – erfaringer fra et landsdækkende implementeringsprojekt (www.sst.dk) juli 2009

Pjece: Motion og psyke, Sundhedsstyrelsen

CHANGE protokol

Forandringscirklen

Den kognitive diamant

Krustrup, Peter …

Kost og motion til psykisk syge, Bispebjerg Hospital (manual)

Moltke, Ane; Kroppens ambivalens

Oestrich, Irene H.; Selvtillidstræning og udvikling af sociale færdigheder (Psykiatri fondens forlag)

Gerlach, Jes; Skizofreni og andre psykoser (Psykiatri fondens forlag 2011)

**Hjemmesider:**

http://www.sind.dk/aarhus_amt1

Dansk idrætsforbund:

www.dif.dk

www. hjerteforeningen.dk

www.cancer.dk

www.diabetes.dk

www.sundhed.dk

www.motion-online.dk

www.vorespuls.dk

www.gomotion.dk

www.dai.dk
